# Mechanochemical Synthesis of *α*‐halo Alkylboronic Esters

**DOI:** 10.1002/advs.202404071

**Published:** 2024-07-03

**Authors:** Yunyi Zhao, Zekun Yang, Xin Wang, Qinchun Kang, Bobo Wang, Tianle Wu, Hao Lei, Peile Ma, Wenqiang Su, Siyuan Wang, Zhiqiang Wu, Xinsong Huang, Chunying Fan, Xiaofeng Wei

**Affiliations:** ^1^ School of Pharmacy Xi'an Jiaotong University No.76, Yanta West Road Xi'an Shaanxi 710061 P. R. China; ^2^ Key Laboratory of Environment and Genes Related to Diseases (Xi'an Jiaotong University) Ministry of Education Xi'an Shaanxi 710061 P. R. China; ^3^ Ningxia Jinghong Technology Co., Ltd. No. 98 Huihong District, Shizuishan Economic and Technological Development Zone Shizuishan Ningxia 753000 P. R. China; ^4^ Department of Pathogenic Microbiology and Immunology School of Basic Medical Sciences Xi'an Jiaotong University Yanta District Xi'an Shaanxi 710061 P. R. China; ^5^ Department of Medicinal Chemistry College of Pharmacy Shenzhen Technology University Shenzhen 518118 P. R. China

**Keywords:** *α*‐halo alkylboronic esters, external catalyst‐free, halogenation, mechanochemical, radical relayed

## Abstract

*α*‐halo alkylboronic esters, acting as ambiphilic synthons, play a pivotal role as versatile intermediates in fields like pharmaceutical science and organic chemistry. The sequential transformation of carbon–boron and carbon–halogen bonds into a broad range of carbon–X bonds allows for programmable bond formation, facilitating the incorporation of multiple substituents at a single position and streamlining the synthesis of complex molecules. Nevertheless, the synthetic potential of these compounds is constrained by limited reaction patterns. Additionally, the conventional methods often necessitate the use of bulk toxic solvents, exhibit sensitivity to air/moisture, rely on expensive metal catalysts, and involve extended reaction times. In this report, a ball milling technique is introduced that overcomes these limitations, enabling the external catalyst‐free multicomponent coupling of aryl diazonium salts, alkenes, and simple metal halides. This approach offers a general and straightforward method for obtaining a diverse array of *α*‐halo alkylboronic esters, thereby paving the way for the extensive utilization of these synthons in the synthesis of fine chemicals.

## Graphical abstract

An external catalyst‐free and solvent‐less mechanochemical protocol is developed for the direct synthesis of *α*‐halo alkylboronic esters. This reaction features mild conditions, including short reaction times and the absence of both solvents and external catalysts. The simplicity of the procedure makes this methodology both sustainable and cost‐effective.

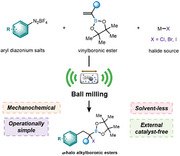

## 1 Introduction

**Figure 1:**
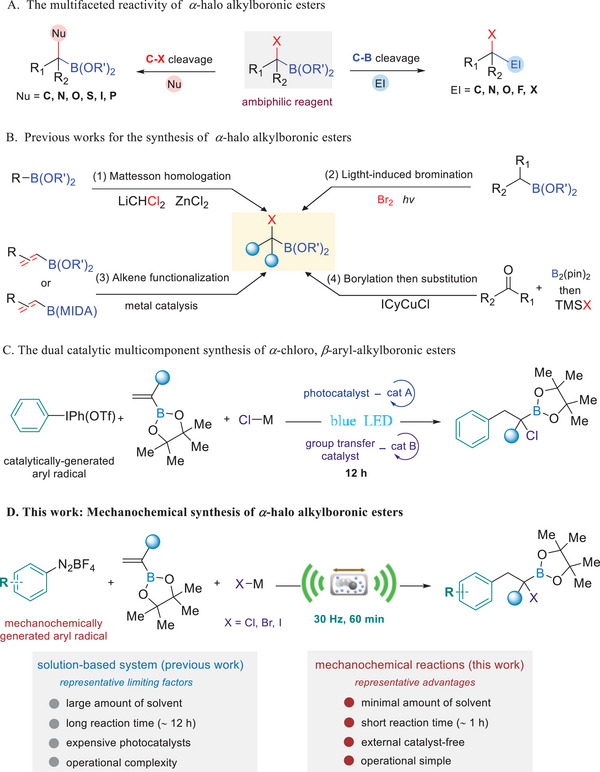
Toward a new strategy for the synthesis of *α*‐halo alkylboronic esters. A) The multifaceted reactivity of *α*‐halo alkylboronic esters. B) Previous works for the synthesis *α*‐halo alkylboronic esters. C) Example of the synthesis of *α*‐chloro, *β*‐aryl‐alkylboronic esters via visible‐light‐mediated dual catalysis. D) Mechanochemical synthesis of *α*‐halo alkylboronic esters.

The creation of platform molecules with versatile transformation potential is pivotal in organic chemistry. This advancement holds the promise of inspiring innovative retrosynthetic strategies, fundamentally reshaping the terrain of complex molecular synthesis. Consequently, chemists persist in actively seeking methods for the facile synthesis of these molecules from readily available chemicals in an operationally simple and catalyst‐free manner. As a representative example, organoboron compounds play a crucial role in various fields, spanning from materials science to biochemistry and organic synthesis.^[^ ^1^, ^2^, ^3^ ^]^ They have been extensively explored, investigated, and utilized as reactants in well‐known processes, including the hydroboration of olefins^[^ ^4^ ^]^ and the Suzuki cross‐coupling.^[^ ^5^ ^]^ Introducing a transformable halogen atom on the same carbon produces *α*‐halo alkylboronic esters, acting as multifunctional synthons in organic synthesis.^[^ ^6^, ^7^, ^8^, ^9^, ^10^, ^11^ ^]^ *α*‐halo alkylboronic esters, as a unique geminal difunctional skeleton, possess both nucleophilic and electrophilic properties. Their transformations have been utilized to expeditiously construct valuable complex molecules (**Figure** **1**A).^[^ ^12^, ^13^, ^14^ ^]^ Various methods for synthesizing *α*‐halo alkylboronic esters have been reported to date (Figure 1B). Traditionally, Matteson and co‐workers have developed a powerful, versatile strategy for their synthesis through the reaction of alkyl‐(or aryl‐) boronic esters with (dichloromethy)lithium (LiCHCl_2_).^[^ ^15^, ^16^ ^]^ However, the need for low temperatures and the intrinsic high basicity of LiCHCl_2_ limit its widespread synthetic applications. Some success was achieved by using photochemical radical reaction conditions,^[^ ^17^, ^18^, ^19^ ^]^ but only bromination reaction is feasible and the substrate scope was mainly limited to secondary alkylboronate esters. In the past decade, some new methods have been developed to access *α*‐halo alkylboronic esters through the bifunctionalization of boryl alkenes. For example, Casar and co‐workers reported iridium‐catalyzed hydrogenation of *α*‐haloalkenyl boronic esters.^[^ ^20^, ^21^, ^22^ ^]^ Ueda, Song, and Hull forged *α*‐halo alkylboronic esters through atom transfer radical addition to vinyl boronic esters.^[^ ^23^, ^24^, ^25^ ^]^ Furthermore, Aggarwal and co‐workers have made significant contributions to the field by pioneering alternative methods to direct Matteson chemistry. Their approaches, involving 1,2‐rearrangements through boronate intermediates, have emerged as potent strategies for synthesizing complex molecules.^[^ ^26^, ^27^, ^28^, ^29^, ^30^, ^31^ ^]^ Recently, Xu et al. developed a modular method for converting carbonyl compounds into *α*‐halo alkylboronic esters via a borylation/halogen substitution sequence.^[^ ^32^ ^]^ Burke and colleagues have elucidated the ability of coordinatively saturated MIDA (*N*‐methyliminodiacetic acid) and TIDA (tris(trimethylsilyl)amine) boronates to stabilize secondary alkyl radicals through *σ*_B‐N_ hyperconjugation. This stabilization mechanism facilitates site‐selective C–H bromination, leading to the generation of *α*‐bromoboronates.^[^ ^33^ ^]^ Lastly, *gem*‐diboron reagents have attracted extensive attention in the field of chemistry in recent years due to their special properties and accessibility.^[^ ^34^, ^35^, ^36^, ^37^ ^]^ Song and co‐workers reported the synthesis of *α*‐chloro or bromo boronates by the treatment of *gem*‐diborylalkanes with *^n^* BuLi reagent via in situ formed tetracoordinate boron species.^[^ ^38^ ^]^ In 2023, Gaunt et al. successfully synthesized *α*‐chloro alkylboronic esters via additions of aryl group and chlorine atom across the vinyl boronic ester enabled by a visible‐light‐mediated dual catalytic system (Figure 1C).^[^ ^39^ ^]^ However, this process required an expensive photocatalyst and group transfer catalyst, long reaction time (12 h), large amounts of organic solvents (0.2 m), and operational complexity.

Recently, the utilization of the mechanochemical strategy has emerged as a promising avenue for organic transformations.^[^ ^40^, ^41^, ^42^, ^43^, ^44^, ^45^, ^46^, ^47^, ^48^, ^49^, ^50^, ^51^, ^52^, ^53^, ^54^ ^]^ Its notable advantages encompass the removal of potentially harmful organic solvents and the alleviation of harsh reaction conditions. This approach also leads to shorter reaction times and simplifies the overall operational processes. Various reactions, including arylation, borylation, atom‐transfer radical cyclization, trifluoromethylation, and fluorination, have demonstrated successful outcomes when conducted in the solid state,^[^ ^54^, ^55^, ^56^, ^57^, ^58^, ^59^ ^]^ although external oxidants,^[^ ^60^, ^61^ ^]^ reductants^[^ ^62^ ^]^ and piezoelectric materials^[^ ^54^, ^58^, ^63^, ^64^ ^]^ are generally required for substrate activation. Breaking stable covalent bonds in small molecules using mechanical force to generate radicals presents a challenging problem.^[^ ^65^, ^66^ ^]^ As far as our knowledge extends, a general and straightforward method for obtaining a diverse array of *α*‐halo alkylboronic esters in solid‐state remains largely unexplored, despite its high potential in both academic and industrial contexts. Herein, we report the external catalyst‐free and solvent‐less mechanochemical synthesis of *α*‐halo alkylboronic esters through a multicomponent coupling process involving aryl diazonium salts, alkenes, and a simple metal halide under ball‐milling conditions (Figure 1D). In this transformation, aryl radicals are generated by mechanical C–N bond breaking of aryldiazonium salt in the presence of NaCl, eliminating the need for an external electron transfer mediator. The reaction demonstrates wide applicability with vinyl‐boronic ester and aryl diazonium salt components, yielding versatile intermediate products of *α*‐halo alkylboronic esters for the sequential conversion of C–B bond and C–halogen bond into C–C, C–N, C–O bonds,^[^ ^14^, ^24^, ^25^, ^67^, ^68^, ^69^ ^]^ highlighting the significant potential in complex molecule synthesis.

## 2 Results and Discussion

We initially conducted a study to optimize the conditions for mechanochemical synthesis of *α*‐halo alkylboronic esters. Using the aryl diazonium salts **1a** and vinylboronic ester **2a** with commercially available potassium chloride (**Table** **1**), all reactions were performed in a Retsch MM400 mixer mill (stainless‐steel milling jar: 1.5 mL; stainless‐steel ball: 5 mm diameter, see Figures S1 and S2, Supporting Information). We were pleased to achieve a 44% yield of desired *α*‐chloro alkylboronic esters **3** in the initial reaction without the addition of an external metal‐catalyst and liquid‐assisted grinding (LAG) (entry 1). Subsequently, we aimed to enhance reactivity by employing LAG, involving the addition of a substoichiometric amount of liquid.^[^ ^70^, ^71^ ^]^ In all LAG reactions, the ratio of liquid additive (microliters) to reactant (milligrams) was 0.2. The use of acetonitrile (MeCN) as the LAG additive improved the yield of **3**, while other common solvents like DCM, MeOH, THF or *N,N*‐dimethylformamide (DMF) showed minimal or no improvement (entries 2–6). Increasing the amounts of LAG and potassium chloride did not further enhance the yield of **3** (entries 7–9). For better mass efficiency, we tested a lower loading of KCl and vinylboronic ester and found that 1.5 equivalents of KCl and **2a** resulted in an excellent yield of 82% (entries 10–11). However, shortening the reaction time to 0.5 h led to a decreased yield (72%, entry 12). Subsequently, we optimized other metal chlorides (LiCl, NaCl and CsCl) (entries 13–15), and NaCl provided almost quantitative conversion of **3** (99%, entry 16). Similarly, not only *α*‐chloro alkylboronic esters, but also the analogous *α*‐bromo‐ and *α*‐iodo alkylboronic esters were readily obtained by using the corresponding halide source (entries 18–19). Remarkably, even when the reaction was conducted in ambient air, there was only a slight reduction in reactivity (84%, entry 20), highlighting the practicality of our protocol. In contrast, solution‐based conditions failed to yield product **3** (entry 21), even with an extended reaction time (entry 22), emphasizing the indispensable role of the mechanical energy supplied by ball milling in this radical relayed process.

**Table 1:** Optimization of the reaction conditions^a)^.

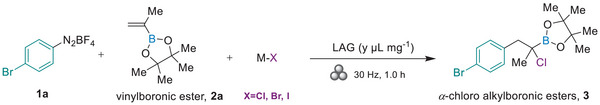				
Entry	M‐X (x eq.)	Ratio (1a:2a)	LAG [y µL mg^−1^]	Yield [%] ^b^ ^)^
1	KCl (2)	1:2	–	44
2	KCl (2)	1:2	DCM (0.2)	24
3	KCl (2)	1:2	MeOH (0.2)	50
4	KCl (2)	1:2	THF (0.2)	53
5	KCl (2)	1:2	DMF (0.2)	68
6	KCl (2)	1:2	MeCN (0.2)	83
7	KCl (2)	1:2	MeCN (0.3)	83
8	KCl (2)	1:2	MeCN (0.5)	75
9	KCl (5)	1:2	MeCN (0.3)	80
10	KCl (1.5)	1:1.5	MeCN (0.2)	82
11	KCl (1.2)	1:1.2	MeCN (0.2)	68
12^c)^	KCl (1.5)	1:1.5	MeCN (0.2)	72
13	LiCl (1.5)	1:1.5	MeCN (0.2)	77
14	NaCl (1.5)	1:1.5	MeCN (0.2)	93
15	CsCl (1.5)	1:1.5	MeCN (0.2)	99
**16**	**NaCl (1.5)**	**1.5:1**	**MeCN (0.2)**	**99**
17	NaCl (1.5)	1.2:1	MeCN (0.2)	98
18	NaBr (1.5)	1.5:1	MeCN (0.2)	99
19	NaI (1.5)	1.5:1	MeCN (0.2)	43
20^d)^	NaCl (1.5)	1.5:1	MeCN (0.2)	84
21^e)^	NaCl (1.5)	1.5:1	MeCN (0.2)	0
22^f)^	NaCl (1.5)	1.5:1	MeCN (0.2)	Trace

^a)^ General reaction conditions: unless otherwise noted, all reactions were performed on 0.2 mmol scale, **1a** (xx mmol), **2a** (xx mmol), MX (x eq.) and LAG (y µL mg^−1^) were added in a 1.5 mL stainless‐steel milling jar with a stainless‐steel ball (diameter, 5 mm) under nitrogen atmosphere and milled at 30 Hz for 1 h;

^b)^ ^1^H NMR yield with dibromomethane (CH_2_Br_2_) as the internal standard.

^c)^ 0.5 h.

^d)^ Under the air atmosphere.

^e)^ 1.0 h in solution.

^f)^ 24 h in solution.

**Figure 2:**
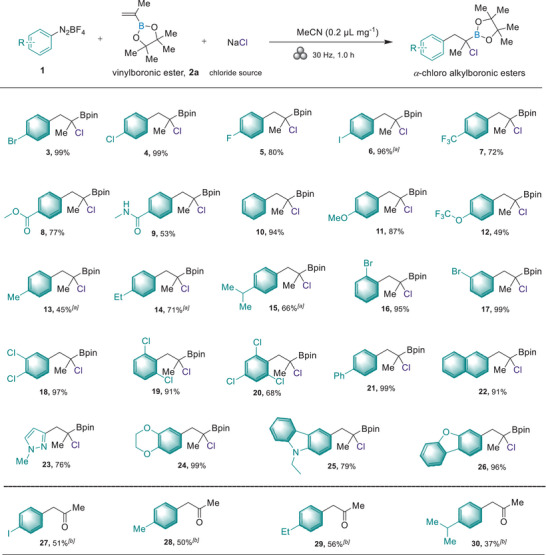
Substrate scope of aryl diazonium salts. Reaction conditions: unless otherwise noted, **1** (0.3 mmol), **2a** (0.2 mmol), NaCl (1.5 eq.) and LAG (0.2 µL mg^−1^) were added in a 1.5 mL stainless‐steel milling jar with a stainless‐steel ball (diameter, 5 mm) under nitrogen atmosphere and milled at 30 Hz for 1 h. [a] ^1^H NMR yield with CH_2_Br_2_ as the internal standard. [b] Products were oxidated by NaBO_3_•4H_2_O (7.0 eq.) in THF/H_2_O for 4 h, isolated yields.

With the optimized reaction conditions in hand, we explored the scope of the reaction in the aryl component using a range of substituted diazonium salts, which can be easily prepared from the corresponding arylamines (**Figure** **2**; Figure S3, Supporting Information). Halogen groups, such as bromide (**3**), chloride (**4**), fluoride (**5**), iodide (**6**), as well as electron‐deficient trifluoromethyl (**7**), ester (**8**) and amide (**9**) groups, were well‐tolerated, yielding the corresponding *α*‐chloro alkylboronic esters in excellent yields (up to 99%). Additionally, the halogen atoms (e.g., **3**–**4**, **6**) could be utilized for further synthetic manipulation through transition metal‐catalyzed cross‐coupling reactions. Aryl diazonium salts bearing electron‐donating groups (methoxy, trifluoromethoxy, methyl, ethyl, and isopropyl) appeared to decrease the reaction's efficiency, resulting in reduced yields (**11‐15**: 45%−87%). In addition to *para*‐substituted aryl diazonium salts, the *meta*‐ and *ortho*‐substituted benzenes successfully reacted with vinyl‐boronic ester, yielding the corresponding products in good yields (**16**: 95%; **17**: 99%). Furthermore, sterically hindered aryl diazonium salts containing di‐ and tri‐substituted phenyl groups were efficiently transformed into the *α*‐chloro alkylboronic esters derivatives with fair to good yields (**18‐20**). Notably, polyaromatic groups, often considered challenging substrates in solution‐based conditions due to their limited solubility, effectively produced the desired products (**21**: 99%; **22**: 91%). Heterocycles, which are important in medicinal chemistry, were also explored. Several aryl diazonium salts containing heterocycles, such as a 1‐methyl‐1*H*‐pyrazole (**23**), 2,3‐dihydrobenzo[*b*][1,4]dioxine (**24**), 9‐ethyl‐9*H*‐carbazole (**25**) and dibenzo[*b,d*]furan (**26**), served as effective radical precursors in this transformation, yielding valuable heterocyclic alkylboronic esters. Unfortunately, other heterocycles **1y ∼ 1am** furnished the complex mixtures under these reaction conditions and did not afford the target products (see Figure S5, Supporting Information). Certain compounds (**27**‐**30**), which could potentially suffer from instability during isolation on silica gel,^[^ ^72^ ^]^ were obtained in their ketone form through NaBO_3_•4H_2_O oxidation.

**Figure 3:**
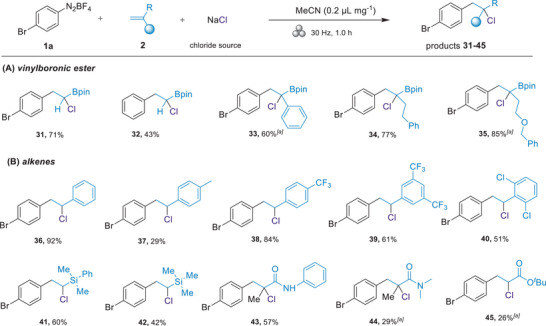
Substrate scope of vinylboronic ester and other alkenes. Reaction conditions: unless otherwise noted, **1a** (0.3 mmol), **2** (0.2 mmol), NaCl (1.5 eq.) and LAG (0.2 µL mg^−1^) were added in a 1.5 mL stainless‐steel milling jar with a stainless‐steel ball (diameter, 5 mm) under nitrogen atmosphere and milled at 30 Hz for 1 h. [a] ^1^H NMR yield with CH_2_Br_2_ as the internal standard.

Next, the scope of unsubstituted and *α*‐substituted vinyl‐boronic esters were assessed (**Figure** **3**A). Surprisingly, the unsubstituted vinyl‐boronic ester, previously reported to yield only 40% under visible‐light‐mediated dual catalysis conditions,^[^ ^39^ ^]^ proved compatible with our reaction conditions, providing secondary *α*‐chloro alkylboronic esters in moderate yield (**31**: 71%; **32**: 43%). However, to the best of our knowledge, previous studies have offered sporadic methods for constructing tertiary *α*‐halo alkylboronic esters, typically relying on expensive metal catalysts or bulk toxic solvents.^[^ ^32^, ^39^ ^]^ With the mechanochemical protocol, we were pleased to find that vinyl‐boronic esters containing an *α*‐aryl group served as excellent substrates for the aryl‐chlorination reaction, yielding the desired products (**33**) with generally high yields. Meanwhile, *α*‐alkyl substituted vinyl‐boronic esters provided the corresponding products in excellent yields (**34**: 77%; **35**: 85%). Unfortunately, *β*‐substituted and branched alkyl substituents with increased steric bulk on the vinyl‐boronic ester were found to be unsuitable as alkene coupling partners (see Figure S6, Supporting Information). This limitation is likely due to steric hindrance slowing down the radical addition step. In addition to vinyl‐boronic esters, various types of olefin acceptors were successfully accommodated (Figure 3B). Styrene (**36**) and its substituted counterparts, including those with electron‐donating (methyl: **37**) and electron‐withdrawing (trifluoromethyl: **38** and **39**) groups, were efficiently transformed into the desired products in good to high yields (up to 92%). Furthermore, the more sterically hindered disubstituted styrene could also yield the target product (**40**) at a respectable 51% yield. Subsequently, we expanded the scope to include silyl vinyl groups, such as dimethyl(phenyl)(vinyl)silane (**41**) and trimethyl(vinyl)silane (**42**), all efficiently forming the corresponding products in 42–60% yields. Moreover, *N*‐phenylmethacrylamide and *N*,*N*‐dimethylmethacrylamide also successfully participated in the reaction (**43**: 57%; **44**: 29%). We also found that *tert*‐butyl acrylate afforded the target product (**45**), albeit with a relatively lower yield of 26%.

**Figure 4:**
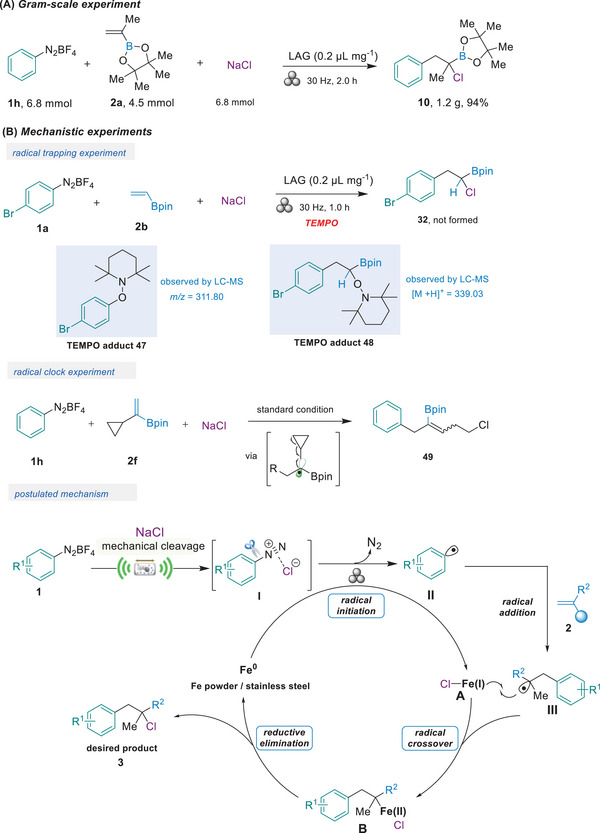
A) Gram‐scale experiment; B) Mechanistic experiments.

To further underscore the practical utility of our method in organic synthesis, we conducted a gram‐scale experiment under ball‐milling conditions (**Figure** **4**A). The reaction between **1** **h** and **2a** at 6.8‐mmol scale was performed in a stainless steel ball‐milling jar (10 mL) using nine stainless steel balls (diameter: 7 mm), resulting in an excellent yield of **10** (94%). This demonstrates the ease with which the reaction can be scaled up and highlights the significant advantages of our mechanochemical strategy, which eliminates the need for bulk toxic solvents and expensive metal catalysts. Subsequently, we carried out a radical trapping experiment to probe the reaction pathway (Figure 4B). The addition of TEMPO completely suppressed the formation of *α*‐chloro alkylboronic esters product **3**, and TEMPO adducts (**46** and **47**) were identified through liquid chromatography mass spectrometry. Moreover, subjecting vinylcyclopropane boronate **2f** to the standard conditions led to alkenyl boronate **48**, which was derived from the cleavage of the cyclopropane ring. These results indicated that aryl radical and *α*‐boryl‐radical were involved in this mechanochemical transformation. Further investigation was conducted to shed light on the mechanism. When ZrO_2_ jars (10 mL) and nine ZrO_2_ balls (7 mm diameter) were used, the reaction did not proceed, and only a trace amount of product **3** was observed by NMR. However, upon adding 20 mol% iron powder—the main element in the mixer mill and stainless‐steel ball—the ZrO_2_ system successfully initiated the reaction, delivering product **3** with a 98% yield (Supporting information 3.7). Based on the experimental results mentioned earlier and the findings in the literature,^[^ ^65^, ^66^, ^73^, ^74^, ^75^, ^76^, ^77^, ^78^, ^79^ ^]^ we propose a mechanistic pathway for the mechanochemical synthesis of *α*‐halo alkylboronic esters (Figure 4B: *postulated mechanism*). First, the NaCl anion undergoes an exchange with the non‐coordinating tetrafluoroborate counterion within the aryldiazonium salt. This exchange results in the formation of an ion pair (**I**). Subsequently, ion pair (**I**) generates aryl radical (**II**) and chlorine radical through intramolecular charge transfer, along with the cleavage of the C–N bond under mechanochemical conditions. Fe(0), present in the mixer mill and stainless‐steel ball, may also react with ion pair (**I**) via a single electron transfer process, generating a Fe(I)–Cl species (**A**).^[^ ^78^ ^]^ This process also liberates nitrogen gas into the surroundings. Following the generation of aryl radical (**II**), it engages in an addition reaction with the olefin acceptor (**2**), leading to the formation of a newly generated *α*‐boryl‐radical (**III**). Subsequently, radical (**III**) is trapped by Fe(**I**)–Cl species (**A**) to form a high‐valent Fe(II) complex (**B**). Finally, intermediate **B** undergoes the reductive elimination to afford the product **3**, along with the regeneration of Fe(0) species to close the catalytic cycle.

## 3 Conclusion

In conclusion, we have successfully developed the external catalyst‐free and solvent‐less mechanochemical protocol for the direct synthesis of *α*‐halo alkylboronic esters. The three‐component coupling reaction is enabled by mechanical force, generating aryl radicals using diazonium tetrafluoroborates as a radical source, alkenes as acceptors, and metal halide as halide sources. The mild conditions, including short reaction times, solvent‐less and external catalyst‐free conditions, along the simplicity of the procedure, make this methodology sustainable and cost‐effective. Furthermore, the reaction exhibits excellent versatility with respect to substituted diazonium salts and olefin acceptors. This innovative mechanochemical protocol offers a sustainable and practical approach for the production of *α*‐halo alkylboronic esters, facilitating the downstream diversifications for complex molecule synthesis.

The authors have cited additional references within the Supporting Information.^[^ ^80^, ^81^, ^82^, ^83^, ^84^, ^85^ ^]^

## Conflict of Interest

The authors declare no conflict of interest.

## Supporting information

Supporting Information

## Data Availability

The data that support the findings of this study are available in the supplementary material of this article.
