# Supplementary material for: Mechanochemical Synthesis of α‐halo Alkylboronic Esters
Source: Adv Sci (Weinh). 2024 Jul 3;11(33):2404071. doi: 10.1002/advs.202404071 (PMC11434113; doi:10.1002/advs.202404071)
Supplement: Supplementary file 1 — Supporting Information [file ADVS-11-2404071-s001.pdf]

ADVANCED SCIENCE
Open Access

Supporting Information

for *Adv. Sci.*, DOI 10.1002/advs.202404071

Mechanochemical Synthesis of α-halo Alkylboronic Esters

Yunyi Zhao, Zekun Yang, Xin Wang, Qinchun Kang, Bobo Wang, Tianle Wu, Hao Lei, Peile Ma, Wenqiang Su, Siyuan Wang, Zhiqiang Wu, Xinsong Huang, Chunying Fan and Xiaofeng Wei**

Supplemental information

Table of Contents

1. General information.. S2

2. General procedures for the synthesis of substrates S4

   2.1 *The synthesis of aryldiazonium tetrafluoroborates*.. S4

   2.2 *The synthesis of vinyl boronic esters* S5

3. Experimental procedures for the synthesis of α-chloroboronic esters S7

   3.1 *General procedure A for the synthesis of α-chloroboronic esters* S7

   3.2 *General procedure B for synthesis of the corresponding ketones*......................... S16

   3.3 *General procedure C for synthesis of products* ***31*** *~* ***45*** S17

   3.4 *Gram-scale experiment for synthesis of α-chloroboronic esters*............................ S23

   3.5 *Radical trapping experiment*.. S23

   3.6 *Radical clock experiment* S24

   3.7 *Ball milling material experiment*.. S25

4. NMR spectroscopic data S27

Supplemental Experimental Procedures

1. General information

Unless otherwise stated, reactions were carried out using dry solvents under argon atmosphere (purity > 99.99%). Commercial reagents were purchased from Adamas, Aladdin, Alfa Aesar, Bidepharm, Leyan and TCI, and used directly without further purification. Conversion was monitored by thin layer chromatography (TLC) using Silicycle 200 mm silica gel GF-254 plates and visualized by UV - light at 254 nm. Flash column chromatographic purification of products was performed over silica gel (200 - 300 mesh). All mechanochemical reactions were carried out using grinding vessels in a Retsch MM400 mill (Figure S1). Both jars (1.5 mL or 10 mL) and balls (5 mm or 7 mm) are made of stainless (Figure S2a), and both jars (10 mL) and balls (7 mm) are made of ZrO_2 (Figure S2b). ^{1}H NMR, ^{13}C NMR and ^{19}F NMR spectra were measured on a Bruker Avance-400 spectrometer at ambient temperature and chemical shifts (δ) are reported in parts per million (ppm). ^{1}H NMR spectra were recorded at 400 MHz in NMR solvents ($CDCl_3$) and referenced internally to corresponding solvent resonance, and ^{13}C NMR spectra were recorded at 101 MHz, Chemical shift was reported in ppm on the δ scale relative to $CDCl_3$ (δ = 7.26 ppm for ^{1}H NMR, δ = 77.16 ppm for ^{13}C NMR). Coupling constants are reported in Hz with multiplicities denoted as s (singlet), d (doublet), t (triplet), dd (doublet-doublet), q (quartet), m (multiplet) and br (broad). Dibromomethane was used as an internal standard to determine NMR yields. High resolution mass Spectra (HRMS) were obtained on a Waters I-Class VION IMS QTof and are reported as m/z (relative intensity).

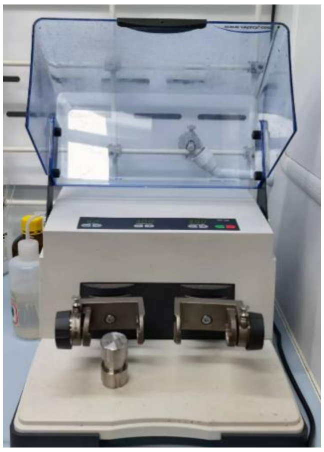

Figure S1. Retsch MM400 used in this study.

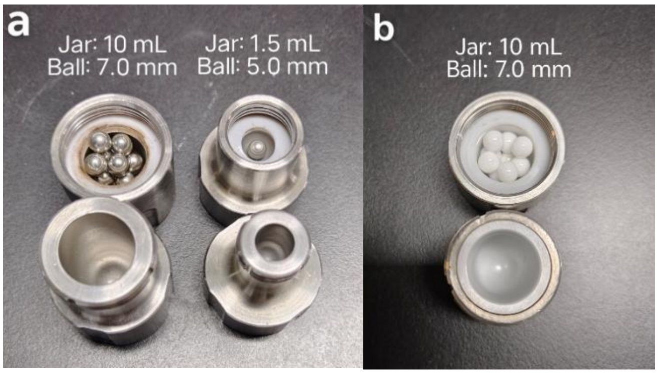

Figure S2. (a) Stainless jar and ball used in this study (1.5 mL and 10 mL). (b) ZrO_2 jar and

balls used in this study (10 mL).

2. General procedures for the synthesis of substrates

2.1 The synthesis of aryldiazonium tetrafluoroborates

Aryldiazonium tetrafluoroborates **1a ~ 1q** were synthesized from the corresponding anilines according to the method A[65]. **1r ~ 1x** were synthesized according to the method B[65].

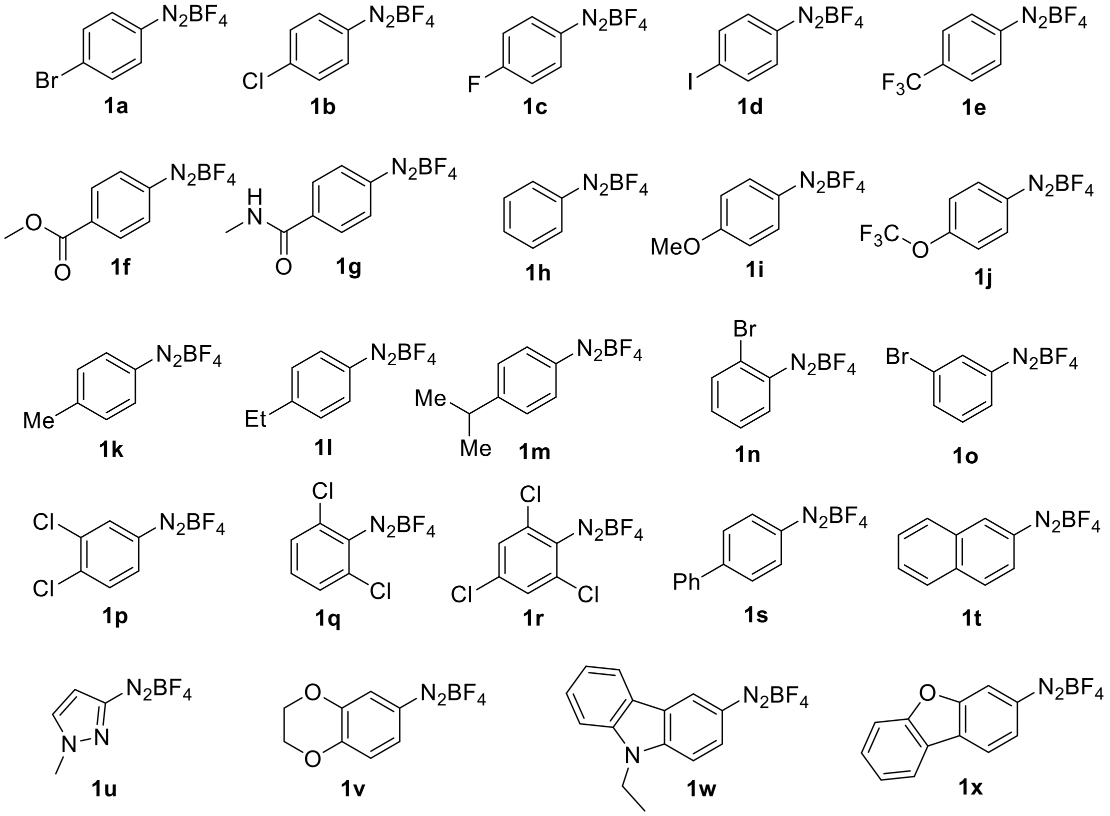

Figure S3. Aryldiazonium tetrafluoroborates used in this study

Method A

In a 100 mL round-bottom flask, the aniline (20.0 mmol) was mixed at 0°C with fluoroboric acid (50 wt.% in H_2O, 7.0 mL) and distilled water (8.0 mL). An aqueous solution of sodium nitrite (1.4 g, 20.2 mmol, 1.0 eq., in 6.0 mL H_2O) was added gradually to the mixture. The mixture was then held at 0°C for 30 min. The thick precipitate was collected by filtration and redissolved in a minimum amount of acetone. Diethyl ether was added until precipitation of the diazonium tetrafluoroborate, which was filtered off and washed several times with diethyl ether and dried under vacuum.

Method B

In a 100 mL round-bottom flask, the aniline (20.0 mmol) was dissolved in a mixture of absolute ethanol (6 mL) and aqueous solution of fluoroboric acid (50 wt.% in H_2O, 5.0 mL). The tertbutyl nitrite (5.4 mL, 44.0 mmol, 2.2 eq.) was added dropwise to the solution at 0°C. The mixture was stirred at room temperature for 1.0 h and diethyl ether (40 mL) was added to precipitate the diazonium tetrafluoroborate. The solid was filtered off and washed with diethyl ether and dried under vacuum.

2.2 *The synthesis of vinyl boronic esters*

Vinyl boronic esters **2a ~ 2c** and **2f** were purchased from commercial suppliers. **2d** and **2e** were synthesized according to the method C[80].

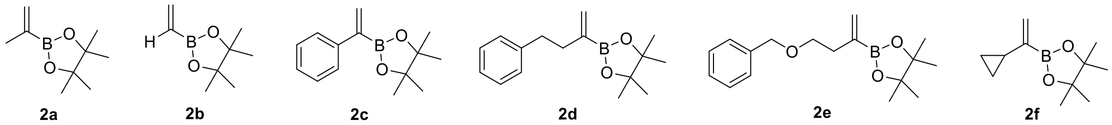

2a **2b** **2c** **2d** **2e** **2f**

Figure S4. Vinyl boronic esters used in this study

Method C

A 48 mL Schlenk tube charged with CuCl (330 mg, 3.3 mmol, 1.1 eq.), LiCl (140 mg, 3.3 mmol, 1.1 eq.) and a magnetic stir bar in glovebox. Then dry DMF (6 mL, 0.5 M) was added in the tube. After being stirred for 1.0 h at room temperature, bis(pinacolato)diboron (840 mg, 3.3 mmol, 1.1 eq.), KOAc (325 mg, 3.3 mmol, 1.1 eq.), and alkyl alkyne (3.0 mmol) were added subsequently. The reaction system was placed in 50°C oil bath and stir for 16 h. After completion of the reaction, the mixture was quenched with water, and extracted with petroleum ether for three times. The combined organic layers were washed with brine for three times, dried over anhydrous Na_2SO_4, and concentrated. The residue was purified by column chromatograph to afford the products.

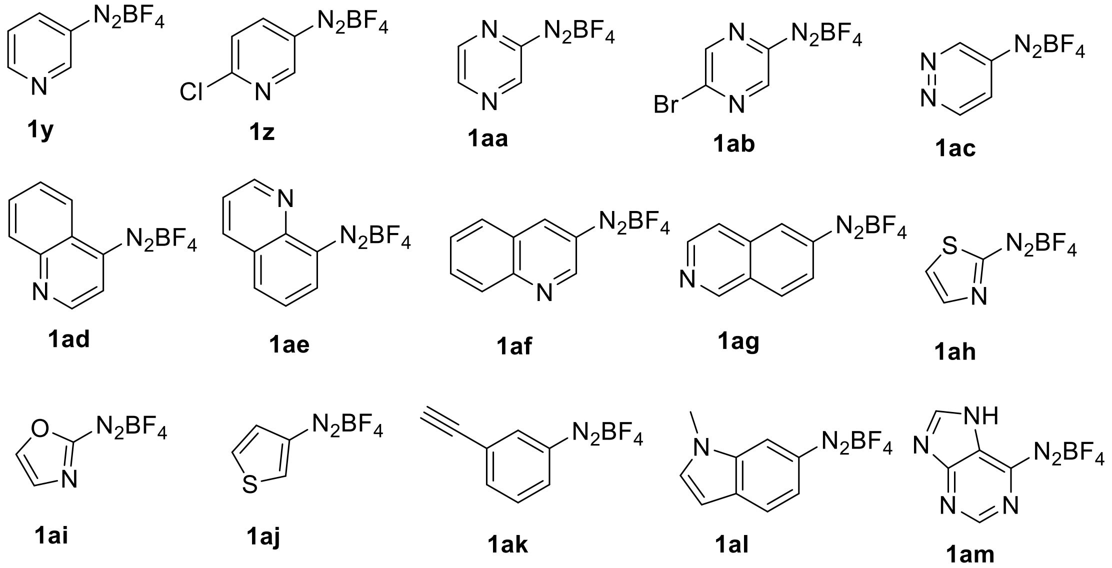

Figure S5. Unsuccessful aryldiazonium tetrafluoroborates in the mechanochemical radical reaction

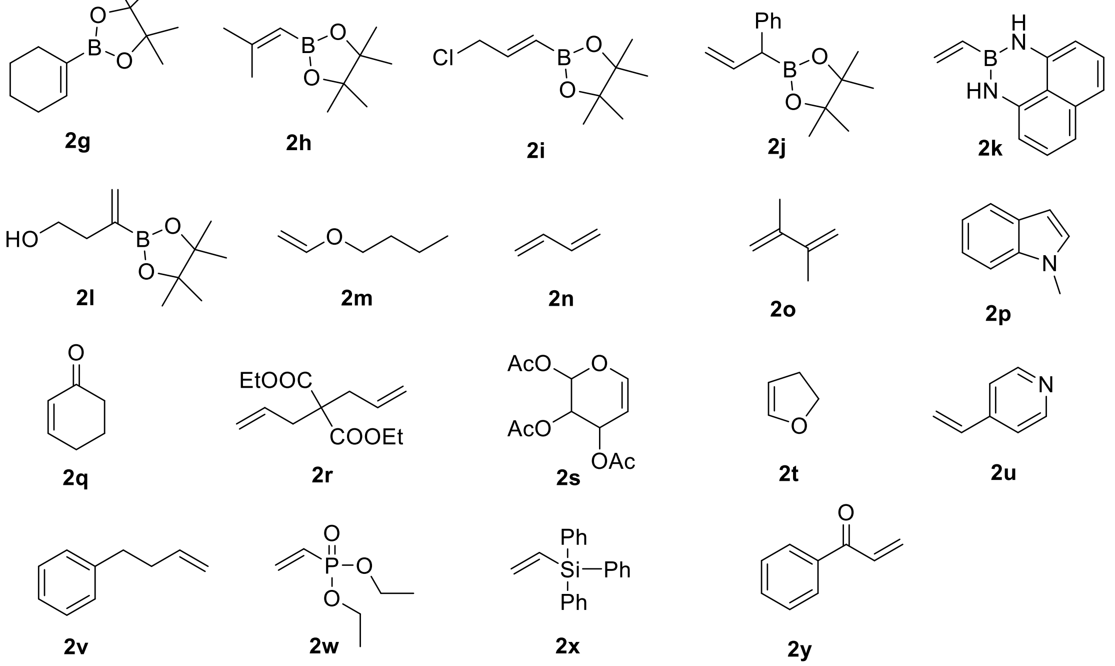

Figure S6. Unsuccessful vinyl boronic esters and other alkenes in the mechanochemical radical reaction

3. Experimental procedures for the synthesis of α-chloroboronic esters

3.1 General procedure A for synthesis of α-chloroboronic esters

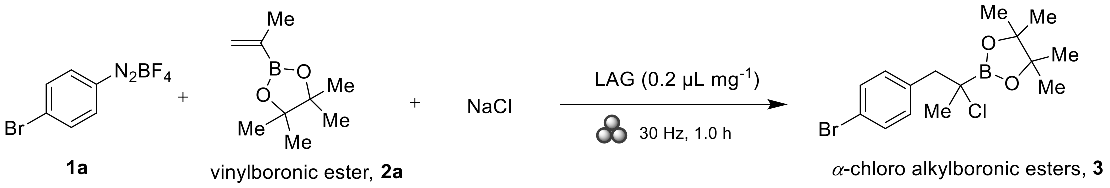

In a nitrogen-filled glove box, NaCl (17.5 mg, 0.3 mmol, 1.5 eq.), **1a** (0.3 mmol, 1.5 eq.), **2a** (33.6 mg, 0.2 mmol, 1.0 eq.), and dry MeCN (0.2 µL mg^{-1}) were placed in a ball milling vessel (stainless, 1.5 mL) loaded with one grinding ball (stainless, diameter: 5.0 mm). Then the vessel was closed and placed in the ball mill (Retsch MM400, 30 Hz). After grinding for 60 min, the mixture was passed through a short celite column eluting with EtOAc to remove inorganic salts. The solvent was evaporated and the residual was purified by column chromatography on silica gel with hexane/ethyl acetate as the eluent to give the desired products **3**.

Note: the carbon attached to boron is not observed in the ^{13}C NMR due to quadrupolar relaxation of boron!

2-(1-(4-bromophenyl)-2-chloropropan-2-yl)-4,4,5,5-tetramethyl-1,3,2-dioxaborolane (3)

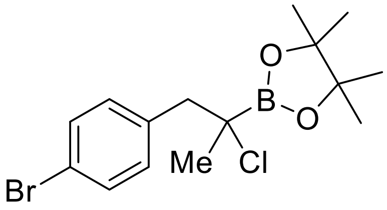

Prepared according to general procedure A using 4-bromobenzenediazonium tetrafluoroborate (81.3 mg, 0.30 mmol). **3** (71.5 mg, 0.199 mmol, 99%) was purified by flash column chromatography (eluent: hexane/ethyl acetate = 10:1) as colourless oil.

^{1}H NMR (400 MHz, CDCl$_3$) δ 7.39 (d, *J* = 8.4 Hz, 2H), 7.17 (d, *J* = 8.5 Hz, 2H), 3.16 (d, *J* = 13.5 Hz, 1H), 3.02 (d, *J* = 13.5 Hz, 1H), 1.48 (s, 3H), 1.25 (s, 6H), 1.25 (s, 6H).

^{13}C NMR (101 MHz, CDCl$_3$) δ 136.33, 132.43, 131.14, 120.94, 84.74, 46.76, 26.31, 24.66 (d, *J* = 4.2 Hz).

^{11}B NMR (128 MHz, CDCl$_3$) δ 30.75.

Spectral data match those previously reported[39].

2-(2-chloro-1-(4-chlorophenyl)propan-2-yl)-4,4,5,5-tetramethyl-1,3,2-dioxaborolane (4)

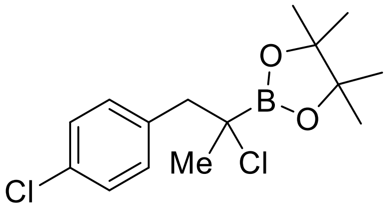

Prepared according to general procedure A using 4-chlorobenzenediazonium tetrafluoroborate (67.9 mg, 0.30 mmol). **4** (62.7 mg, 0.199 mmol, 99%) was purified by flash column chromatography (eluent: hexane/ethyl acetate = 10:1) as colourless oil.

^{1}H NMR (400 MHz, CDCl$_3$) δ 7.23 (s, 4H), 3.18 (d, J = 13.6 Hz, 1H), 3.03 (d, J = 13.5 Hz, 1H), 1.49 (s, 3H), 1.26 (s, 6H), 1.24 (s, 6H).

^{13}C NMR (101 MHz, CDCl$_3$) δ 135.83, 132.80, 132.04, 128.18, 84.73, 46.73, 26.32, 24.65 (d, J = 3.9 Hz).

^{11}B NMR (128 MHz, CDCl$_3$) δ 30.36.

Spectral data match those previously reported[39].

2-(2-chloro-1-(4-fluorophenyl)propan-2-yl)-4,4,5,5-tetramethyl-1,3,2-dioxaborolane (5)

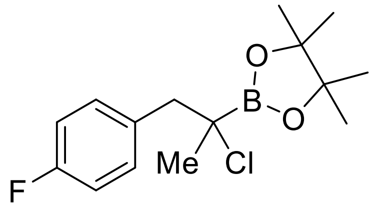

Prepared according to general procedure A using 4-fluorobenzenediazonium tetrafluoroborate (63.0 mg, 0.30 mmol). **5** (47.8 mg, 0.160 mmol, 80%) was purified by flash column chromatography (eluent: hexane/ethyl acetate = 10:1) as colourless oil.

^{1}H NMR (400 MHz, CDCl$_3$) δ 7.31 – 7.23 (m, 2H), 6.95 (t, J = 8.6 Hz, 2H), 3.19 (d, J = 13.7 Hz, 1H), 3.03 (d, J = 13.7 Hz, 1H), 1.49 (s, 3H), 1.26 (d, J = 5.8 Hz, 6H), 1.24 (s, 6H).

^{13}C NMR (101 MHz, CDCl$_3$) δ 163.23, 160.80, 133.08 (d, J = 2.5 Hz), 132.15 (d, J = 8.1 Hz), 114.82 (d, J = 21.1 Hz), 84.68, 46.62, 26.32, 24.64.

^{19}F NMR (376 MHz, CDCl$_3$) δ -116.19.

^{11}B NMR (128 MHz, CDCl$_3$) δ 30.51.

Spectral data match those previously reported[39].

2-(2-chloro-1-(4-(trifluoromethyl)phenyl)propan-2-yl)-4,4,5,5-tetramethyl-1,3,2-dioxaborolane (7)

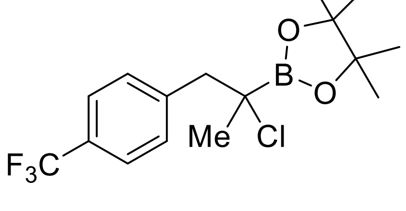

Prepared according to general procedure A using 4-trifluoromethylphenyldiazonium

tetrafluoroborate (78.0 mg, 0.30 mmol). **7** (50.1 mg, 0.144 mmol, 72%) was purified by flash column chromatography (eluent: hexane/ethyl acetate = 10:1) as colourless oil.

^{1}H NMR (400 MHz, $CDCl_3$) δ 7.53 (d, *J* = 8.1 Hz, 2H), 7.42 (d, *J* = 8.1 Hz, 2H), 3.25 (d, *J* = 13.5 Hz, 1H), 3.14 (d, *J* = 13.5 Hz, 1H), 1.50 (s, 3H), 1.26 (s, 6H), 1.25 (s, 6H).

^{13}C NMR (101 MHz, $CDCl_3$) δ 141.40, 131.02, 124.93 (q, *J* = 3.5 Hz), 84.82, 47.04, 26.41, 24.63 (d, *J* = 5.2 Hz).

^{19}F NMR (376 MHz, $CDCl_3$) δ -62.32.

^{11}B NMR (128 MHz, $CDCl_3$) δ 30.32.

Spectral data match those previously reported[39].

Methyl 4-(2-chloro-2-(4,4,5,5-tetramethyl-1,3,2-dioxaborolan-2-yl) propyl) benzoate (8)

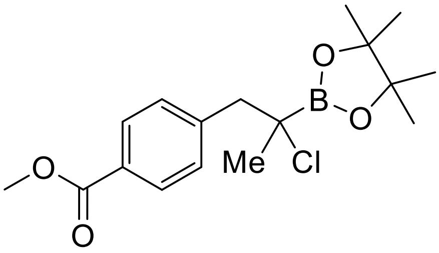

Prepared according to general procedure A using 4-methoxycarbonylphenyldiazonium tetrafluoroborate (75.0 mg, 0.30 mmol). **8** (52.2 mg, 0.154 mmol, 77%) was purified by flash column chromatography (eluent: hexane/ethyl acetate = 10:1) as colourless oil.

^{1}H NMR (400 MHz, $CDCl_3$) δ 7.93 (d, *J* = 8.0 Hz, 2H), 7.37 (d, *J* = 8.1 Hz, 2H), 3.88 (s, 3H), 3.27 (d, *J* = 13.3 Hz, 1H), 3.11 (d, *J* = 13.3 Hz, 1H), 1.49 (s, 3H), 1.25 (s, 6H), 1.24 (s, 6H).

^{13}C NMR (101 MHz, $CDCl_3$) δ 167.16, 142.79, 131.07, 130.71, 129.35, 128.78 (d, *J* = 6.5 Hz), 84.77, 52.15, 47.39, 26.44, 24.64 (d, *J* = 2.8 Hz).

^{11}B NMR (128 MHz, $CDCl_3$) δ 31.12.

HRMS (ESI) m/z calcd for $C_{17}H_{24}BO_4$ $[M-Cl]^+$: 303.17676, found 303.17609.

4-(2-chloro-2-(4,4,5,5-tetramethyl-1,3,2-dioxaborolan-2-yl)propyl)-*N*-methylbenzamide (9)

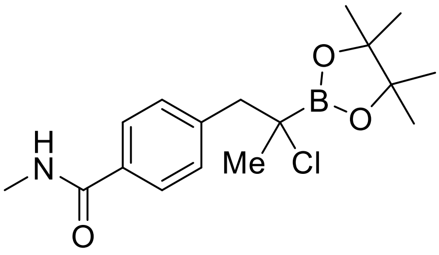

Prepared according to general procedure A using 4-(methylcarbamoyl)benzenediazonium tetrafluoroborate (74.7 mg, 0.30 mmol). **9** (36.0 mg, 0.107 mmol, 53%) was purified by flash column chromatography (eluent: hexane/ethyl acetate = 10:1) as colourless oil.

^{1}H NMR (400 MHz, $CDCl_3$) δ 7.63 – 7.58 (m, 2H), 7.30 (d, *J* = 8.1 Hz, 2H), 6.10 (s, 1H), 3.19 (d, *J* = 13.4 Hz, 1H), 3.05 (d, *J* = 13.4 Hz, 1H), 2.94 (d, *J* = 4.7 Hz, 3H), 1.43 (s, 3H), 1.19 (s, 6H), 1.18 (s, 6H).

^{13}C NMR (101 MHz, CDCl$_3$) δ 168.15, 141.00, 137.60, 133.02, 130.80, 128.82, 128.58, 128.31, 126.83, 126.52, 84.68, 50.87, 47.12, 26.92, 26.86, 26.34, 24.59, 24.56.

^{11}B NMR (128 MHz, CDCl$_3$) δ 30.82.

HRMS (ESI) m/z calcd for $C_{17}H_{26}BClNO_3$ [M+H]$^+$: 338.16888, found 338.17173.

2-(2-chloro-1-phenylpropan-2-yl)-4,4,5,5-tetramethyl-1,3,2-dioxaborolane (10)

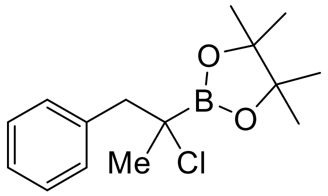

Prepared according to general procedure A using benzenediazonium tetrafluoroborate (57.6 mg, 0.30 mmol). **10** (52.7 mg, 0.188 mmol, 94%) was purified by flash column chromatography (eluent: hexane/ethyl acetate = 10:1) as colourless oil.

^{1}H NMR (400 MHz, CDCl$_3$) δ 7.32 – 7.22 (m, 5H), 3.28 (d, *J* = 13.5 Hz, 1H), 3.07 (d, *J* = 13.5 Hz, 1H), 1.51 (s, 3H), 1.27 (s, 6H), 1.25 (s, 6H).

^{13}C NMR (101 MHz, CDCl$_3$) δ 137.41, 130.67, 128.07, 126.86, 84.63, 47.58, 26.35, 24.65.

^{11}B NMR (128 MHz, CDCl$_3$) δ 30.83.

Spectral data match those previously reported[39].

2-(2-chloro-1-(4-methoxyphenyl)propan-2-yl)-4,4,5,5-tetramethyl-1,3,2-dioxaborolane (11)

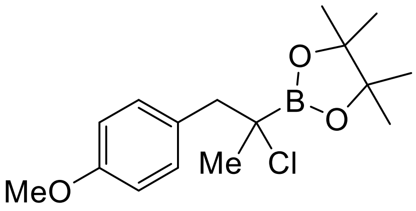

Prepared according to general procedure A using 4-methoxybenzenediazonium tetrafluoroborate (66.6 mg, 0.30 mmol). **11** (53.7 mg, 0.173 mmol, 87%) was purified by flash column chromatography (eluent: hexane/ethyl acetate = 10:1) as brown oil.

^{1}H NMR (400 MHz, CDCl$_3$) δ 7.21 (d, *J* = 8.2 Hz, 2H), 6.80 (d, *J* = 8.3 Hz, 2H), 3.77 (s, 3H), 3.20 (d, *J* = 13.5 Hz, 1H), 2.99 (d, *J* = 13.5 Hz, 1H), 1.49 (s, 3H), 1.26 (s, 6H), 1.24 (s, 6H).

^{13}C NMR (101 MHz, CDCl$_3$) δ 158.57, 131.67, 129.53, 113.45, 84.58, 55.29, 46.70, 26.25, 24.66.

^{11}B NMR (128 MHz, CDCl$_3$) δ 30.43.

Spectral data match those previously reported[39].

2-(2-chloro-1-(4-(trifluoromethoxy)phenyl)propan-2-yl)-4,4,5,5-tetramethyl-1,3,2-dioxaborolane (12)

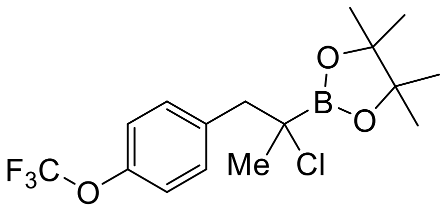

Prepared according to general procedure A using 4-(trifluoromethoxy)benzenediazonium tetrafluoroborate (82.8 mg, 0.30 mmol). **12** (35.4 mg, 0.097 mmol, 49%) was purified by flash column chromatography (eluent: hexane/ethyl acetate = 10:1) as yellow oil.

^{1}H NMR (400 MHz, CDCl$_3$) δ 7.33 (d, *J* = 8.5 Hz, 2H), 7.11 (d, *J* = 8.2 Hz, 2H), 3.21 (d, *J* = 13.6 Hz, 1H), 3.08 (d, *J* = 13.7 Hz, 1H), 1.51 (s, 3H), 1.25 (s, 6H), 1.24 (s, 6H).

^{13}C NMR (101 MHz, CDCl$_3$) δ 148.21, 136.03, 131.93, 120.42, 84.67, 46.61, 26.37, 24.53 (d, *J* = 4.4 Hz).

^{19}F NMR (376 MHz, CDCl$_3$) δ -57.90.

^{11}B NMR (128 MHz, CDCl$_3$) δ 29.96.

Spectral data match those previously reported[39].

2-(1-(2-bromophenyl)-2-chloropropan-2-yl)-4,4,5,5-tetramethyl-1,3,2-dioxaborolane (16)

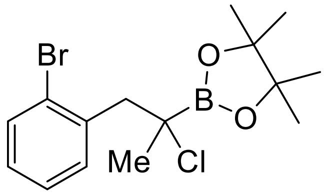

Prepared according to general procedure A using 2-bromobenzenediazonium tetrafluoroborate (81.3 mg, 0.30 mmol). **16** (68.3 mg, 0.190 mmol, 95%) was purified by flash column chromatography (eluent: hexane/ethyl acetate = 10:1) as pale-yellow solids.

^{1}H NMR (400 MHz, CDCl$_3$) δ 7.54 (d, *J* = 7.9 Hz, 2H), 7.21 (d, *J* = 7.6 Hz, 1H), 7.08 (t, *J* = 7.7 Hz, 1H), 3.41 (s, 1H), 3.40 (s, 1H), 1.56 (s, 3H), 1.30 (s, 6H), 1.28 (s, 6H).

^{13}C NMR (101 MHz, CDCl$_3$) δ 137.14, 132.96, 131.97, 128.33, 126.98, 126.08, 84.63, 45.14, 25.81, 24.57.

^{11}B NMR (128 MHz, CDCl$_3$) δ 31.15.

Spectral data match those previously reported[39].

2-(1-(3-bromophenyl)-2-chloropropan-2-yl)-4,4,5,5-tetramethyl-1,3,2-dioxaborolane (17)

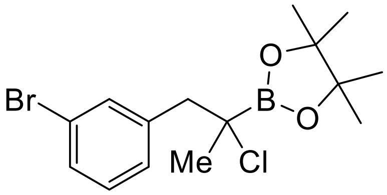

Prepared according to general procedure A using 3-bromobenzenediazonium tetrafluoroborate (81.3 mg, 0.30 mmol). **17** (71.5 mg, 0.199 mmol, 99%) was purified by flash column chromatography (eluent: hexane/ethyl acetate = 10:1) as pale-yellow solids.

^{1}H NMR (400 MHz, CDCl$_3$) δ 7.50 (s, 1H), 7.35 (d, *J* = 7.9 Hz, 1H), 7.21 (d, *J* = 7.7 Hz, 1H),

7.13 (t, *J* = 7.8 Hz, 1H), 3.22 (d, *J* = 13.4 Hz, 1H), 3.01 (d, *J* = 13.4 Hz, 1H), 1.51 (s, 3H), 1.27 (s, 6H), 1.25 (s, 6H).

^{13}C NMR (101 MHz, $CDCl_3$) δ 139.82, 133.44, 129.96, 129.66, 129.39, 122.18, 84.81, 47.23, 26.49, 24.69 (d, *J* = 3.9 Hz).

^{11}B NMR (128 MHz, $CDCl_3$) δ 30.38.

Spectral data match those previously reported[39].

2-(2-chloro-1-(3,4-dichlorophenyl)propan-2-yl)-4,4,5,5-tetramethyl-1,3,2-dioxaborolane (18)

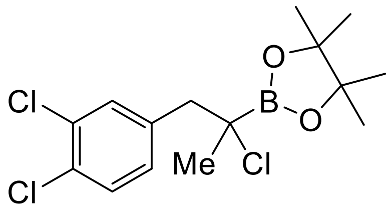

Prepared according to general procedure A using 3,4-dichlorobenzenediazonium tetrafluoroborate (78.2 mg, 0.30 mmol). **18** (67.8 mg, 0.194 mmol, 97%) was purified by flash column chromatography (eluent: hexane/ethyl acetate = 10:1) as pale-yellow oil.

^{1}H NMR (400 MHz, $CDCl_3$) δ 7.44 (d, *J* = 2.1 Hz, 1H), 7.33 (d, *J* = 8.2 Hz, 1H), 7.12 (dd, *J* = 8.2, 2.0 Hz, 1H), 3.16 (d, *J* = 13.5 Hz, 1H), 3.01 (d, *J* = 13.5 Hz, 1H), 1.50 (s, 3H), 1.26 (s, 6H), 1.25 (s, 6H).

^{13}C NMR (101 MHz, $CDCl_3$) δ 137.71, 132.42, 131.99, 130.98, 130.19, 129.98, 84.89, 46.57, 26.48, 24.68 (d, *J* = 5.9 Hz).

^{11}B NMR (128 MHz, $CDCl_3$) δ 30.71.

Spectral data match those previously reported[39].

2-(2-chloro-1-(2,6-dichlorophenyl)propan-2-yl)-4,4,5,5-tetramethyl-1,3,2-dioxaborolane (19)

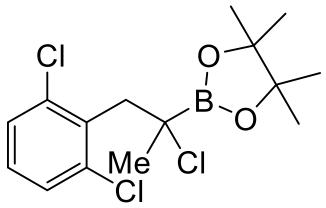

Prepared according to general procedure A using 2,6-dichlorobenzenediazonium tetrafluoroborate (78.2 mg, 0.30 mmol). **19** (63.8 mg, 0.183 mmol, 91%) was purified by flash column chromatography (eluent: hexane/ethyl acetate = 10:1) as yellow oil.

^{1}H NMR (400 MHz, $CDCl_3$) δ 7.29 (d, *J* = 8.0 Hz, 2H), 7.10 (t, *J* = 8.1 Hz, 1H), 3.71 (d, *J* = 14.5 Hz, 1H), 3.58 (d, *J* = 14.6 Hz, 1H), 1.58 (s, 3H), 1.28 (s, 12H).

^{13}C NMR (101 MHz, $CDCl_3$) δ 136.98, 134.39, 128.43 (d, *J* = 9.8 Hz), 84.60, 40.51, 27.30, 24.76 (d, *J* = 5.1 Hz).

^{11}B NMR (128 MHz, $CDCl_3$) δ 30.39.

HRMS (ESI) m/z calcd for $C_{15}H_{21}BCl_3O_2$ $[M+H]^+$: 349.06947, found 349.07147.

2-(2-chloro-1-(2,4,6-trichlorophenyl)propan-2-yl)-4,4,5,5-tetramethyl-

1,3,2-dioxaborolane (20)

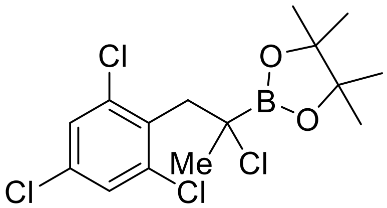

Prepared according to general procedure A using 2,4,6-trichlorobenzenediazonium tetrafluoroborate (88.6 mg, 0.30 mmol). **20** (52.2 mg, 0.136 mmol, 68%) was purified by flash column chromatography (eluent: hexane/ethyl acetate = 10:1) as colourless oil.

^{1}H NMR (400 MHz, $CDCl_3$) δ 7.59 (s, 2H), 3.92 (d, *J* = 14.6 Hz, 1H), 3.76 (d, *J* = 14.6 Hz, 1H), 1.84 (s, 3H), 1.55 (s, 12H).

^{13}C NMR (101 MHz, $CDCl_3$) δ 138.06, 135.50, 133.76 (d, *J* = 13.2 Hz), 129.46, 128.95, 85.31, 40.66, 28.07, 25.46 (d, *J* = 4.8 Hz), 20.29.

^{11}B NMR (128 MHz, $CDCl_3$) δ 29.30.

HRMS (ESI) m/z calcd for $C_{15}H_{19}BCl_4O_2$ $[M]^+$: 382.02322, found 382.02417.

2-(1-([1,1'-biphenyl]-4-yl)-2-chloropropan-2-yl)-4,4,5,5-tetramethyl-1,3,2-dioxaborolane (21)

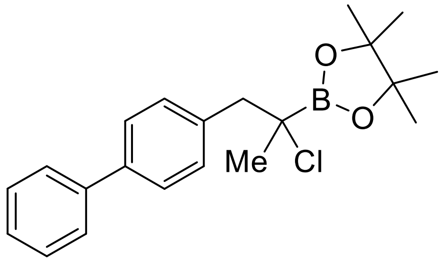

Prepared according to general procedure A using 4-biphenyldiazonium tetrafluoroborate (80.4 mg, 0.30 mmol). **21** (70.6 mg, 0.198 mmol, 99%) was purified by flash column chromatography (eluent: hexane/ethyl acetate = 10:1) as brown solids.

^{1}H NMR (400 MHz, $CDCl_3$) δ 7.59 (d, *J* = 7.0 Hz, 2H), 7.52 (d, *J* = 8.3 Hz, 2H), 7.44 (d, *J* = 4.1 Hz, 2H), 7.42 (d, *J* = 2.3 Hz, 1H), 7.40 (d, *J* = 8.1 Hz, 2H), 3.33 (d, *J* = 13.4 Hz, 1H), 3.13 (d, *J* = 13.4 Hz, 1H), 1.58 (s, 3H), 1.30 (s, 6H), 1.28 (s, 6H).

^{13}C NMR (101 MHz, $CDCl_3$) δ 141.05, 140.10, 139.75, 136.55, 131.11, 129.02, 129.00, 128.86, 128.51, 127.71, 127.37, 127.28, 127.13, 127.09, 126.81, 84.70, 47.23, 26.46, 24.69 (d, *J* = 3.6 Hz).

^{11}B NMR (128 MHz, $CDCl_3$) δ 30.25.

HRMS (ESI) m/z calcd for $C_{21}H_{26}BO_2$ $[M-Cl]^+$: 321.20259, found 321.20377.

2-(2-chloro-1-(naphthalen-2-yl)propan-2-yl)-4,4,5,5-tetramethyl-1,3,2-dioxaborolane (22)

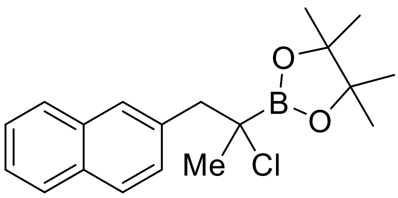

Prepared according to general procedure A using 2-naphthalenediazonium tetrafluoroborate (70.2 mg, 0.30 mmol). **22** (60.0 mg, 0.181 mmol, 91%) was purified by flash column chromatography (eluent: hexane/ethyl acetate = 10:1) as brown solids.

^{1}H NMR (400 MHz, CDCl$_3$) δ 7.72 – 7.65 (m, 4H), 7.38 – 7.33 (m, 3H), 3.38 (d, *J* = 13.3 Hz, 1H), 3.14 (d, *J* = 13.3 Hz, 1H), 1.47 (s, 3H), 1.19 (s, 6H), 1.16 (s, 6H).

^{13}C NMR (101 MHz, CDCl$_3$) δ 135.09, 133.30, 132.42, 129.14, 129.01, 127.65, 127.51, 125.96, 125.59, 84.61, 47.72, 26.42, 24.68, 24.60.

^{11}B NMR (128 MHz, CDCl$_3$) δ 30.63.

HRMS (ESI) m/z calcd for $C_{19}H_{28}BClNO_2$ $[M+NH_4]^+$: 348.18961, found 348.18834.

3-(2-chloro-2-(4,4,5,5-tetramethyl-1,3,2-dioxaborolan-2-yl)propyl)-1-methyl-1*H*-pyrazole (23)

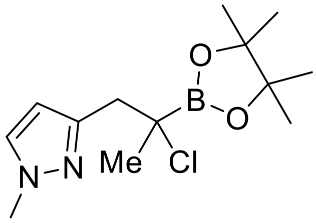

Prepared according to general procedure A using 1-methyl-1*H*-pyrazole-3-diazonium tetrafluoroboranuide (58.8 mg, 0.30 mmol). **23** (43.4 mg, 0.153 mmol, 76%) was purified by flash column chromatography (eluent: hexane/ethyl acetate = 10:1) as yellow oil.

^{1}H NMR (400 MHz, CDCl$_3$) δ 7.21 (d, *J* = 2.2 Hz, 1H), 6.14 (d, *J* = 2.1 Hz, 1H), 3.81 (s, 3H), 3.24 (d, *J* = 14.3 Hz, 1H), 3.09 (d, *J* = 14.3 Hz, 1H), 1.54 (s, 3H), 1.26 (s, 6H), 1.25 (s, 6H).

^{13}C NMR (101 MHz, CDCl$_3$) δ 148.81, 130.45, 106.09, 84.48, 40.41, 38.74, 26.50, 24.67, 24.63, 24.56.

^{11}B NMR (128 MHz, CDCl$_3$) δ 30.34.

HRMS (ESI) m/z calcd for $C_{13}H_{23}BClN_2O_2$ $[M+H]^+$: 285.15356, found 285.15494.

2-(2-chloro-1-(2,3-dihydrobenzo[b][1,4]dioxin-6-yl)propan-2-yl)-4,4,5,5-tetramethyl-1,3,2-dioxaborolane (24)

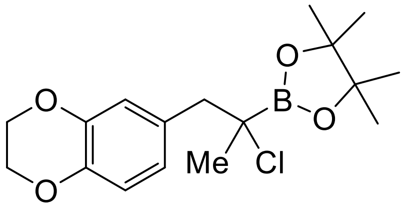

Prepared according to general procedure A using 2,3-dihydro-1,4-benzodioxine-6-diazonium tetrafluoroborate (75.0 mg, 0.30 mmol). **24** (67.1 mg, 0.198 mmol, 99%) was purified by flash column chromatography (eluent: hexane/ethyl acetate = 10:1) as brown solids.

^{1}H NMR (400 MHz, CDCl$_3$) δ 6.82 (s, 1H), 6.74 (d, *J* = 1.2 Hz, 2H), 4.21 (s, 4H), 3.16 (d, *J* = 13.5 Hz, 1H), 2.92 (d, *J* = 13.4 Hz, 1H), 1.49 (s, 3H), 1.27 (s, 6H), 1.25 (s, 6H).

^{13}C NMR (101 MHz, CDCl$_3$) δ 143.07, 142.51, 130.70, 123.63, 119.33, 116.73, 84.63, 64.44, 46.93, 26.29, 24.67.

^{11}B NMR (128 MHz, CDCl$_3$) δ 29.62.

HRMS (ESI) m/z calcd for $C_{17}H_{24}BNaClO_4$ [M+Na]$^+$: 361.13484, found 361.13658.

3-(2-chloro-2-(4,4,5,5-tetramethyl-1,3,2-dioxaborolan-2-yl)propyl)-9-ethyl-9*H*-carbazole (25)

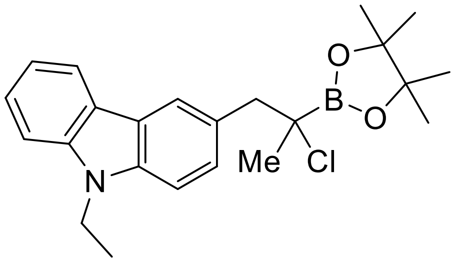

Prepared according to general procedure A using 9-ethyl-9*H*-carbazole-3-diazonium tetrafluoroborate (92.7 mg, 0.30 mmol). **25** (63.1 mg, 0.159 mmol, 79%) was purified by flash column chromatography (eluent: hexane/ethyl acetate = 10:1) as brown solids.

^{1}H NMR (400 MHz, CDCl$_3$) δ 8.15 – 8.11 (m, 1H), 8.08 (s, 1H), 7.38 (d, *J* = 31.0 Hz, 5H), 4.40 – 4.34 (m, 2H), 3.56 (d, *J* = 13.4 Hz, 1H), 3.26 (d, *J* = 13.4 Hz, 1H), 1.61 (s, 3H), 1.44 (d, *J* = 5.8 Hz, 3H), 1.32 (s, 6H), 1.28 (s, 6H).

^{13}C NMR (101 MHz, CDCl$_3$) δ 140.30, 140.04, 139.14, 128.54, 127.93, 125.69 (d, *J* = 8.4 Hz), 123.04, 122.97, 122.87, 122.29, 120.46 (d, *J* = 15.4 Hz), 118.83 (d, *J* = 5.5 Hz), 108.57 (d, *J* = 3.7 Hz), 107.99, 84.62, 47.90, 37.66, 26.43, 24.77 (d, *J* = 7.8 Hz), 13.93.

^{11}B NMR (128 MHz, CDCl$_3$) δ 31.67.

HRMS (ESI) m/z calcd for $C_{23}H_{30}BClNO_2$ [M+H]$^+$: 398.20526, found 398.20644.

2-(2-chloro-1-(dibenzo[b,d]furan-3-yl)propan-2-yl)-4,4,5,5-tetramethyl-1,3,2-dioxaborolane (26)

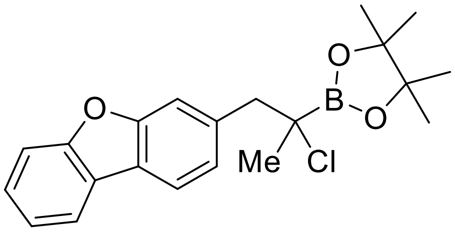

Prepared according to general procedure A using 3-dibenzofurandiazonium tetrafluoroborate (84.6 mg, 0.30 mmol). **26** (70.8 mg, 0.191 mmol, 96%) was purified by flash column chromatography (eluent: hexane/ethyl acetate = 10:1) as yellow solids.

^{1}H NMR (400 MHz, CDCl$_3$) δ 7.92 (dd, *J* = 7.7, 1.3 Hz, 1H), 7.84 (s, 1H), 7.60 – 7.53 (m, 3H), 7.46 – 7.43 (m, 1H), 7.33 (s, 1H), 3.46 (d, *J* = 13.4 Hz, 1H), 3.24 (d, *J* = 13.4 Hz, 1H), 1.58 (s, 3H), 1.31 (s, 6H), 1.28 (s, 6H).

^{13}C NMR (101 MHz, CDCl$_3$) δ 156.39 (d, *J* = 16.6 Hz), 137.09, 127.25, 127.03, 125.53, 124.26, 122.98, 122.78, 120.70 (d, *J* = 12.9 Hz), 120.04, 113.66, 111.76, 84.76, 47.93, 26.45, 24.72,

24.71.
^{11}B NMR (128 MHz, CDCl$_3$) δ 31.82.
HRMS (ESI) m/z calcd for $C_{21}H_{24}BNaClO_3$ [M+Na]$^+$: 393.13992, found 393.14087.

3.2 General procedure B for synthesis of the corresponding ketones

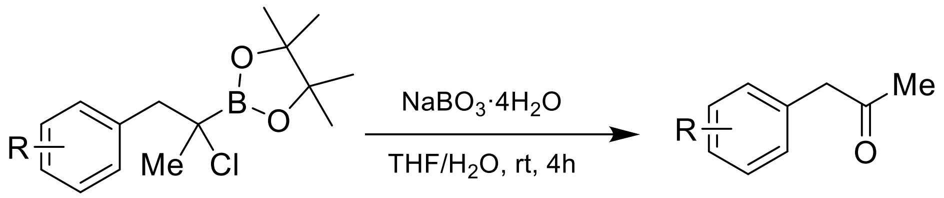

The isolation of **6**, **13**, **14**, **15** was found to be highly cumbersome due to their instability on silica gel, hence the resulting products were in situ oxidized into the corresponding ketones using $NaBO_3$•$4H_2O$ in aqueous THF (general procedure B). A solution of crude product was dissolved in THF (1.5 mL) and deionized water (0.5 mL). Sodium perborate tetrahydrate (135.0 mg, 7.0 eq.) was then added in one portion and the reaction stirred for 4 h at room temperature. The reaction was then diluted with diethyl ether (5 mL) and water (5 mL) and the organic layer extracted. The aqueous layer was extracted with additional diethyl ether (5 mL) and the combined organic extracts were dried over anhydrous Na_2SO_4 and concentrated via rotary evaporation. The resulting crude oil was purified by passing the crude material through a short pad of SiO_2, eluting with hexanes/EtOAc (5:1) to yield the desired compound **27** ~ **30**.

1-(4-iodophenyl)propan-2-one (27)

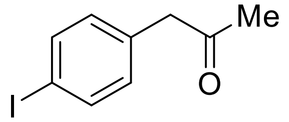

Prepared according to general procedure B using compound **6**. **27** (25.3 mg, 0.097 mmol, 51%) was purified by flash column chromatography (eluent: hexane/ethyl acetate = 5:1) as colourless oil.
^{1}H NMR (400 MHz, CDCl$_3$) δ 7.59 (d, *J* = 8.3 Hz, 2H), 6.87 (d, *J* = 8.3 Hz, 2H), 3.57 (s, 2H), 2.09 (s, 3H).
^{13}C NMR (101 MHz, CDCl$_3$) δ 205.52, 137.82, 133.76, 131.44, 92.61, 50.28, 29.46.
Spectral data match those previously reported[81].

1-(p-tolyl)propan-2-one (28)

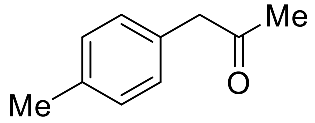

Prepared according to general procedure B using compound **13**. **28** (6.7 mg, 0.045 mmol, 50%) was purified by flash column chromatography (eluent: hexane/ethyl acetate = 5:1) as colourless oil.
^{1}H NMR (400 MHz, CDCl$_3$) δ 7.08 (d, *J* = 7.9 Hz, 2H), 7.02 (d, *J* = 8.0 Hz, 2H), 3.58 (s, 2H),

2.27 (s, 3H), 2.07 (s, 3H).
^{13}C NMR (101 MHz, $CDCl_3$) δ 206.89, 136.82, 131.27, 129.57, 129.35, 50.76, 29.28, 21.17.
Spectral data match those previously reported[82].

1-(4-ethylphenyl)propan-2-one (29)

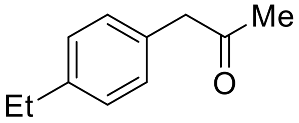

Prepared according to general procedure B using compound **14**. **29** (13.0 mg, 0.080 mmol, 56%) was purified by flash column chromatography (eluent: hexane/ethyl acetate = 5:1) as colourless oil.
^{1}H NMR (400 MHz, $CDCl_3$) δ 7.10 (d, J = 8.0 Hz, 2H), 7.05 (d, J = 8.0 Hz, 2H), 3.59 (s, 2H), 2.57 (q, J = 7.8 Hz, 2H), 2.08 (s, 3H), 1.16 (t, J = 7.6 Hz, 3H).
^{13}C NMR (101 MHz, $CDCl_3$) δ 129.42, 128.38, 50.78, 29.32, 28.56, 15.61.
Spectral data match those previously reported[83].

1-(4-isopropylphenyl)propan-2-one (30)

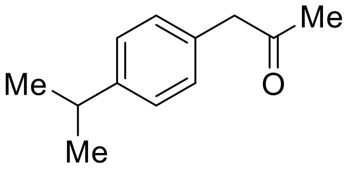

Prepared according to general procedure B using compound **15**. **30** (8.5 mg, 0.048 mmol, 37%) was purified by flash column chromatography (eluent: hexane/ethyl acetate = 5:1) as pale-yellow oil.
^{1}H NMR (400 MHz, $CDCl_3$) δ 7.12 (d, J = 8.0 Hz, 2H), 7.06 (d, J = 8.1 Hz, 2H), 3.59 (s, 2H), 2.85 – 2.78 (m, 1H), 2.08 (s, 3H), 1.18 (s, 3H), 1.16 (s, 3H).
^{13}C NMR (101 MHz, $CDCl_3$) δ 206.79, 147.69, 131.52, 129.31, 126.85, 50.67, 33.76, 29.28, 23.99.
Spectral data match those previously reported[84].

3.3 General procedure C for synthesis of products 31 ~ 45

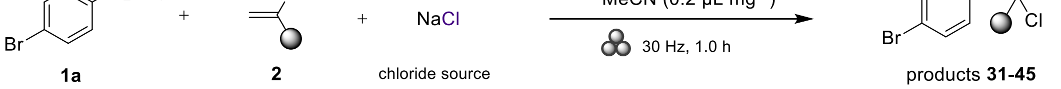

In a nitrogen-filled glove box, NaCl (17.5 mg, 0.3 mmol, 1.5 eq.), **1a** (81.3 mg, 0.3 mmol, 1.5 eq.), **2** (0.2 mmol, 1.0 eq.), and dry MeCN (0.2 µL mg^{-1}) were placed in a ball milling vessel (stainless, 1.5 mL) loaded with one grinding ball (stainless, diameter: 5.0 mm). Then the vessel was closed and placed in the ball mill (Retsch MM400, 30 Hz). After grinding for 60 min, the mixture was passed through a short celite column eluting with EtOAc to remove inorganic salts. The solvent was evaporated and the residual was purified by column chromatography on silica gel with hexane/ethyl acetate as the eluent to give the desired products **31** ~ **45**.

2-(2-(4-bromophenyl)-1-chloroethyl)-4,4,5,5-tetramethyl-1,3,2-dioxaborolane (31)

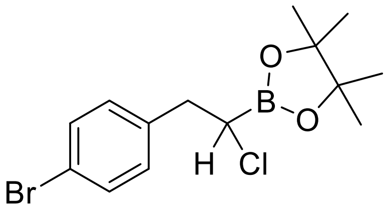

Prepared according to general procedure C using pinacol vinylboronate (30.8 mg, 0.20 mmol). **31** (48.7 mg, 0.141 mmol, 71%) was purified by flash column chromatography (eluent: hexane/ethyl acetate = 10:1) as yellow oil.

^{1}H NMR (400 MHz, $CDCl_3$) δ 7.40 (d, J = 8.4 Hz, 2H), 7.13 (d, J = 8.3 Hz, 2H), 3.54 (t, J = 8.0 Hz, 1H), 3.12 (dd, J = 14.0, 7.6 Hz, 1H), 3.02 (dd, J = 14.0, 8.5 Hz, 1H), 1.23 (s, 6H), 1.23 (s, 6H).

^{13}C NMR (101 MHz, $CDCl_3$) δ 137.40, 131.43, 131.00, 120.70, 84.62, 39.54, 24.62, 24.52.

^{11}B NMR (128 MHz, $CDCl_3$) δ 30.51.

HRMS (ESI) m/z calcd for $C_{14}H_{19}BBrO_2$ [M-Cl]$^+$: 309.06615, found 309.06621.

2-(1-chloro-2-phenylethyl)-4,4,5,5-tetramethyl-1,3,2-dioxaborolane (32)

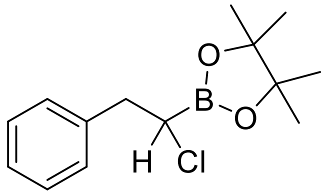

Prepared according to general procedure C, replacing **1a** with **1h** (57.6 mg, 0.30 mmol, 1.5 eq.) and using pinacol vinylboronate (30.8 mg, 0.20 mmol). **32** (23.0 mg, 0.086 mmol, 43%) was purified by flash column chromatography (eluent: hexane/ethyl acetate = 10:1) as pale-yellow oil.

^{1}H NMR (400 MHz, $CDCl_3$) δ 7.23 – 7.16 (m, 5H), 3.52 (t, J = 8.2 Hz, 1H), 3.10 (dd, J = 13.7, 8.1 Hz, 1H), 3.02 (dd, J = 13.8, 8.3 Hz, 1H), 1.16 (s, 6H), 1.15 (s, 6H).

^{13}C NMR (101 MHz, $CDCl_3$) δ 138.38, 129.24, 128.35, 126.76, 84.47, 40.26, 24.57, 24.51.

^{11}B NMR (128 MHz, $CDCl_3$) δ 30.35.

Spectral data match those previously reported[39].

2-(2-(4-bromophenyl)-1-chloro-1-phenylethyl)-4,4,5,5-tetramethyl-1,3,2-dioxaborolane (33)

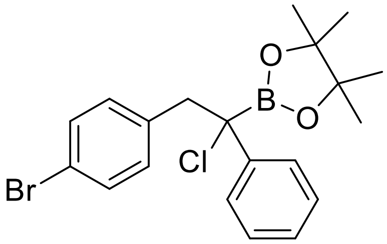

Prepared according to general procedure C using 4,4,5,5-tetramethyl-2-(1-phenylvinyl)-1,3,2-

dioxaborolane (46.0 mg, 0.20 mmol). **33** (50.6 mg, 0.120 mmol, 60%) as a pale-yellow oil. **33** was found to be highly cumbersome due to their instability on silica gel, were obtained in its ketone **33'** form through $NaBO_3 \cdot 4H_2O$ oxidation according to general procedure B.

2-(4-bromophenyl)-1-phenylethan-1-one (33')

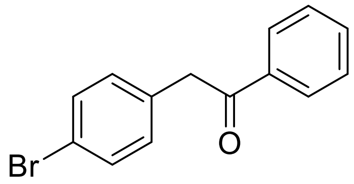

Prepared according to general procedure B using compound **33**, **33'** (16.1 mg, 0.059 mmol, 49%) was purified by flash column chromatography (eluent: hexane/ethyl acetate = 5:1) as white solids.

^{1}H NMR (400 MHz, CDCl$_3$) δ 7.92 (d, J = 7.3 Hz, 2H), 7.50 (t, J = 7.4 Hz, 1H), 7.39 (dd, J = 10.8, 8.1 Hz, 4H), 7.06 (d, J = 8.3 Hz, 2H), 4.17 (s, 2H).

^{13}C NMR (101 MHz, CDCl$_3$) δ 197.00, 136.40, 133.46, 133.39, 131.76, 131.28, 128.75, 128.53, 120.99, 44.77.

Spectral data match those previously reported[85].

2-(1-(4-bromophenyl)-2-chloro-4-phenylbutan-2-yl)-4,4,5,5-tetramethyl-1,3,2-dioxaborolane (34)

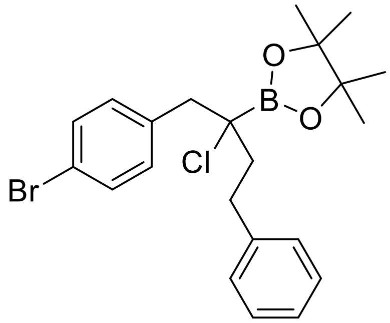

Prepared according to general procedure C using **2d** (51.6 mg, 0.20 mmol). **34** (69.6 mg, 0.155 mmol, 77%) was purified by flash column chromatography (eluent: hexane/ethyl acetate = 10:1) as colourless oil.

^{1}H NMR (400 MHz, CDCl$_3$) δ 7.32 (d, J = 8.4 Hz, 2H), 7.22 – 7.11 (m, 5H), 7.09 (d, J = 7.8 Hz, 2H), 3.07 (d, J = 1.9 Hz, 2H), 2.85 – 2.65 (m, 2H), 2.13 – 1.85 (m, 2H), 1.19 (s, 6H), 1.17 (s, 6H).

^{13}C NMR (101 MHz, CDCl$_3$) δ 141.85, 136.16, 132.50, 131.17, 128.57, 126.10, 121.02, 85.00, 45.07, 41.83, 32.25, 25.01, 24.91, 24.80.

^{11}B NMR (128 MHz, CDCl$_3$) δ 29.87.

HRMS (ESI) m/z calcd for $C_{22}H_{27}BLiBrClO_2$ $[M+Li]^+$: 455.11306, found 455.11805.

2-(4-(benzyloxy)-1-(4-bromophenyl)-2-chlorobutan-2-yl)-4,4,5,5-tetramethyl-1,3,2-dioxaborolane (35)

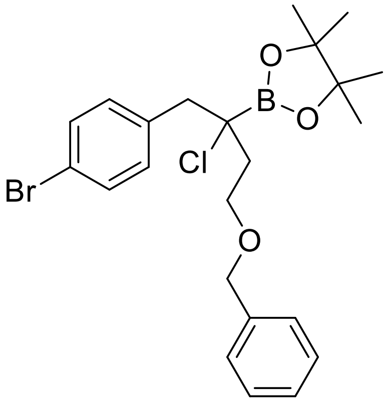

Prepared according to general procedure C using **2e** (57.6 mg, 0.20 mmol). **35** (82.0 mg, 0.171 mmol, 85%) as colourless oil.

^{1}H NMR (400 MHz, CDCl$_3$) δ 7.37 (d, *J* = 8.4 Hz, 2H), 7.33 (d, *J* = 4.1 Hz, 5H), 7.21 (d, *J* = 8.3 Hz, 2H), 4.51 (s, 2H), 3.61 (t, *J* = 6.7 Hz, 2H), 3.13 (d, *J* = 14.1 Hz, 1H), 3.05 (d, *J* = 14.2 Hz, 1H), 2.09 (ddd, *J* = 92.3, 14.2, 7.5 Hz, 2H), 1.20 (s, 6H), 1.15 (s, 6H).

^{13}C NMR (101 MHz, CDCl$_3$) δ 138.24, 135.85, 132.71, 130.89, 128.38, 128.29, 127.81, 127.66, 127.62, 120.86, 84.72, 73.08, 66.87, 45.32, 39.38, 24.90, 24.75, 24.63.

^{11}B NMR (128 MHz, CDCl$_3$) δ 30.36.

HRMS (ESI) m/z calcd for $C_{23}H_{29}BNaBrClO_3$ [M+Na]$^+$: 501.09739, found 501.09891.

1-bromo-4-(2-chloro-2-phenylethyl)benzene (36)

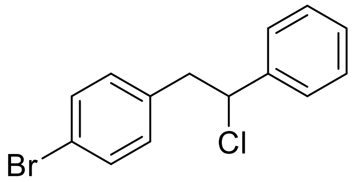

Prepared according to general procedure C using styrene (20.8 mg, 0.20 mmol). **36** (54.4 mg, 0.184 mmol, 92%) was purified by flash column chromatography (eluent: hexane/ethyl acetate = 10:1) as yellow solids.

^{1}H NMR (400 MHz, CDCl$_3$) δ 7.37 (d, *J* = 8.4 Hz, 2H), 7.35 – 7.28 (m, 5H), 6.97 (d, *J* = 8.2 Hz, 2H), 5.00 (t, *J* = 7.3 Hz, 1H), 3.35 (dd, *J* = 14.0, 7.8 Hz, 1H), 3.28 (dd, *J* = 14.0, 6.9 Hz, 1H).

^{13}C NMR (101 MHz, CDCl$_3$) δ 140.82, 136.43, 131.53, 131.26, 128.65 (d, *J* = 14.7 Hz), 127.20, 63.74, 45.96.

HRMS (ESI) m/z calcd for $C_{14}H_{12}LiBrCl$ [M+Li]$^+$: 300.99655, found 300.99684.

1-bromo-4-(2-chloro-2-(p-tolyl)ethyl)benzene (37)

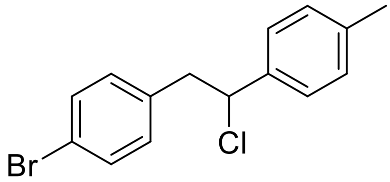

Prepared according to general procedure C using 4-methylstyrene (23.6 mg, 0.20 mmol). **37** (18.1 mg, 0.058 mmol, 29%) was purified by flash column chromatography (eluent: hexane/ethyl acetate = 10:1) as colourless oil.

^{1}H NMR (400 MHz, CDCl$_3$) δ 7.30 (d, *J* = 8.3 Hz, 2H), 7.14 (d, *J* = 8.2 Hz, 2H), 7.06 (d, *J* = 7.9 Hz, 2H), 6.90 (d, *J* = 8.3 Hz, 2H), 4.90 (t, *J* = 7.3 Hz, 1H), 3.30 – 3.17 (m, 2H), 2.27 (s, 3H).
^{13}C NMR (101 MHz, CDCl$_3$) δ 138.37, 137.82, 136.52, 131.43, 131.17, 129.32, 127.02, 120.81, 63.67, 45.79, 21.18.
HRMS (ESI) m/z calcd for $C_{15}H_{14}KBrCl$ [M+K]$^+$: 346.95990, found 346.96075.

1-bromo-4-(2-chloro-2-(4-(trifluoromethyl)phenyl)ethyl)benzene (38)

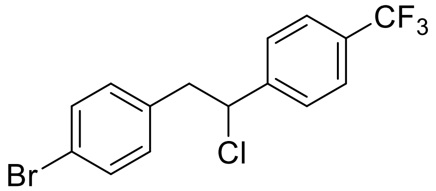

Prepared according to general procedure C using 1-(trifluoromethyl)-4-vinylbenzene (34.4 mg, 0.20 mmol). **38** (61.1 mg, 0.168 mmol, 84%) was purified by flash column chromatography (eluent: hexane/ethyl acetate = 10:1) as yellow oil.
^{1}H NMR (400 MHz, CDCl$_3$) δ 7.51 (d, *J* = 8.2 Hz, 2H), 7.35 (d, *J* = 8.1 Hz, 2H), 7.31 (d, *J* = 8.2 Hz, 2H), 6.87 (d, *J* = 8.2 Hz, 2H), 4.95 (t, *J* = 7.3 Hz, 1H), 3.26 (dd, *J* = 14.0, 7.6 Hz, 1H), 3.18 (dd, *J* = 14.1, 6.9 Hz, 1H).
^{13}C NMR (101 MHz, CDCl$_3$) δ 144.55, 144.54, 135.64, 131.61, 131.13, 127.57, 125.65 (q, *J* = 3.7 Hz), 121.17, 62.45, 45.78.
^{19}F NMR (376 MHz, CDCl$_3$) δ -62.63.
HRMS (ESI) m/z calcd for $C_{15}H_{11}NaBrClF_3$ [M+Na]$^+$: 384.95770, found 384.95844.

1-(2-(4-bromophenyl)-1-chloroethyl)-3,5-bis(trifluoromethyl)benzene (39)

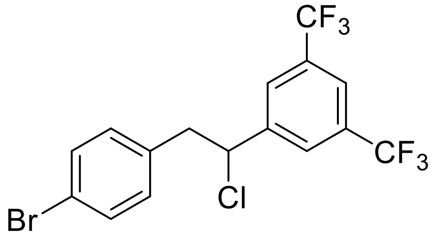

Prepared according to general procedure C using 1-ethenyl-3,5-bis(trifluoromethyl)benzene (48.0 mg, 0.20 mmol). **39** (52.5 mg, 0.122 mmol, 61%) was purified by flash column chromatography (eluent: hexane/ethyl acetate = 10:1) as pale-yellow oil.
^{1}H NMR (400 MHz, CDCl$_3$) δ 7.71 (d, *J* = 32.0 Hz, 3H), 7.34 (d, *J* = 8.2 Hz, 2H), 6.89 (d, *J* = 8.2 Hz, 2H), 5.01 (t, *J* = 7.2 Hz, 1H), 3.28 (dd, *J* = 14.1, 8.0 Hz, 1H), 3.19 (dd, *J* = 14.1, 6.5 Hz, 1H).
^{13}C NMR (101 MHz, CDCl$_3$) δ 146.02, 143.26, 137.38, 134.95, 132.23, 132.04, 131.90, 131.78, 131.59, 131.07, 130.61, 129.63, 127.42, 127.40, 122.47, 122.44, 122.40, 122.36, 122.33, 121.50, 61.58, 52.11, 45.69, 41.08.
^{19}F NMR (376 MHz, CDCl$_3$) δ -62.92.
HRMS (ESI) m/z calcd for $C_{16}H_{10}NaBrClF_6$ [M+Na]$^+$: 452.94508, found 452.94681.

2-(2-(4-bromophenyl)-1-chloroethyl)-1,3-dichlorobenzene (40)

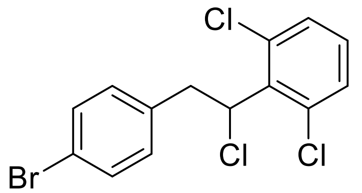

Prepared according to general procedure C using 2,6-dichlorostyrene (34.6 mg, 0.20 mmol). **40** (37.2 mg, 0.102 mmol, 51%) was purified by flash column chromatography (eluent: hexane/ethyl acetate = 10:1) as yellow oil.

^{1}H NMR (400 MHz, CDCl$_3$) δ 7.29 (d, *J* = 8.4 Hz, 2H), 7.20 – 7.11 (m, 2H), 7.06 (t, *J* = 8.0 Hz, 1H), 6.98 (d, *J* = 8.4 Hz, 2H), 5.86 (t, *J* = 8.0 Hz, 1H), 3.61 (dd, *J* = 13.9, 8.2 Hz, 1H), 3.52 (dd, *J* = 14.0, 8.0 Hz, 1H).

^{13}C NMR (101 MHz, CDCl$_3$) δ 136.14, 135.78, 135.17, 134.28, 131.55, 130.92, 130.73, 129.82, 128.46, 121.04, 57.47, 41.02.

HRMS (ESI) m/z calcd for $C_{14}H_{10}BrCl_2$ [M-Cl]$^+$: 326.93429, found 326.93454.

(2-(4-bromophenyl)-1-chloroethyl)dimethyl(phenyl)silane (41)

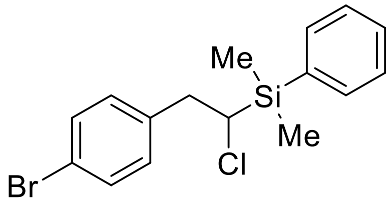

Prepared according to general procedure C using dimethylphenylvinylsilane (32.5 mg, 0.20 mmol). **41** (42.1 mg, 0.119 mmol, 60%) was purified by flash column chromatography (eluent: hexane/ethyl acetate = 10:1) as yellow oil.

^{1}H NMR (400 MHz, CDCl$_3$) δ 7.57 (d, *J* = 5.7 Hz, 2H), 7.44 – 7.35 (m, 5H), 7.01 (d, *J* = 8.3 Hz, 2H), 3.58 (dd, *J* = 11.8, 3.3 Hz, 1H), 3.00 (dd, *J* = 14.9, 3.3 Hz, 1H), 2.73 (dd, *J* = 14.8, 11.8 Hz, 1H), 0.45 (d, *J* = 8.5 Hz, 6H).

^{13}C NMR (101 MHz, CDCl$_3$) δ 138.52, 135.43, 134.23, 131.40, 130.75, 129.90, 128.15, 120.48, 50.97, 38.98, -4.40, -5.70.

HRMS (ESI) m/z calcd for $C_{16}H_{19}BrClSi$ [M+H]$^+$: 353.01224, found 353.01499.

(2-(4-bromophenyl)-1-chloroethyl)trimethylsilane (42)

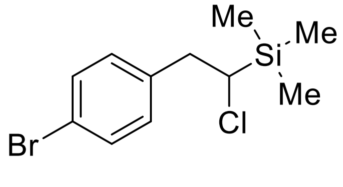

Prepared according to general procedure C using vinyltrimethylsilane (20.0 mg, 0.20 mmol). **42** (24.3 mg, 0.083 mmol, 42%) was purified by flash column chromatography (eluent: hexane/ethyl acetate = 10:1) as colourless oil.

^{1}H NMR (400 MHz, CDCl$_3$) δ 7.29 (d, *J* = 8.4 Hz, 2H), 6.97 (d, *J* = 8.4 Hz, 2H), 3.26 (dd, *J* = 11.7, 3.6 Hz, 1H), 2.93 (dd, *J* = 14.7, 3.6 Hz, 1H), 2.65 (dd, *J* = 14.7, 11.7 Hz, 1H), 0.00 (s, 9H).

^{13}C NMR (101 MHz, CDCl$_3$) δ 138.60, 131.39, 130.68, 120.40, 51.50, 38.96, -3.47.

HRMS (ESI) m/z calcd for $C_{11}H_{16}NaBrClSi$ [M+Na]$^+$: 312.97854, found 312.97809.

3-(4-bromophenyl)-2-chloro-2-methyl-*N*-phenylpropanamide (43)

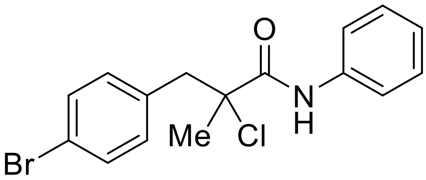

Prepared according to general procedure C using *N*-phenylmethacrylamide (32.2 mg, 0.20 mmol). **43** (40.4 mg, 0.115 mmol, 57%) was purified by flash column chromatography (eluent: hexane/ethyl acetate = 10:1) as pale-yellow oil.

^{1}H NMR (400 MHz, CDCl$_3$) δ 8.53 (s, 1H), 7.67 – 7.60 (m, 4H), 7.56 (t, *J* = 7.9 Hz, 2H), 7.41 – 7.34 (m, 3H), 3.77 (d, *J* = 14.0 Hz, 1H), 3.32 (d, *J* = 14.0 Hz, 1H), 2.16 (s, 3H).

^{13}C NMR (101 MHz, CDCl$_3$) δ 168.83, 136.82, 134.46, 132.49, 131.30, 129.15, 125.30, 121.62, 120.44, 74.07, 47.61, 30.22.

HRMS (ESI) m/z calcd for $C_{16}H_{16}BrClNO$ [M+H]$^+$: 352.00983, found 352.01072.

3.4 Gram-scale experiment for synthesis of α-chloroboronic esters

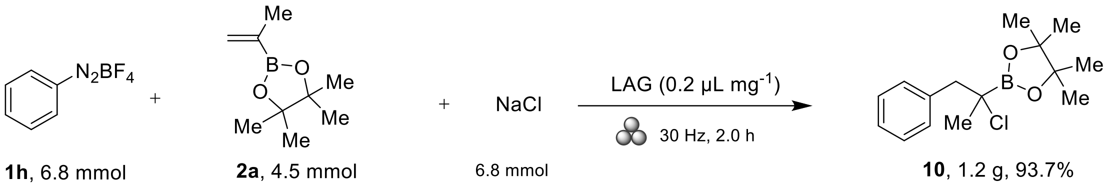

In a nitrogen-filled glove box, NaCl (394.5 mg, 6.8 mmol, 1.5 eq.), **1h** (1.3 g, 6.8 mmol, 1.5 eq.), **2a** (756.2 mg, 4.5 mmol, 1.0 eq.), and dry MeCN (0.2 μL mg^{-1}) were placed in a ball milling vessel (stainless, 10.0 mL) loaded with nine grinding balls (stainless, diameter: 7.0 mm). Then the vessel was closed and placed in the ball mill (Retsch MM400, 30 Hz). After grinding for 2 hours, the mixture was passed through a celite column eluting with EtOAc to remove inorganic salts. The solvent was evaporated and the residual was purified by column chromatography on silica gel with hexane/ethyl acetate as the eluent to give the desired products **10** (1.2 g, 4.2 mmol, 94%).

3.5 Radical trapping experiment

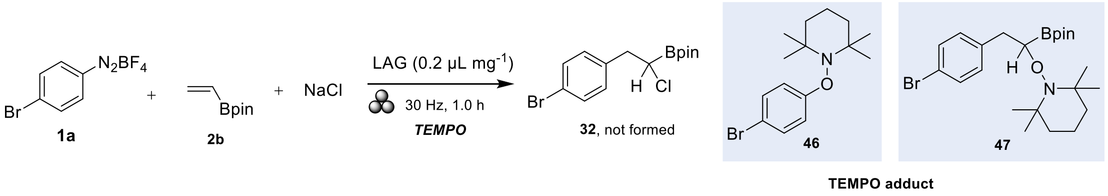

In a nitrogen-filled glove box, NaCl (17.5 mg, 0.3 mmol, 1.5 eq.), **1a** (81.3 mg, 0.3 mmol, 1.5 eq.), **2b** (30.8 mg, 0.2 mmol, 1.0 eq.), TEMPO (62.5 mg, 0.4 mmol, 2.0 eq.), and dry MeCN (0.2 μL mg^{-1}) were placed in a ball milling vessel (stainless, 1.5 mL) loaded with one grinding ball (stainless, diameter: 5.0 mm). Then the vessel was closed and placed in the ball mill (Retsch MM400, 30 Hz). After grinding for 60 min, the mixture was passed through a short celite column eluting with EtOAc to remove inorganic salts, and the solvent was evaporated to get crude products. The crude product was analyzed by LC-MS to determine the presence of

product (**32**) and/or TEMPO adducts. LC-MS m/z calcd for **46** [$C_{15}H_{22}BrNO$]: 311.09, found 311.80; calcd for **47** [$C_{17}H_{26}BrNO$] $[M+H]^+$: 339.11, found 339.03.

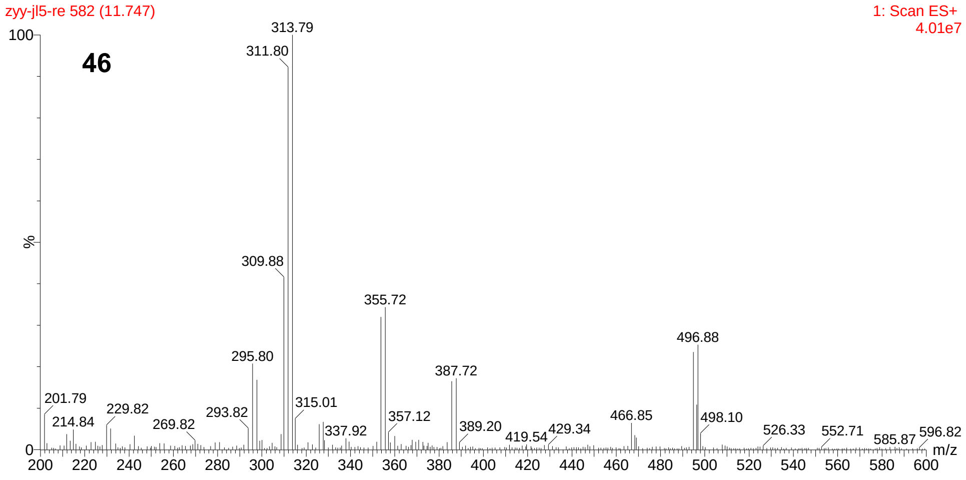

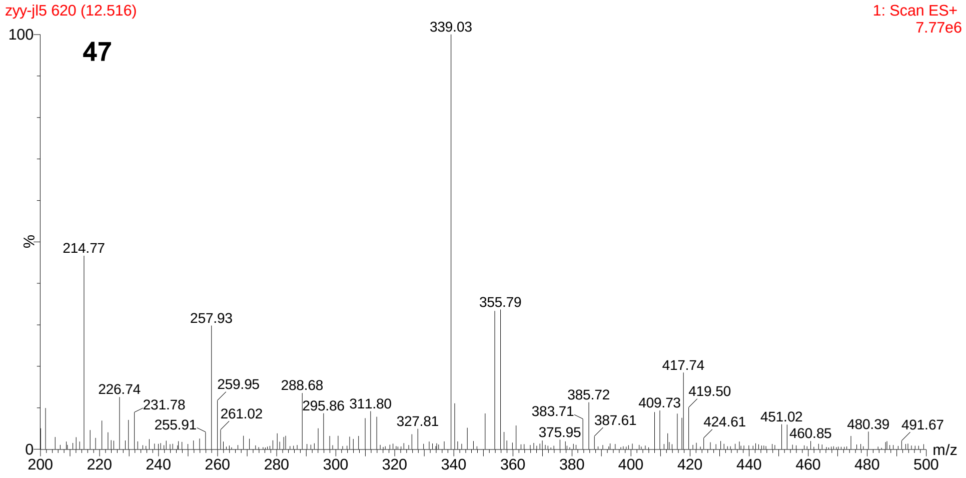

3.6 *Radical clock experiment*

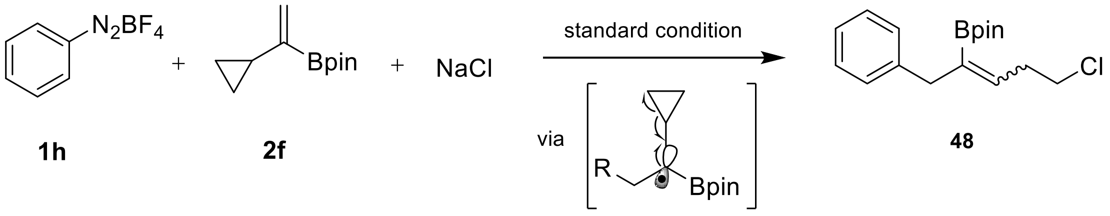

In a nitrogen-filled glove box, NaCl (8.8 mg, 0.15 mmol, 1.5 eq.), **1h** (28.8 mg, 0.15 mmol, 1.5 eq.), **2f** (19.4 mg, 0.10 mmol, 1.0 eq.), and dry MeCN (0.2 μL mg^{-1}) were placed in a ball milling vessel (stainless, 1.5 mL) loaded with one grinding ball (stainless, diameter: 5.0 mm). Then the vessel was closed and placed in the ball mill (Retsch MM400, 30 Hz). After grinding for 60 min, the mixture was passed through a short celite column eluting with EtOAc to remove inorganic salts. The solvent was evaporated and the residual was purified by column chromatography on silica gel with hexane/ethyl acetate as the eluent to give the desired products **48** (10.5 mg, 0.034 mmol, 34%) as a pale-yellow oil. The product was analyzed by LC-MS to determine the

presence of product **48**. LC-MS m/z calcd for **48** [$C_{17}H_{24}BNaClO_2$] $[M+Na]^+$ 329.16, found 328.90.

^{1}H NMR (400 MHz, $CDCl_3$) δ 7.19 (s, 4H), 7.15 – 7.12 (m, 4H), 7.10 (d, *J* = 1.6 Hz, 2H), 7.07 (d, *J* = 8.2 Hz, 2H), 6.30 (t, *J* = 7.0 Hz, 1H), 3.48 – 3.44 (m, 4H), 3.42 (s, 2H), 3.38 (s, 2H), 2.97 – 2.76 (m, 2H), 2.62 (q, *J* = 7.0 Hz, 2H), 1.12 (s, 12H), 1.09 – 1.03 (m, 12H).

Spectral data match those previously reported[39].

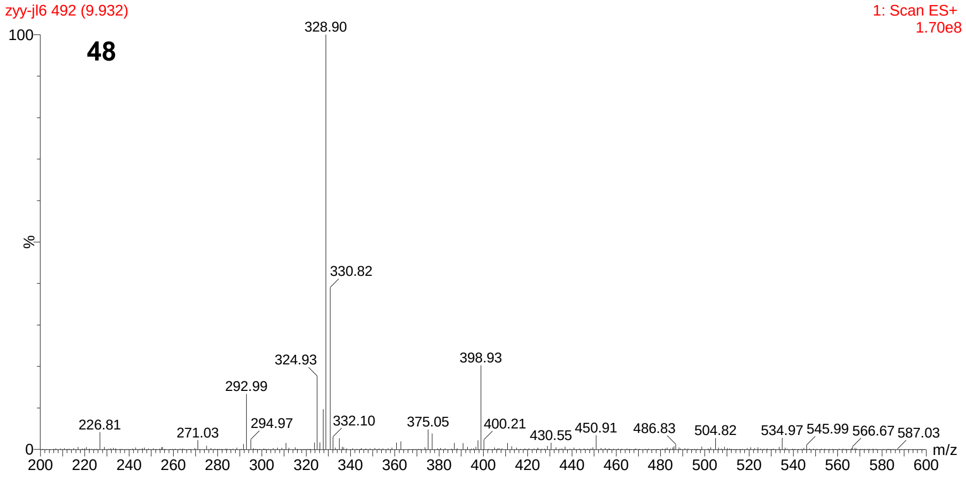

3.7 Ball milling material experiment

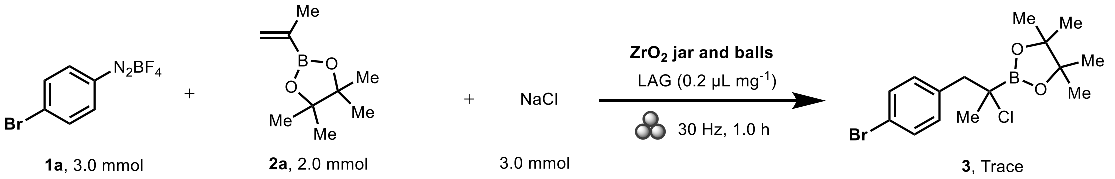

In a nitrogen-filled glove box, NaCl (175.0 mg, 3.0 mmol, 1.5 eq.), **1a** (813 mg, 3.0 mmol, 1.5 eq.), **2a** (336 mg, 2.0 mmol, 1.0 eq.), and dry MeCN (0.2 μL mg^{-1}) were placed in a ball milling vessel (ZrO_2, 10.0 mL) loaded with nine grinding balls (ZrO_2, diameter: 7.0 mm). Then the vessel was closed and placed in the ball mill (Retsch MM400, 30 Hz). After grinding for an hour, the mixture was passed through a celite column eluting with EtOAc to remove inorganic salts and the solvent was evaporated to get crude products. The crude products was analyzed by NMR (400 MHz), only trace of product **3** can be found.

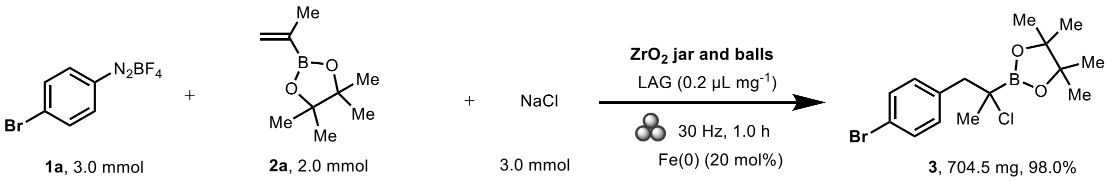

In a nitrogen-filled glove box, NaCl (175.0 mg, 3.0 mmol, 1.5 eq.), **1a** (813 mg, 3.0 mmol, 1.5 eq.), **2a** (336 mg, 2.0 mmol, 1.0 eq.), iron powder (22.4 mg, 0.4 mmol, 20 mol%) and dry MeCN (0.2 μL mg^{-1}) were placed in a ball milling vessel (ZrO_2, 10.0 mL) loaded with nine grinding balls (ZrO_2, diameter: 7.0 mm). Then the vessel was closed and placed in the ball mill (Retsch MM400, 30 Hz). After grinding for an hour, the mixture was passed through a celite column eluting with EtOAc to remove inorganic salts. The solvent was evaporated and the residual was

purified by column chromatography on silica gel with hexane/ethyl acetate as the eluent to give the desired products **3** (704.5 mg, 1.9 mmol, 98%).

4. NMR Spectroscopic Data

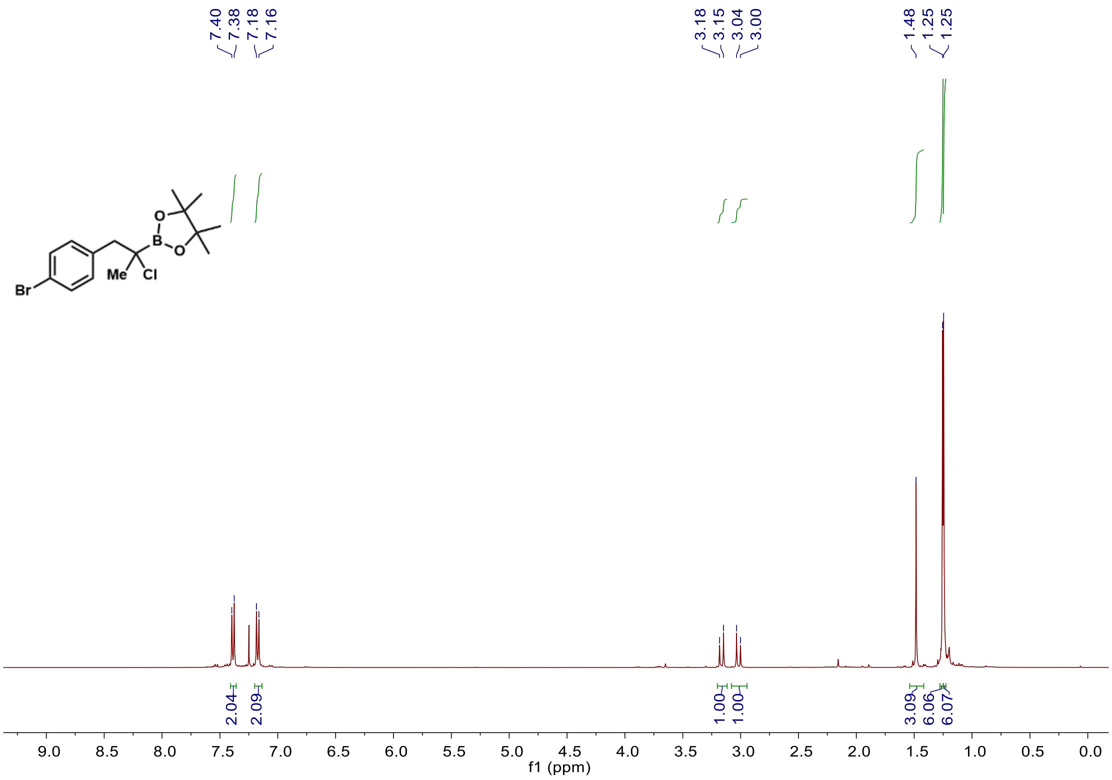

^{1}H NMR spectra (400 MHz, $CDCl_3$) of **3**.

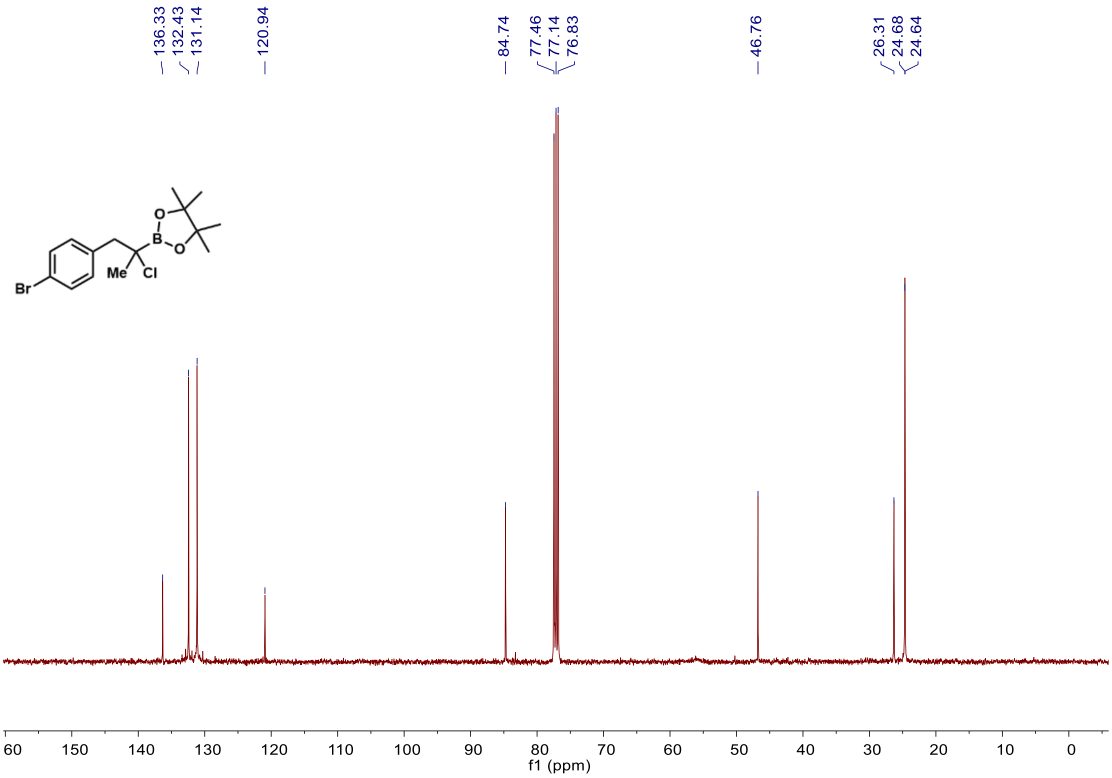

^{13}C NMR spectra (101 MHz, $CDCl_3$) of **3**.

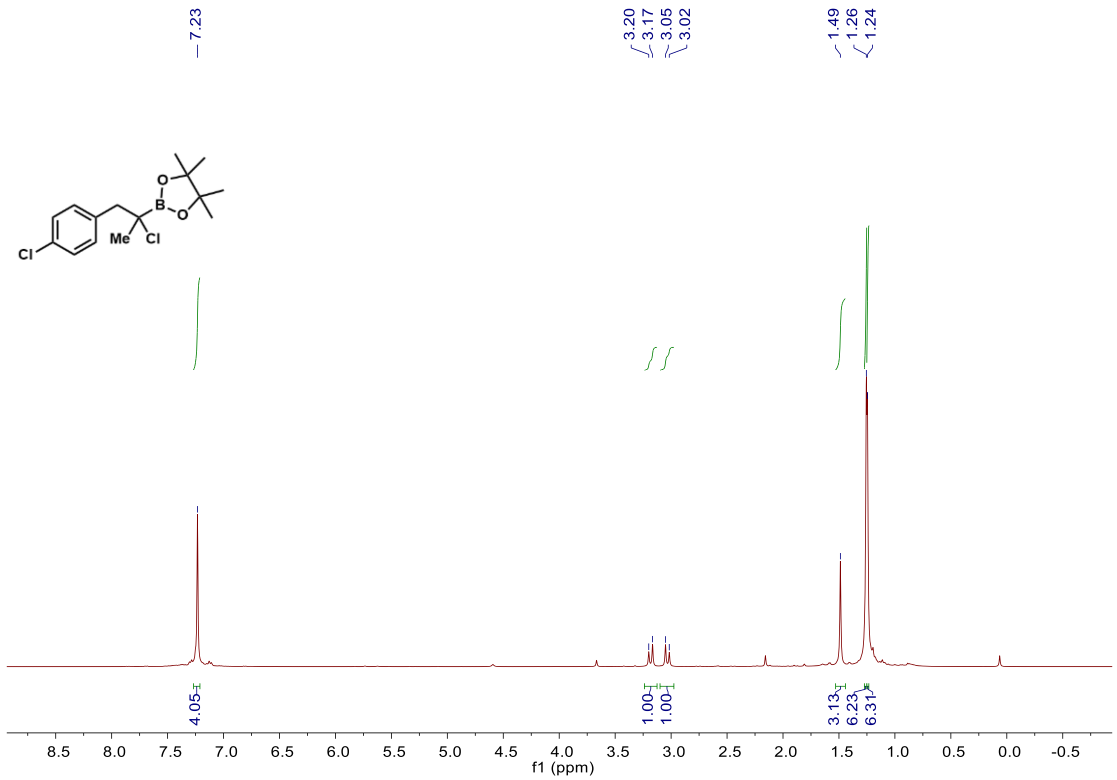

^{1}H NMR spectra (400 MHz, $CDCl_3$) of **4.**

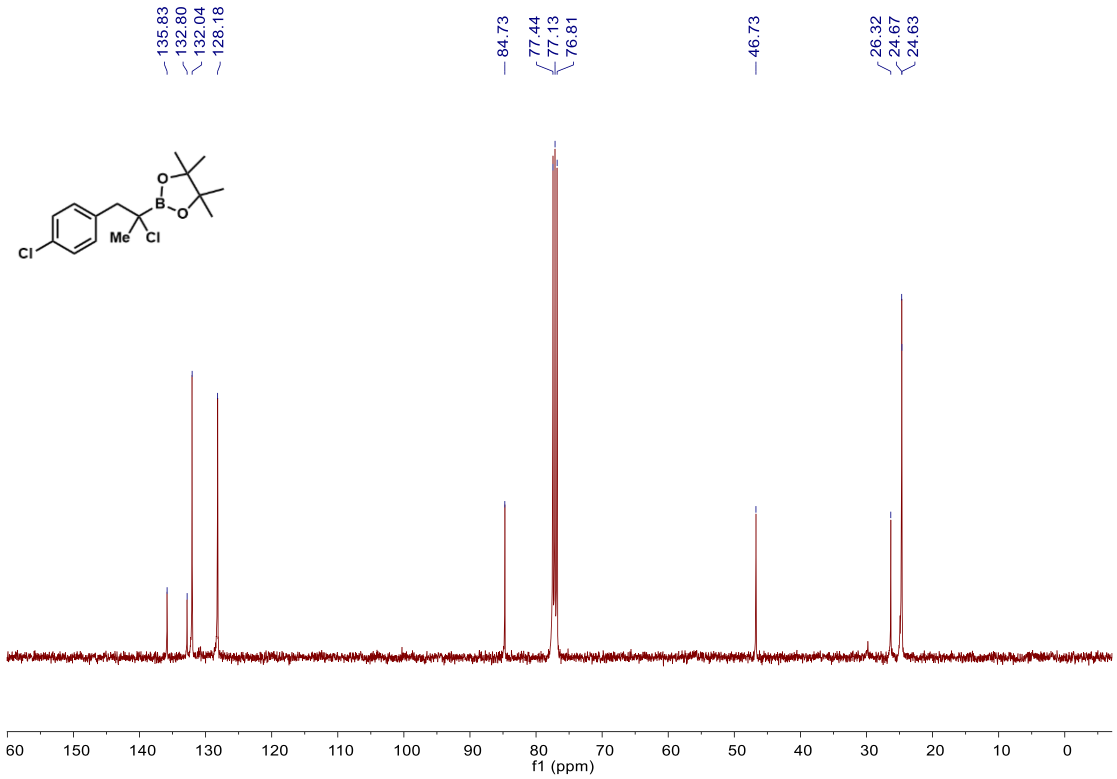

^{13}C NMR spectra (101 MHz, $CDCl_3$) of **4.**

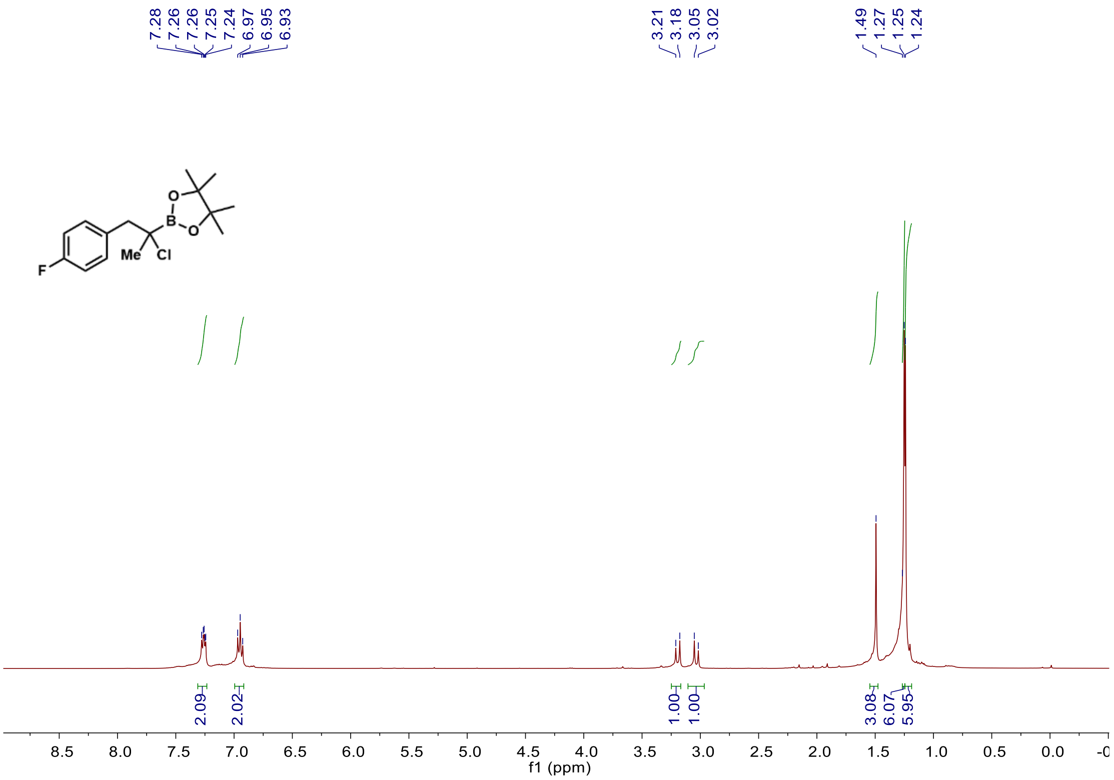

^{1}H NMR spectra (400 MHz, $CDCl_3$) of **5**.

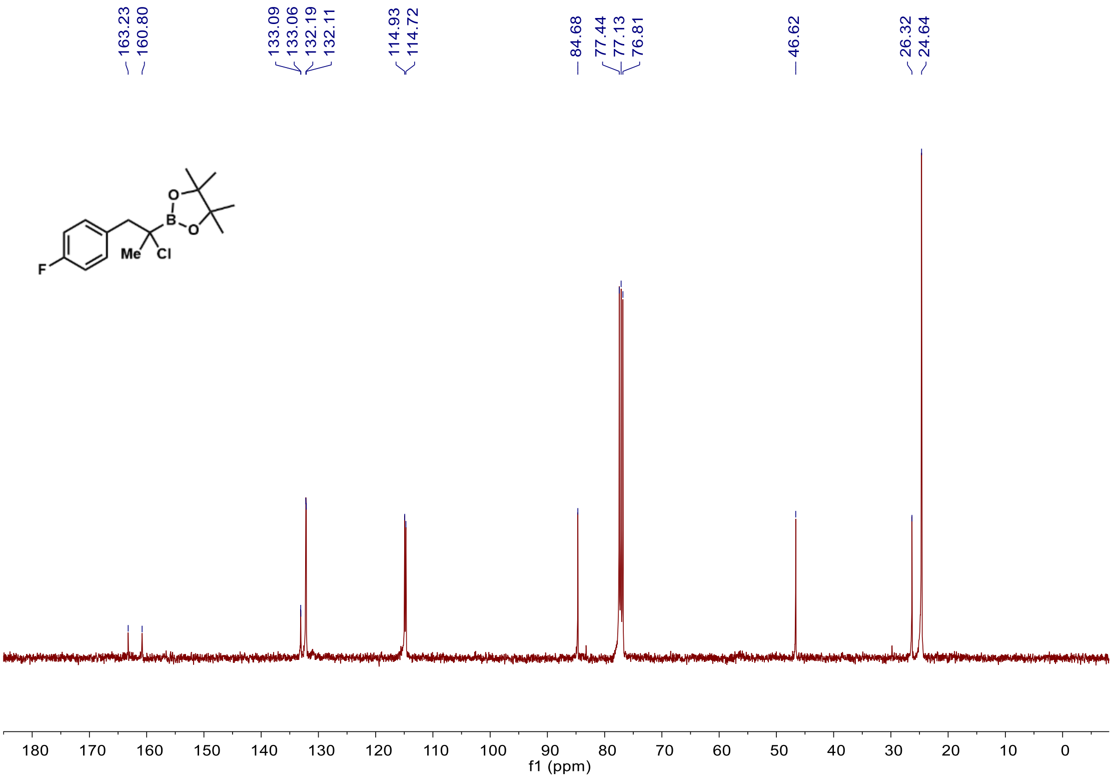

^{13}C NMR spectra (101 MHz, $CDCl_3$) of **5**.

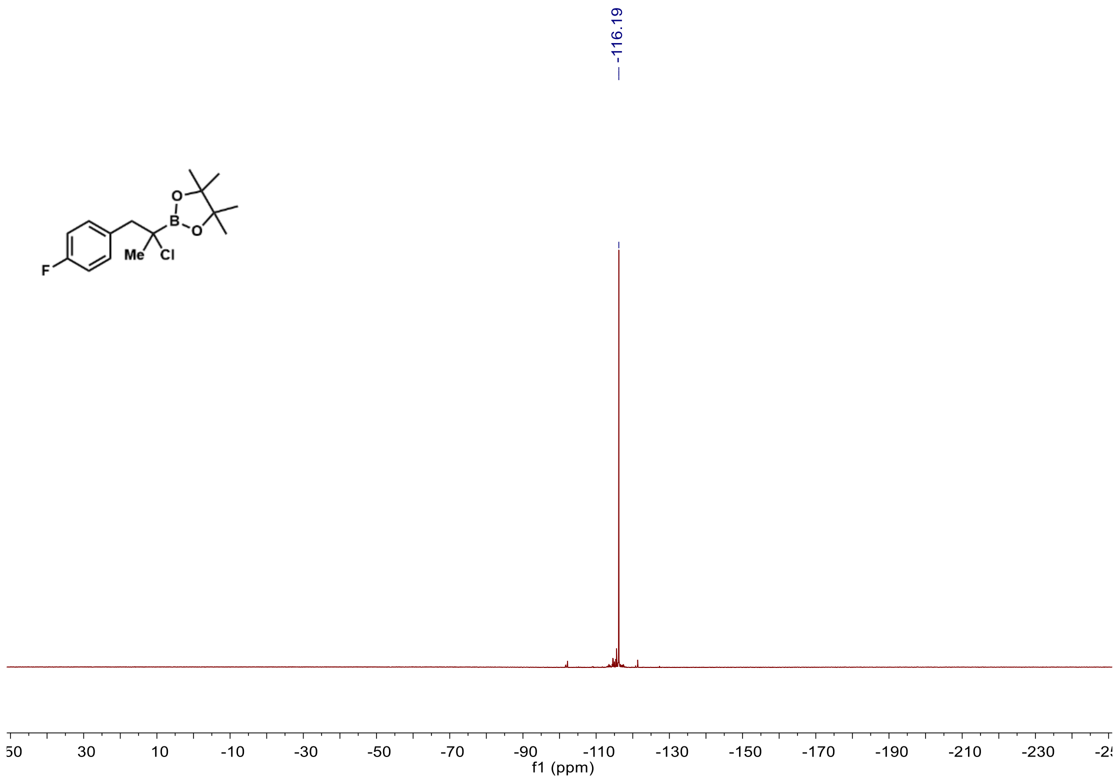

^{19}F NMR spectra (376 MHz, $CDCl_3$) of **5**.

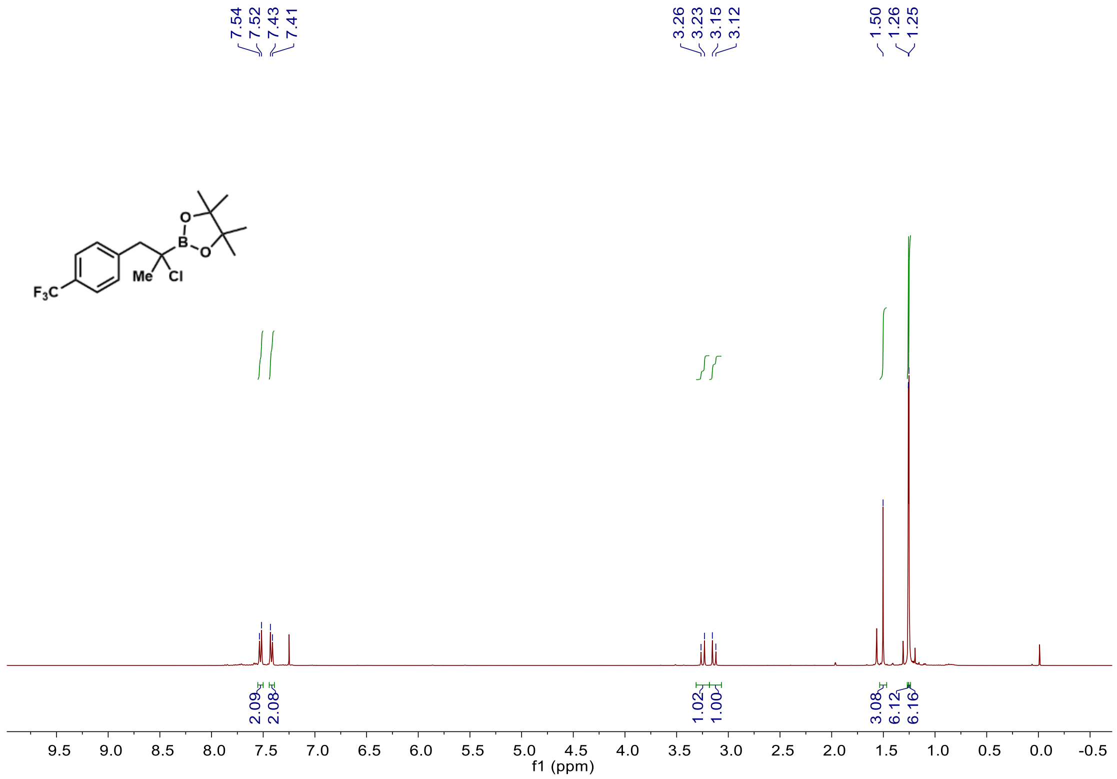

^{1}H NMR spectra (400 MHz, $CDCl_3$) of **7**.

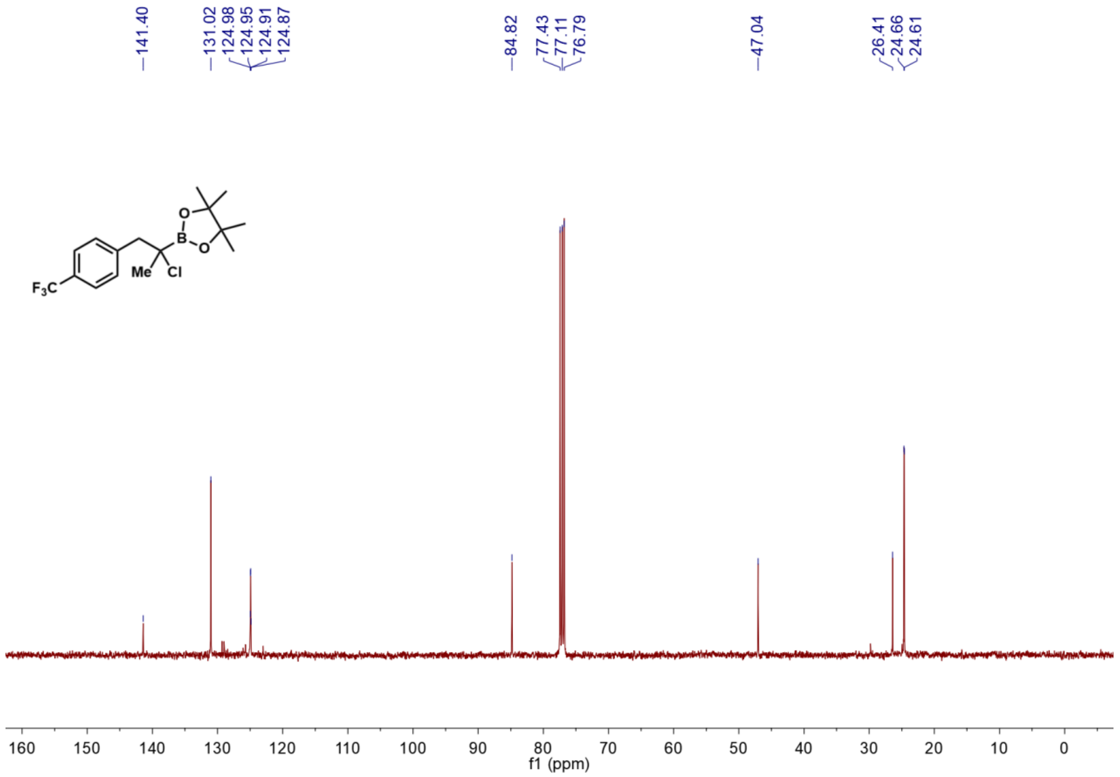

^{13}C NMR spectra (101 MHz, $CDCl_3$) of **7**.

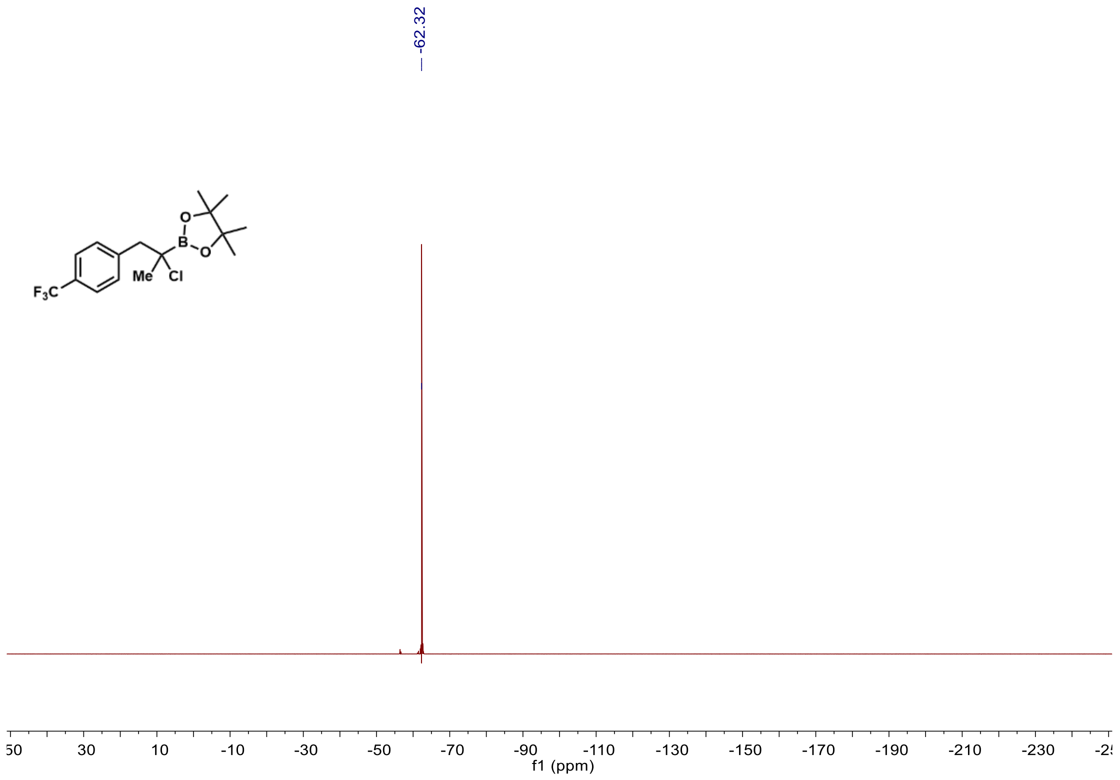

^{19}F NMR spectra (376 MHz, $CDCl_3$) of **7**.

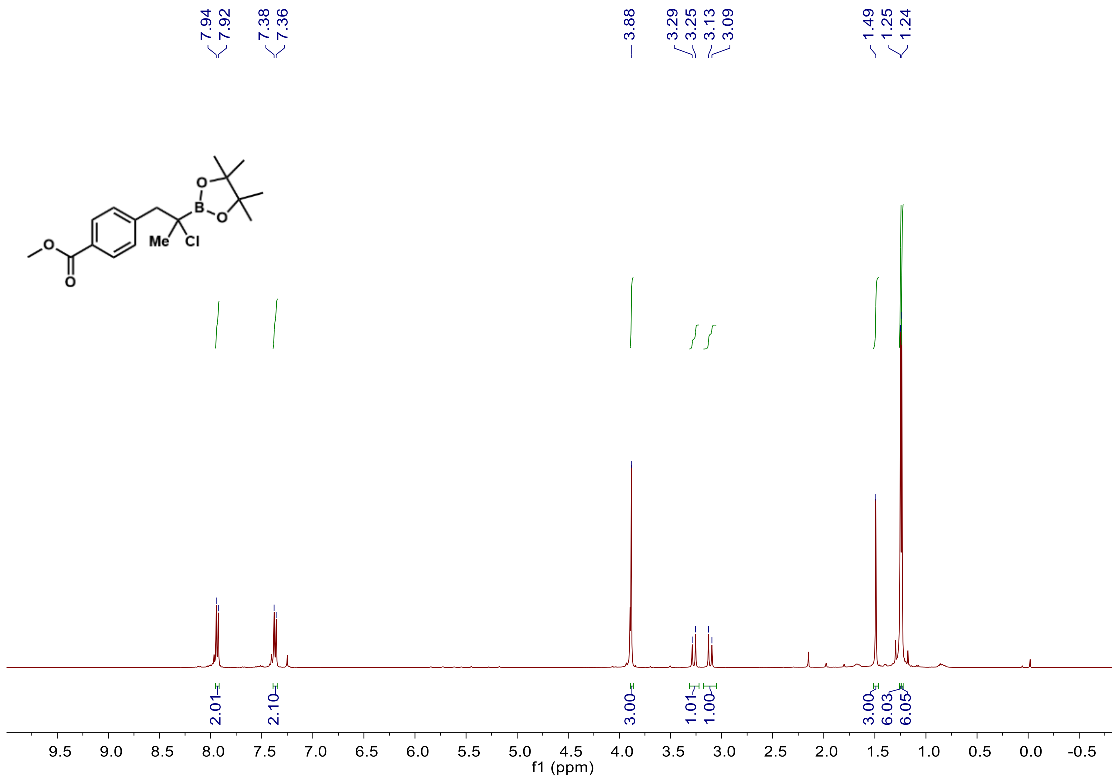

^{1}H NMR spectra (400 MHz, $CDCl_3$) of **8**.

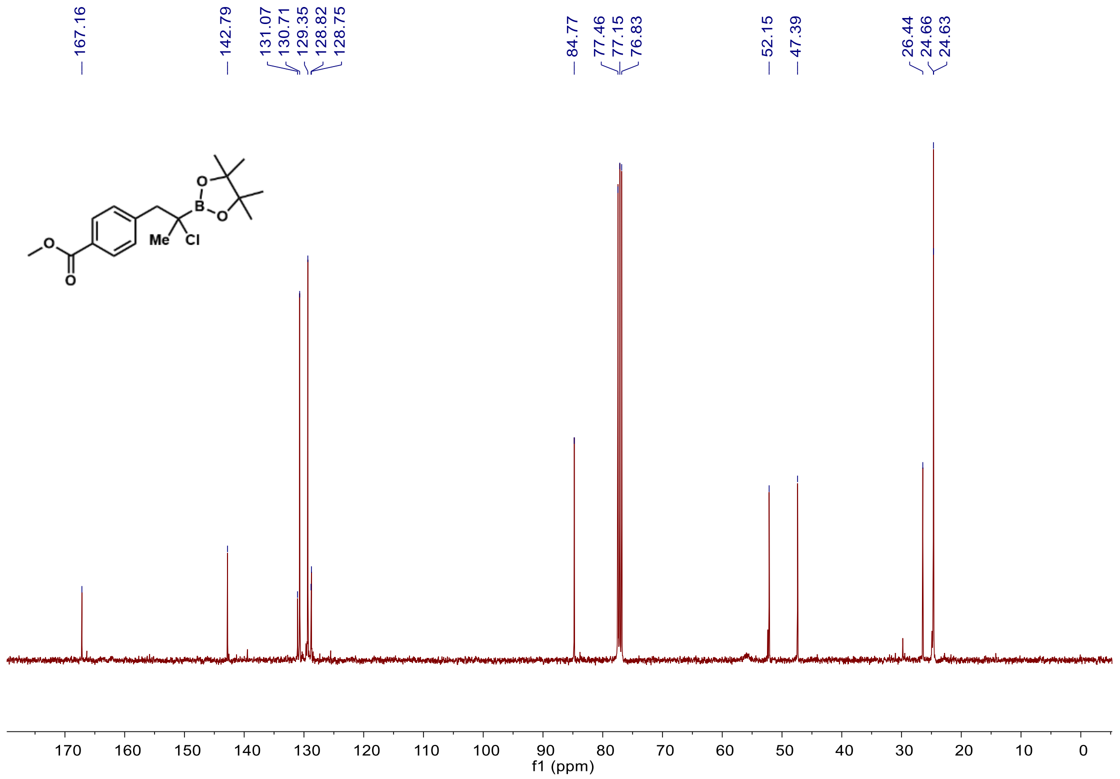

^{13}C NMR spectra (101 MHz, $CDCl_3$) of **8**.

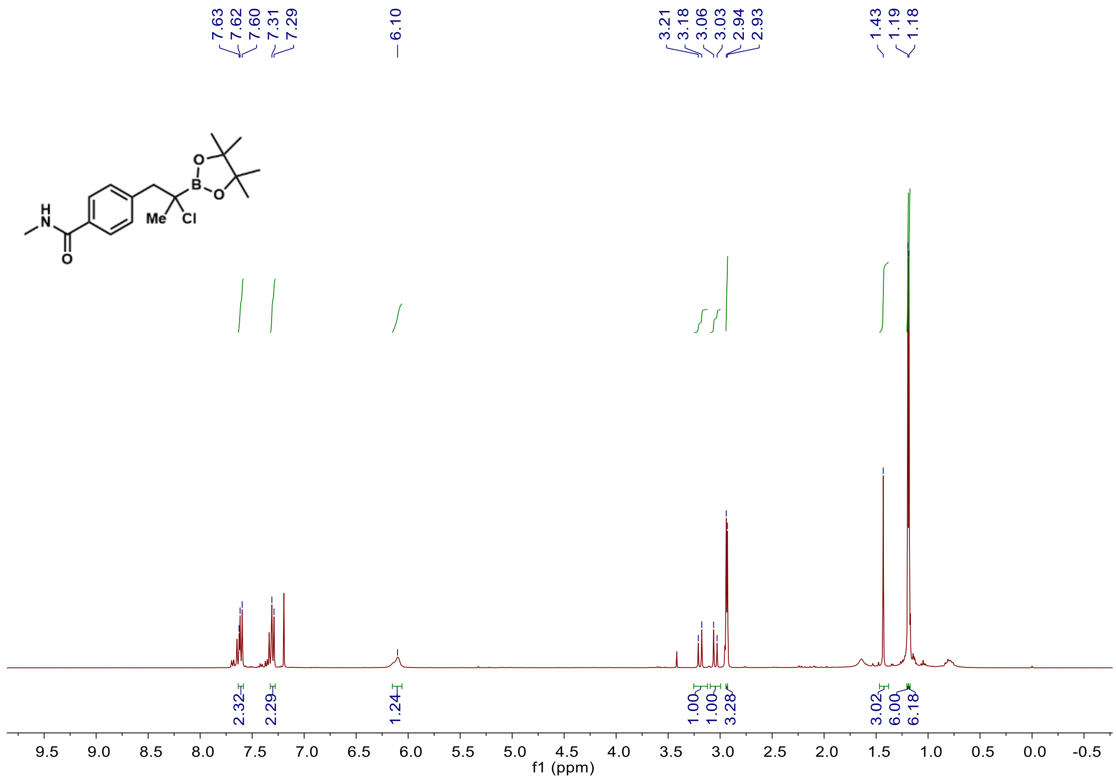

^{1}H NMR spectra (400 MHz, $CDCl_3$) of **9**.

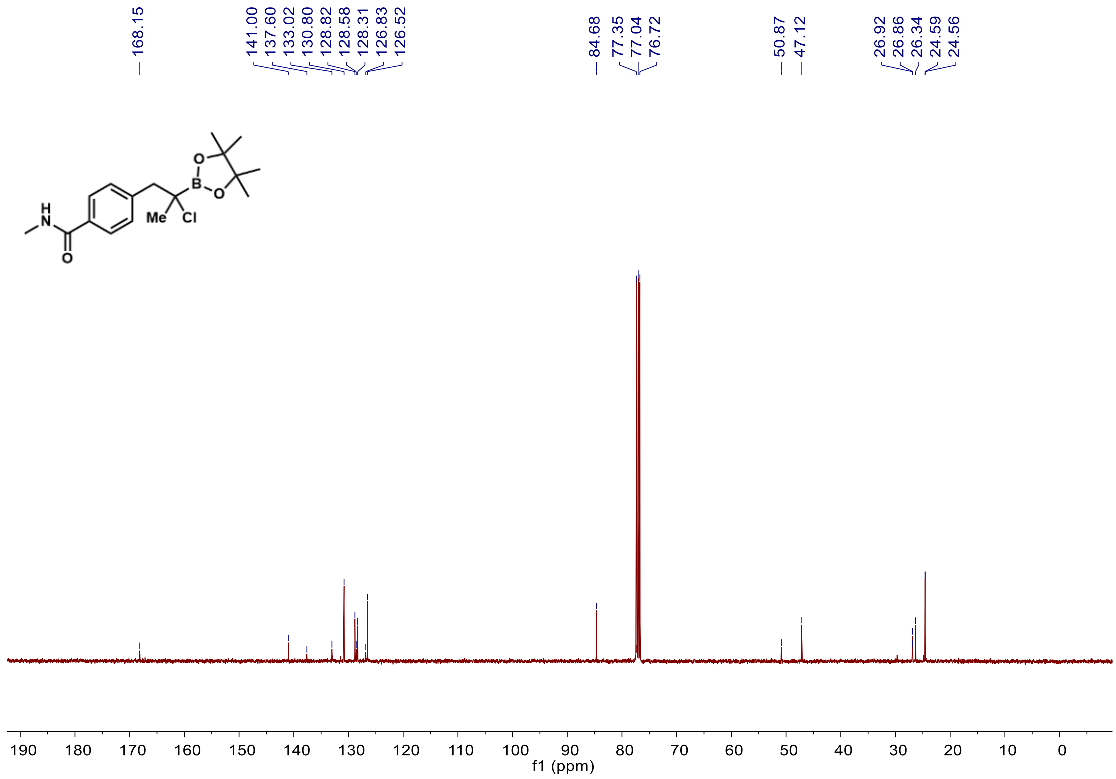

^{13}C NMR spectra (101 MHz, $CDCl_3$) of **9**.

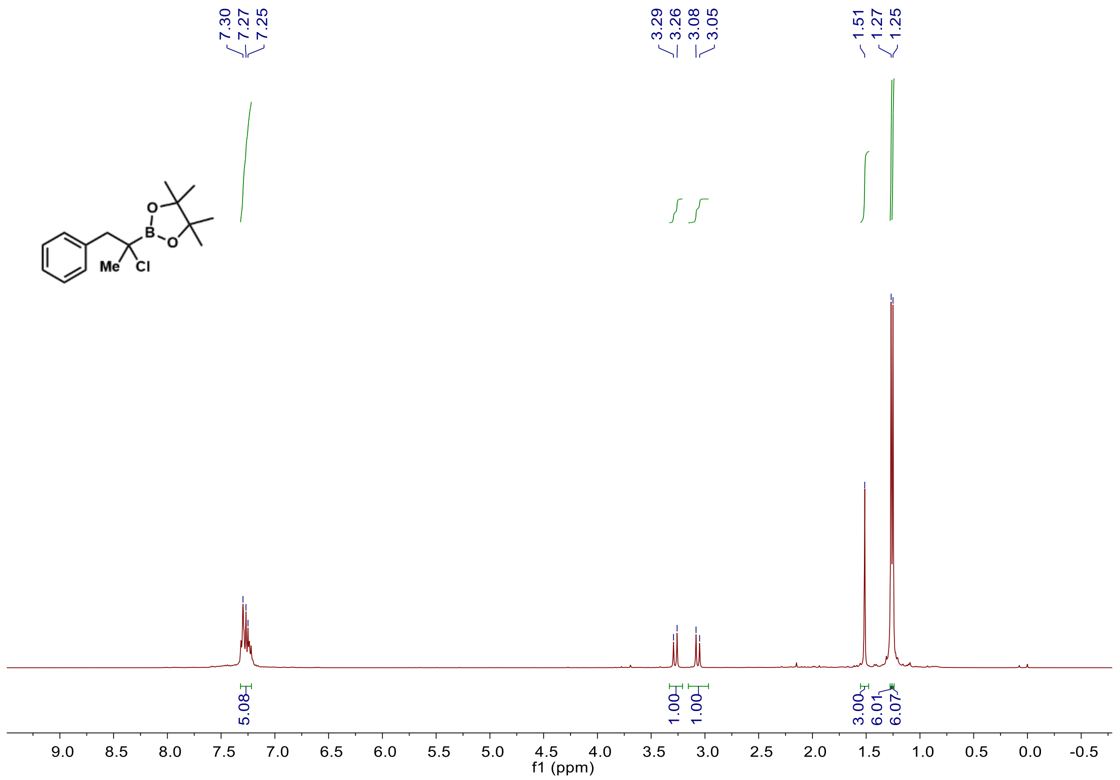

^{1}H NMR spectra (400 MHz, $CDCl_3$) of **10**.

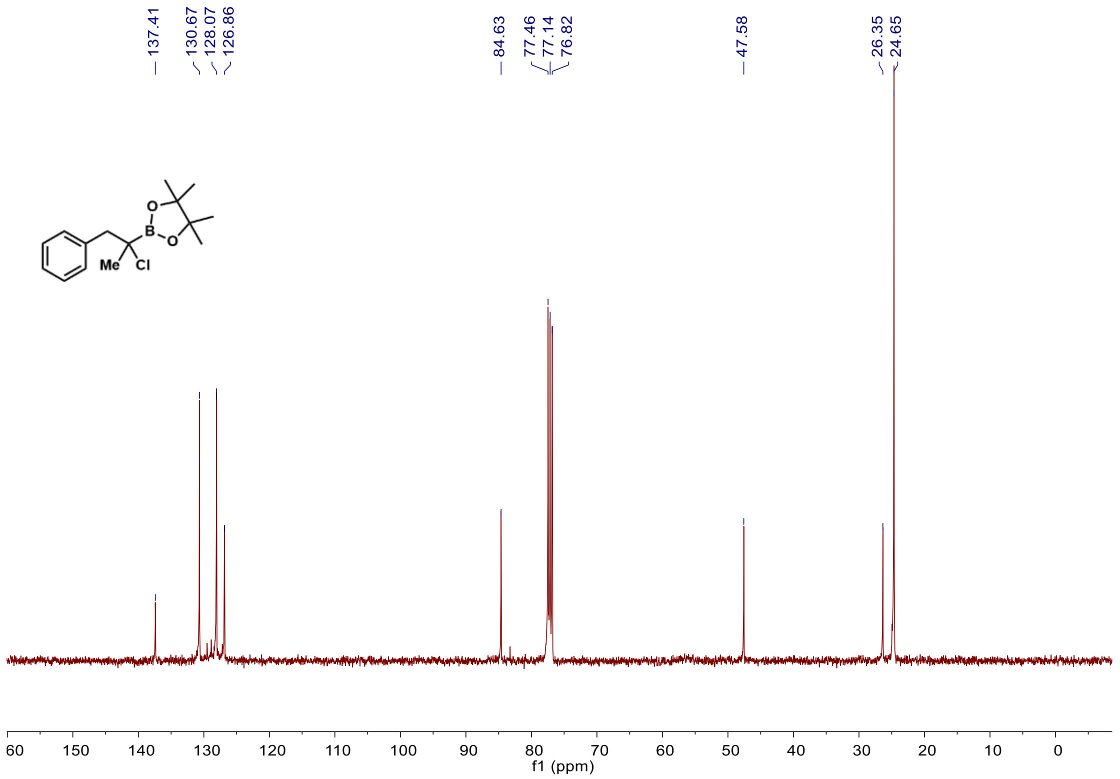

^{13}C NMR spectra (101 MHz, $CDCl_3$) of **10**.

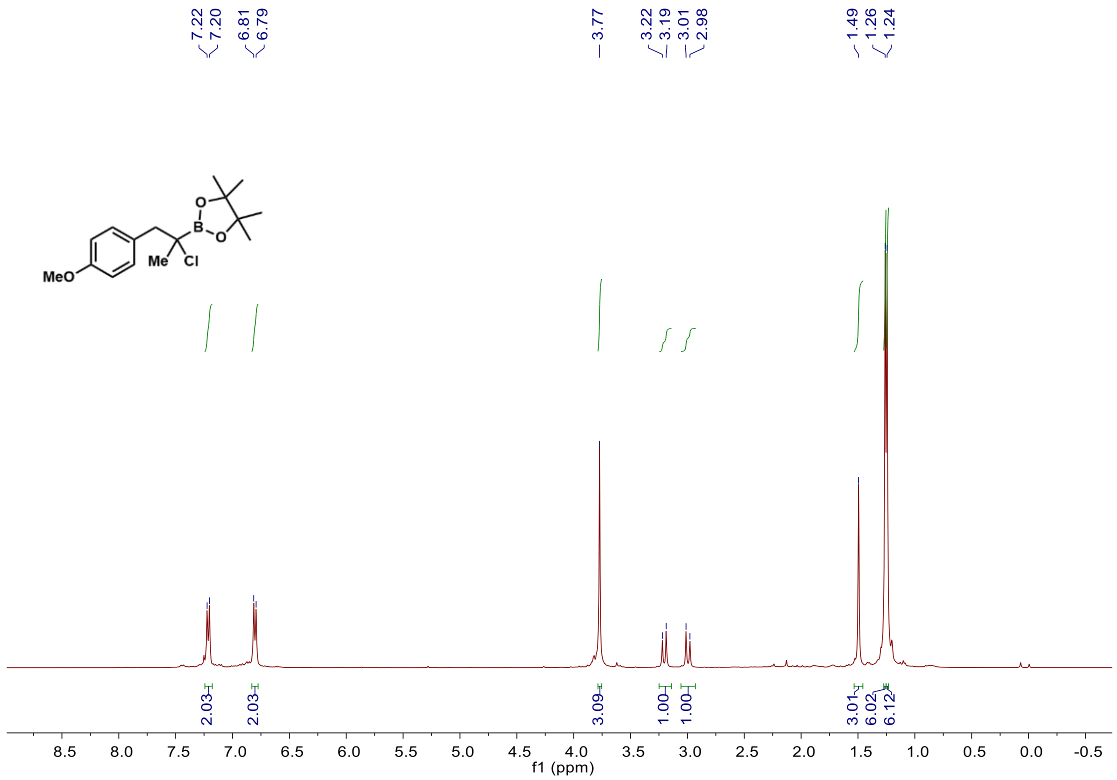

^{1}H NMR spectra (400 MHz, $CDCl_3$) of **11**.

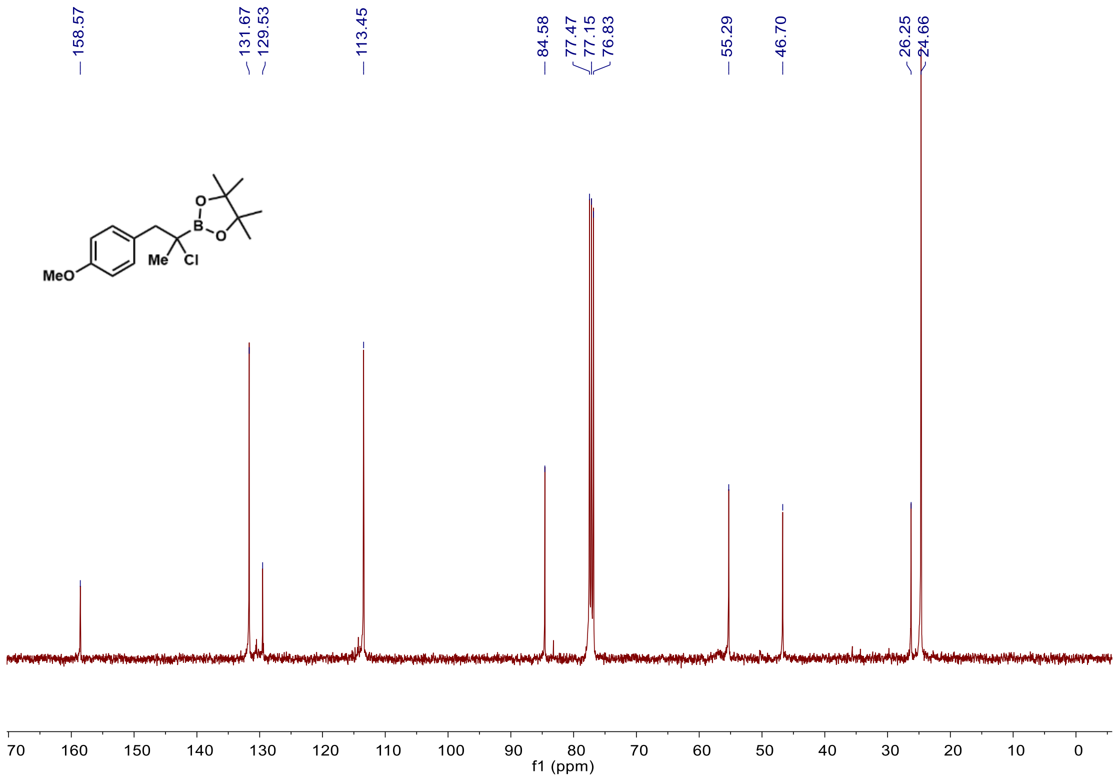

^{13}C NMR spectra (101 MHz, $CDCl_3$) of **11**.

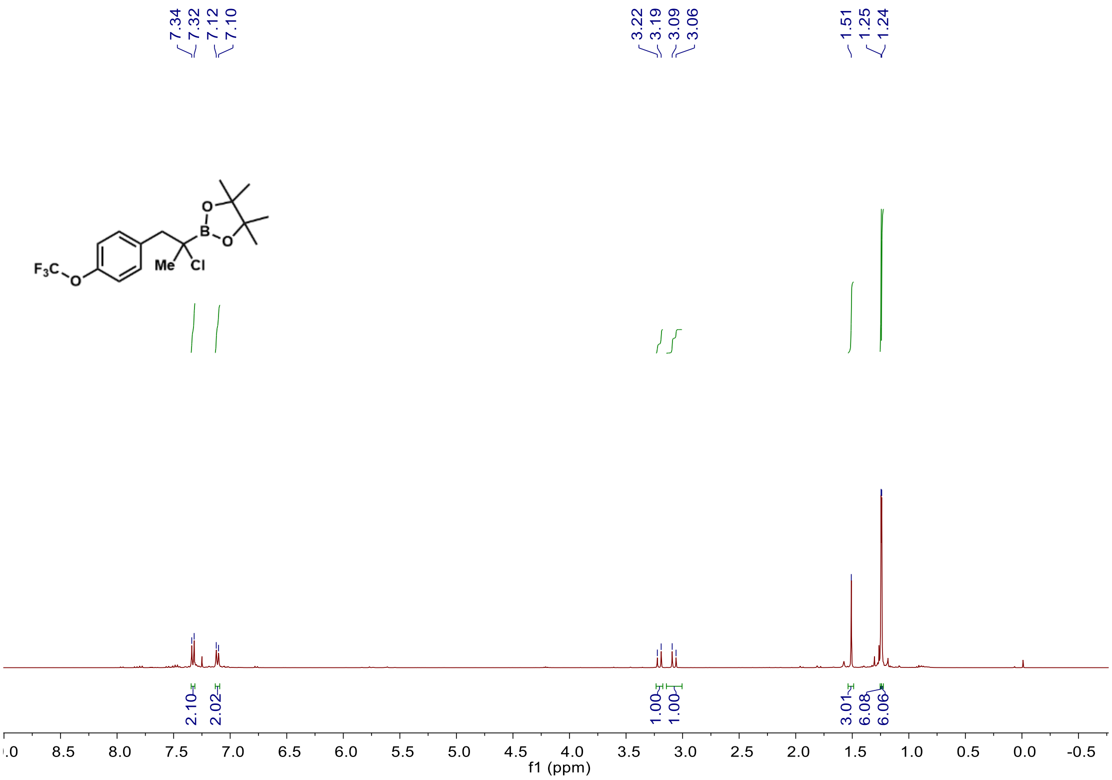

^{1}H NMR spectra (400 MHz, $CDCl_3$) of **12**.

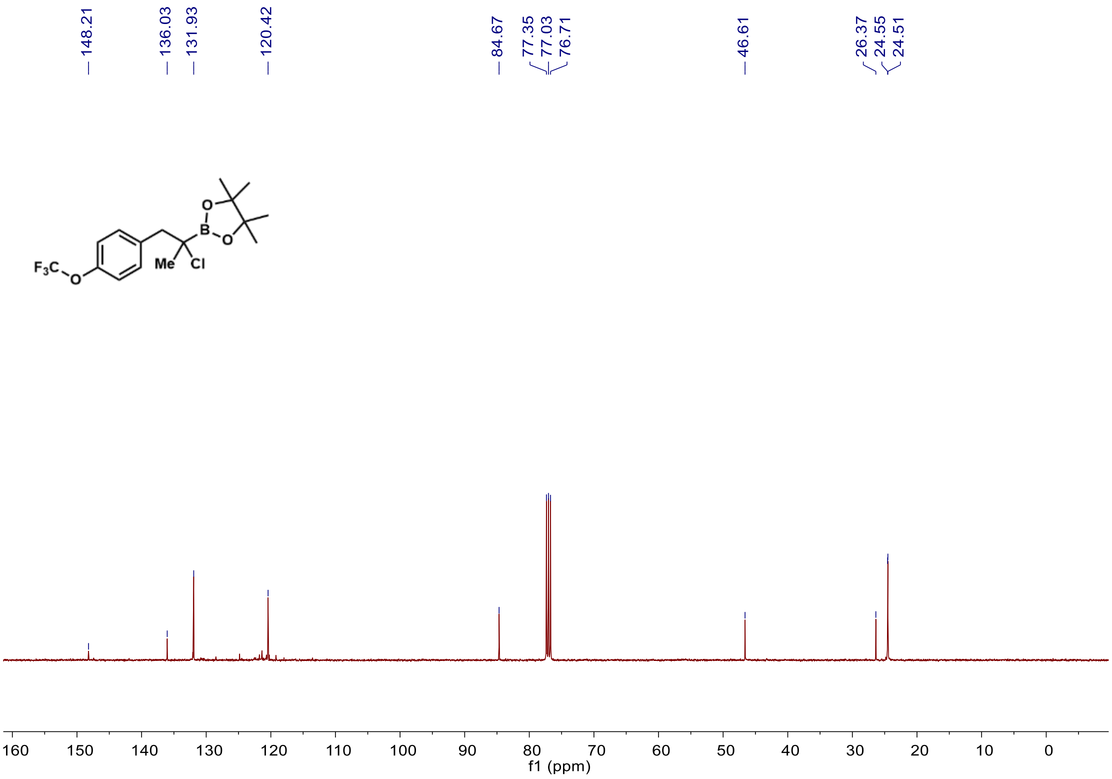

^{13}C NMR spectra (101 MHz, $CDCl_3$) of **12**.

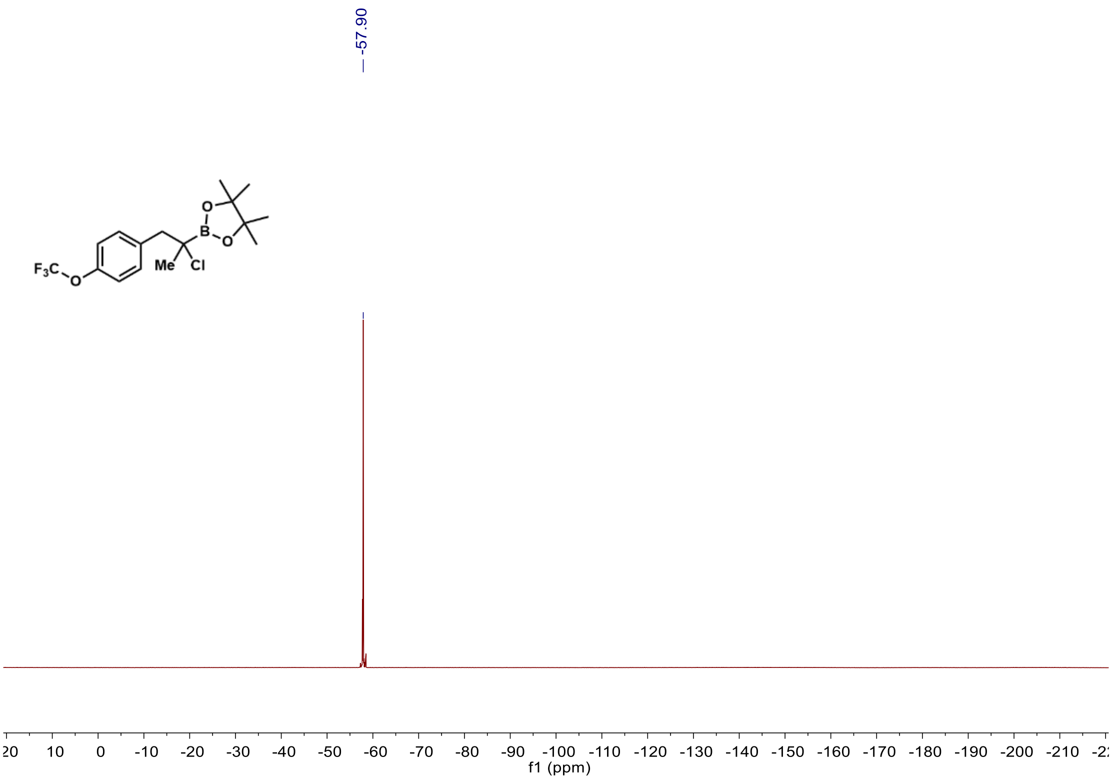

^{19}F NMR spectra (376 MHz, CDCl$_3$) of **12**.

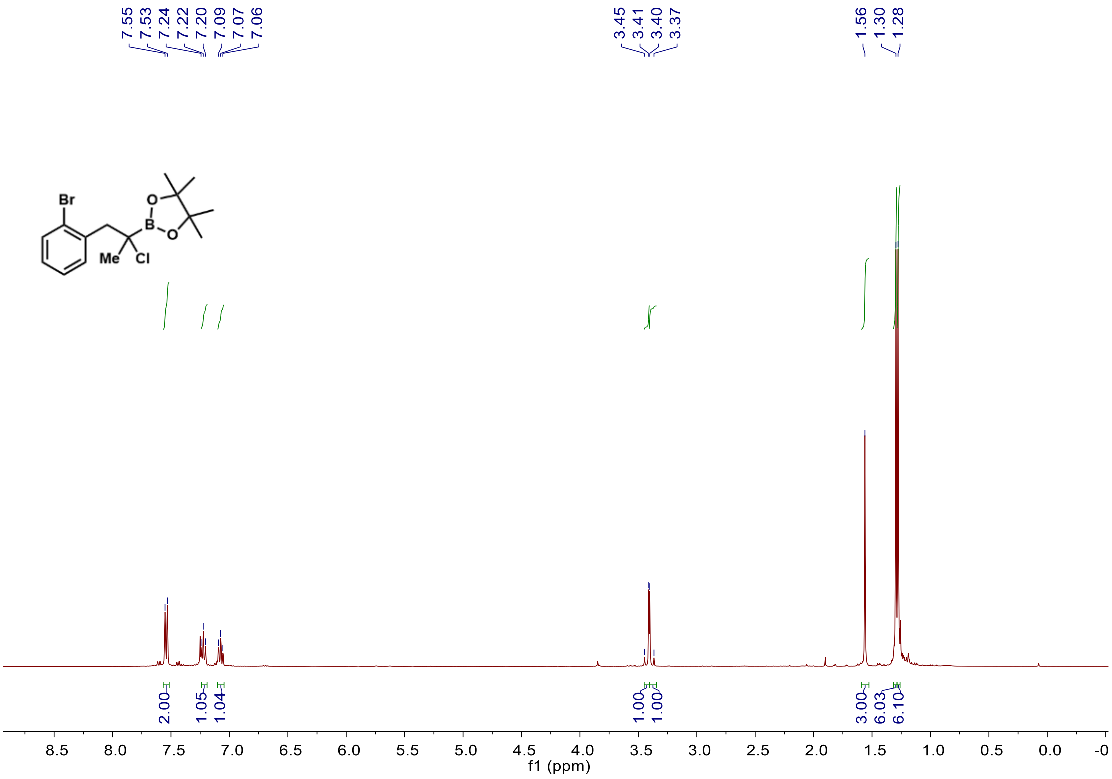

^{1}H NMR spectra (400 MHz, CDCl$_3$) of **16**.

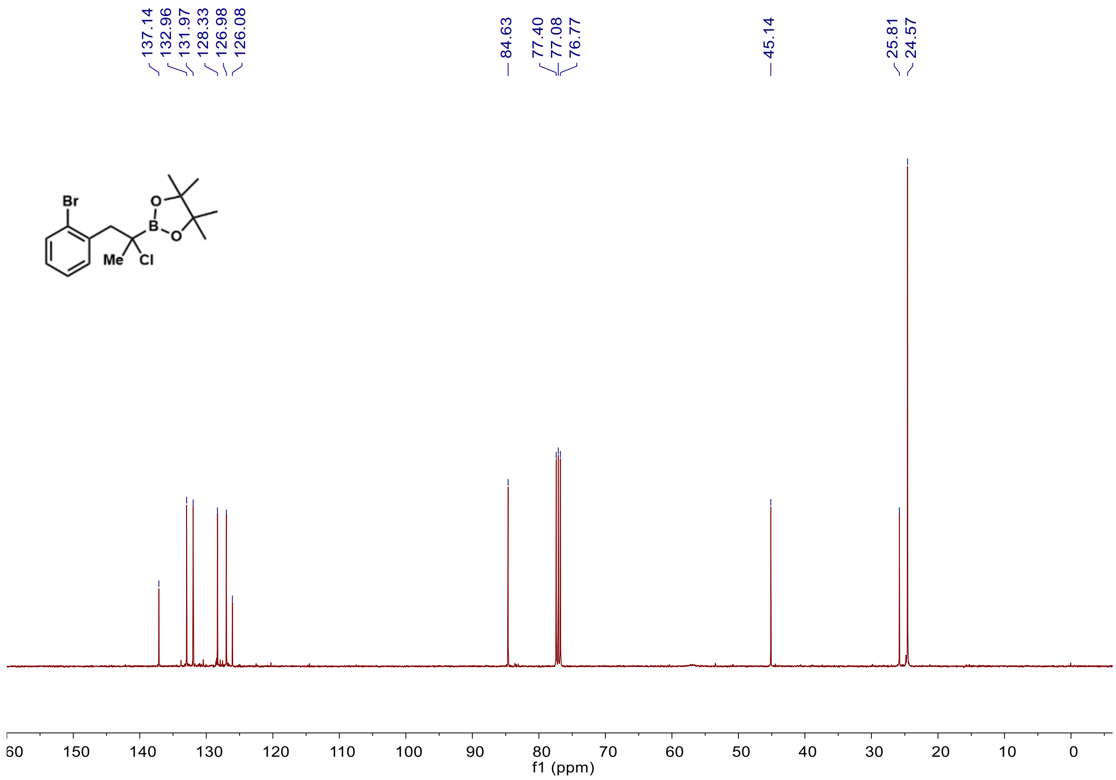

^{13}C NMR spectra (101 MHz, $CDCl_3$) of **16**.

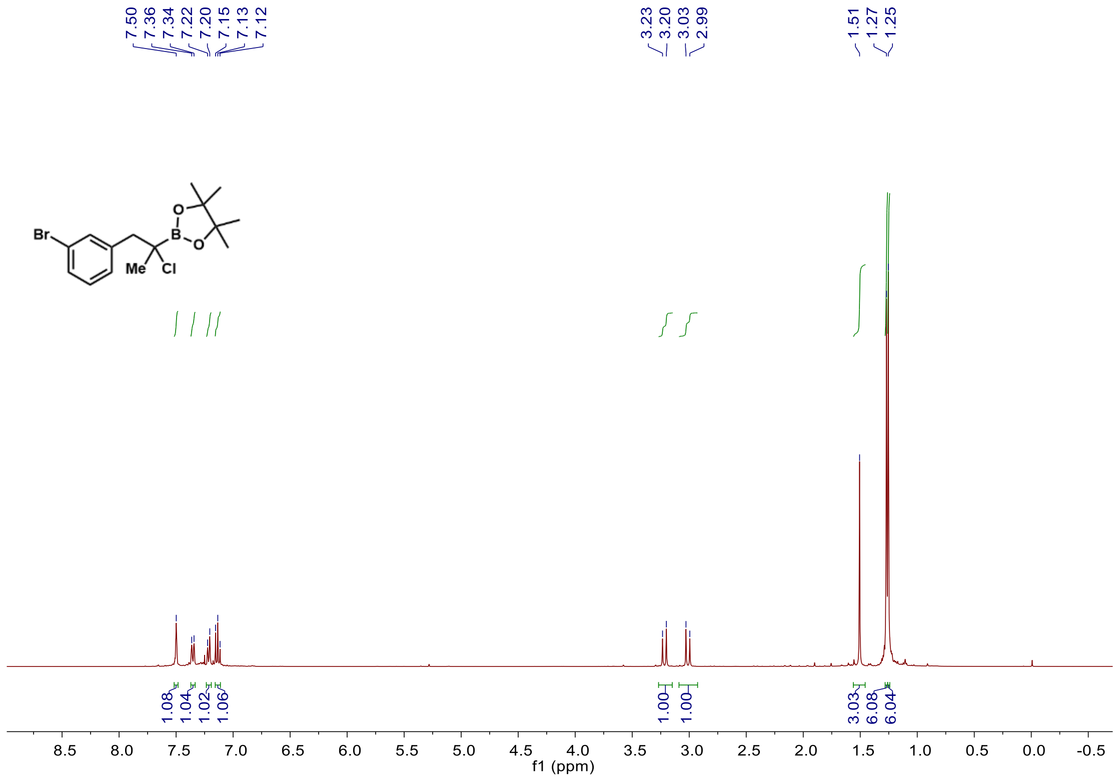

^{1}H NMR spectra (400 MHz, $CDCl_3$) of **17**.

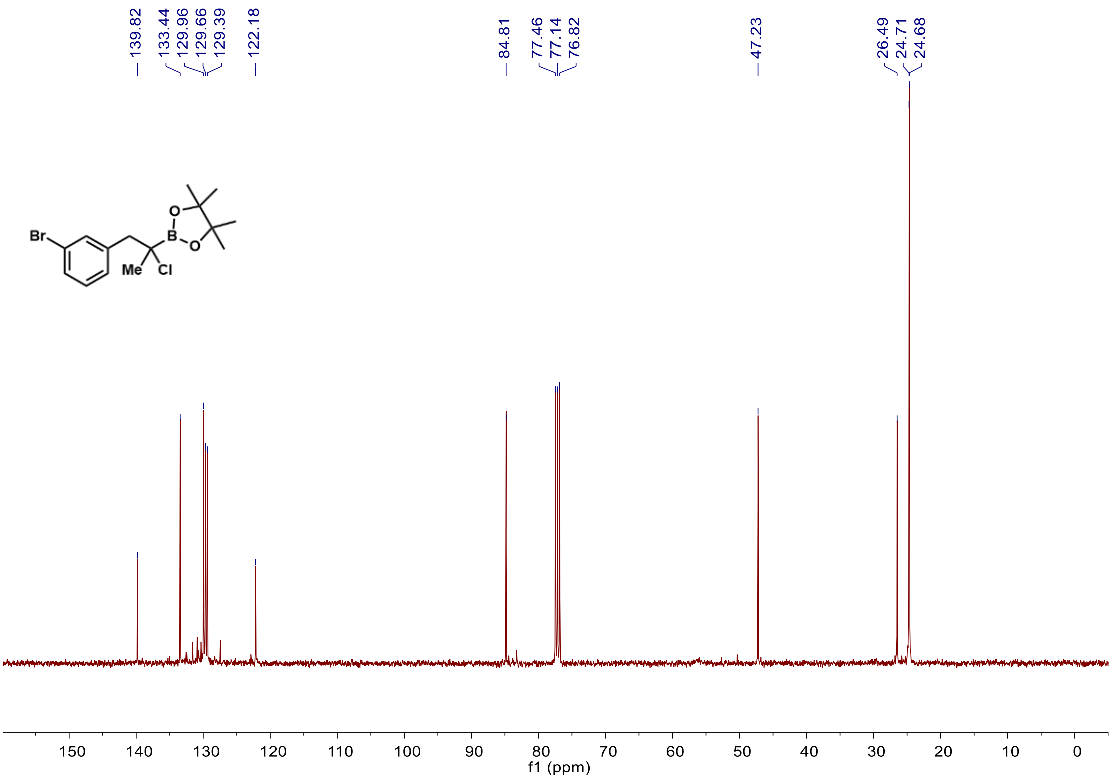

^{13}C NMR spectra (101 MHz, $CDCl_3$) of **17**.

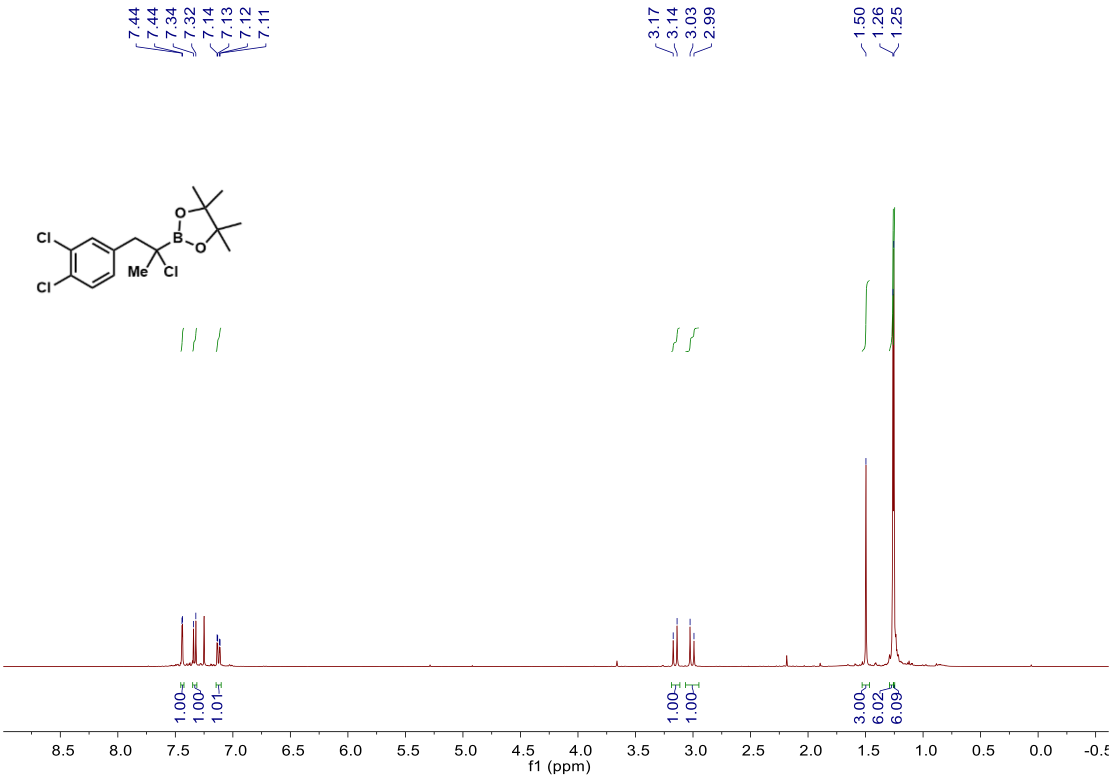

^{1}H NMR spectra (400 MHz, $CDCl_3$) of **18**.

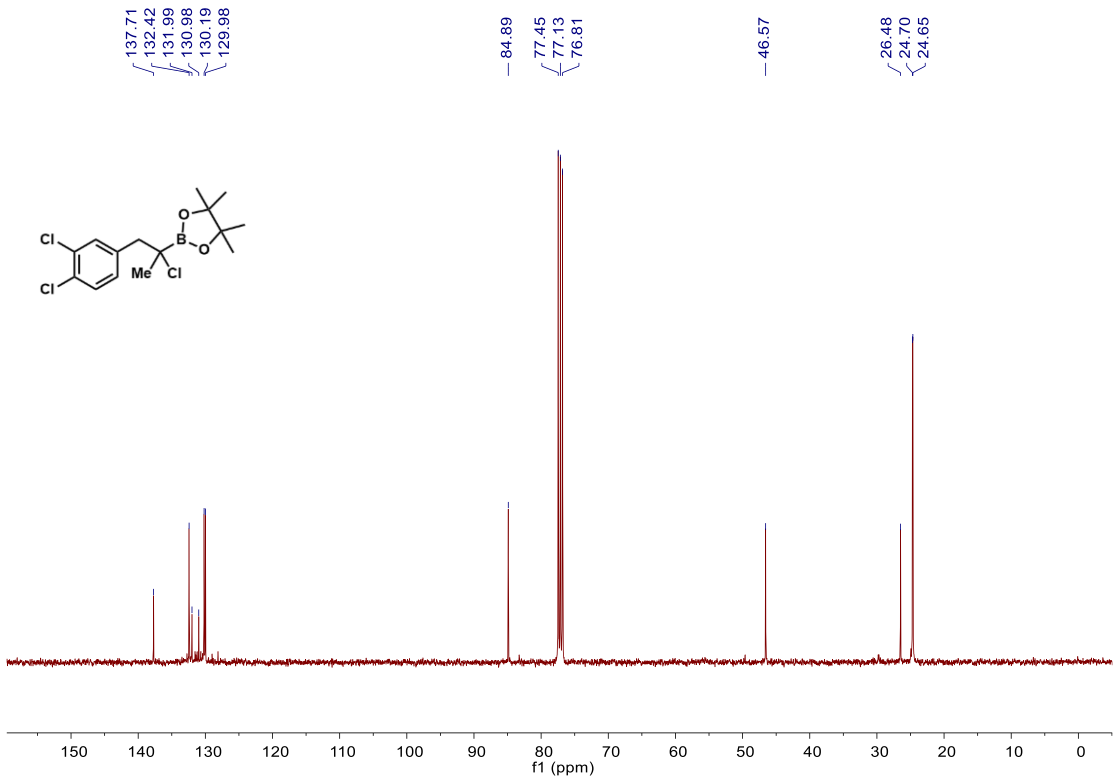

^{13}C NMR spectra (101 MHz, $CDCl_3$) of **18**.

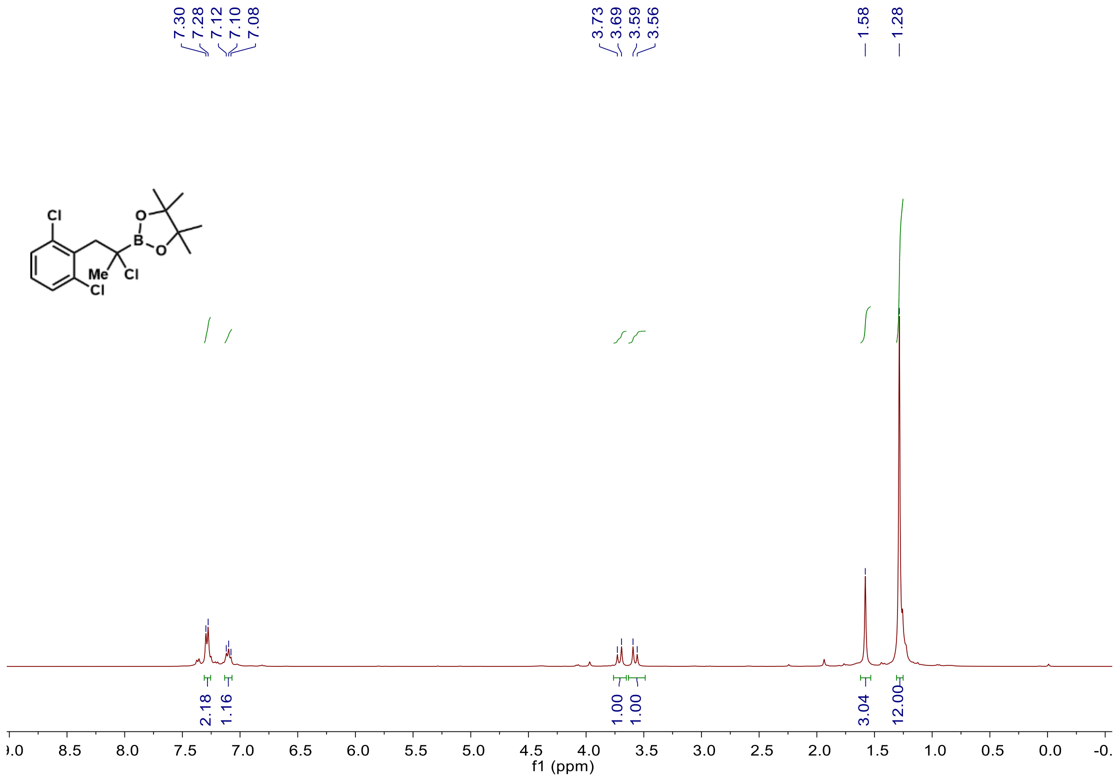

^{1}H NMR spectra (400 MHz, $CDCl_3$) of **19**.

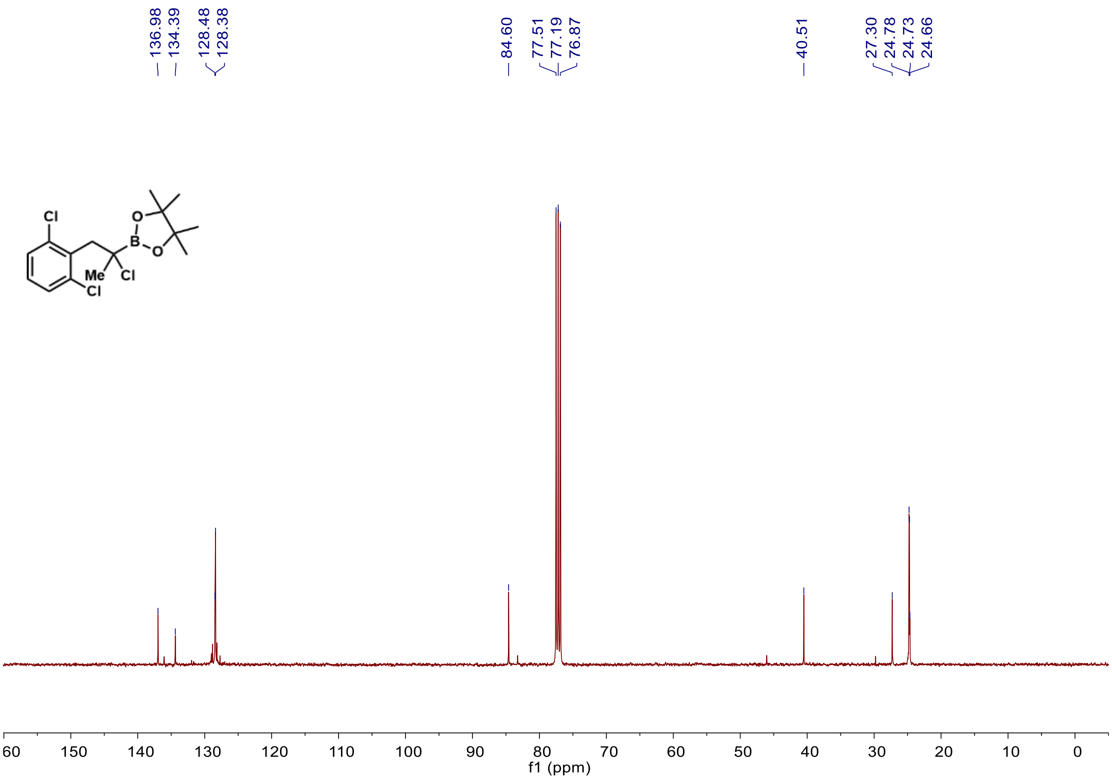

^{13}C NMR spectra (101 MHz, $CDCl_3$) of **19**.

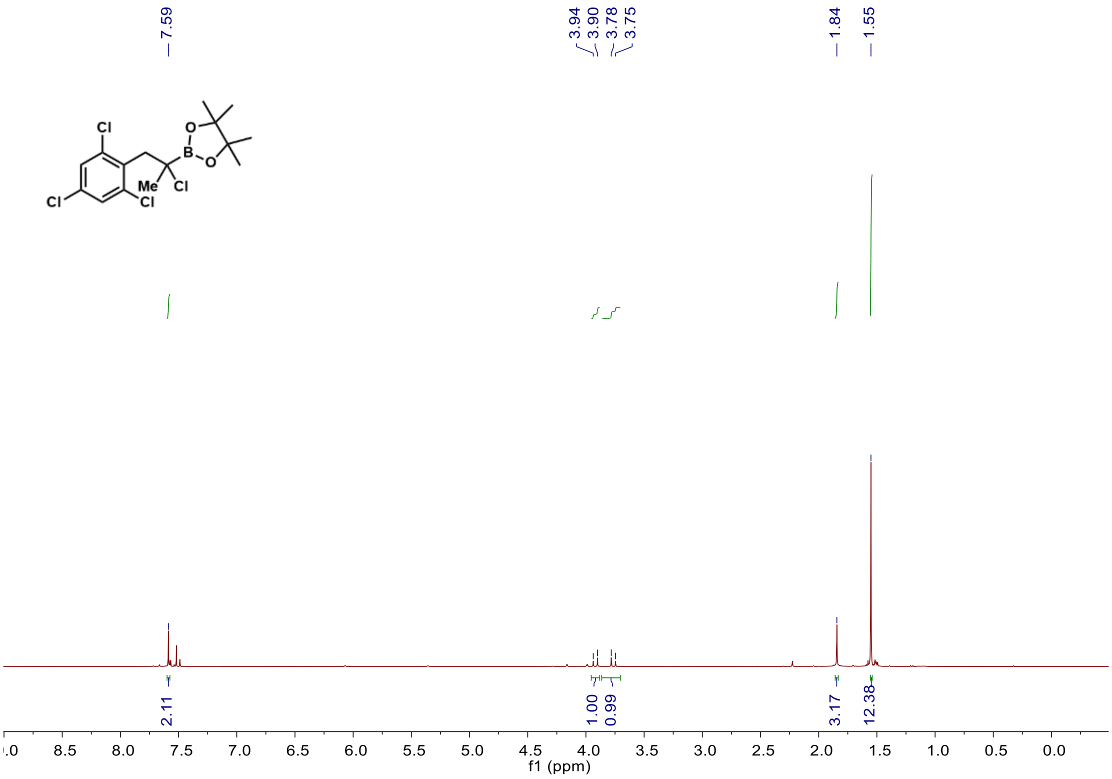

^{1}H NMR spectra (400 MHz, $CDCl_3$) of **20**.

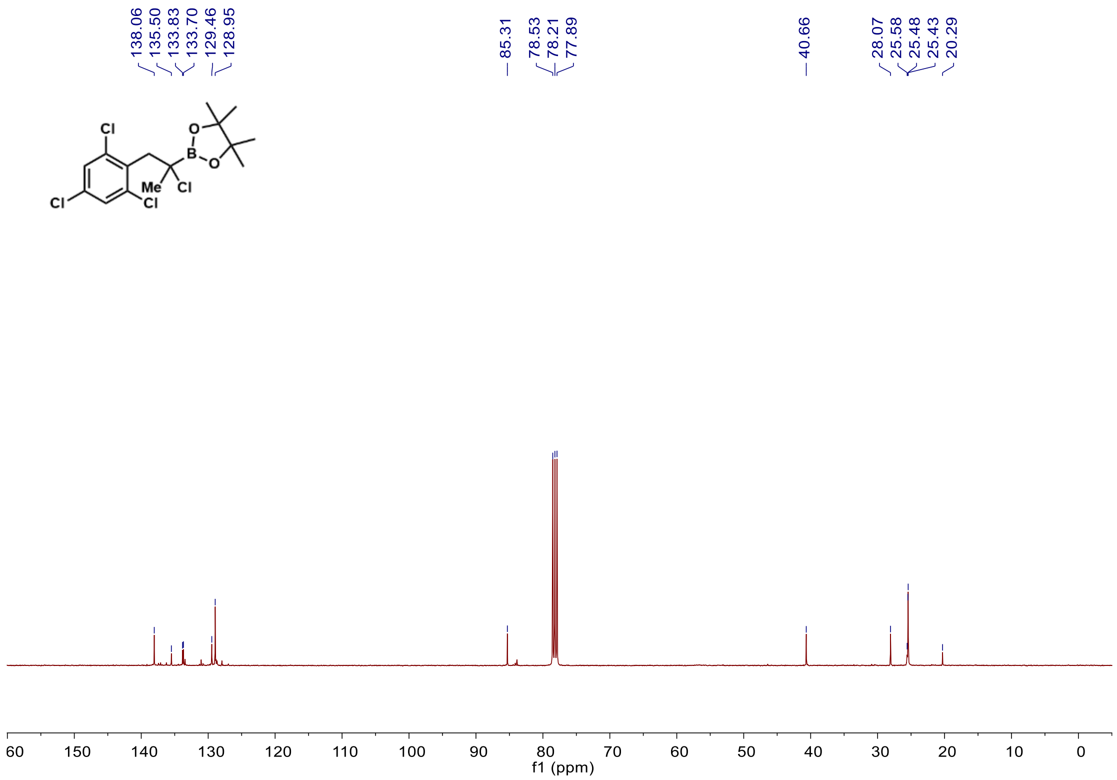

^{13}C NMR spectra (101 MHz, CDCl$_3$) of **20**.

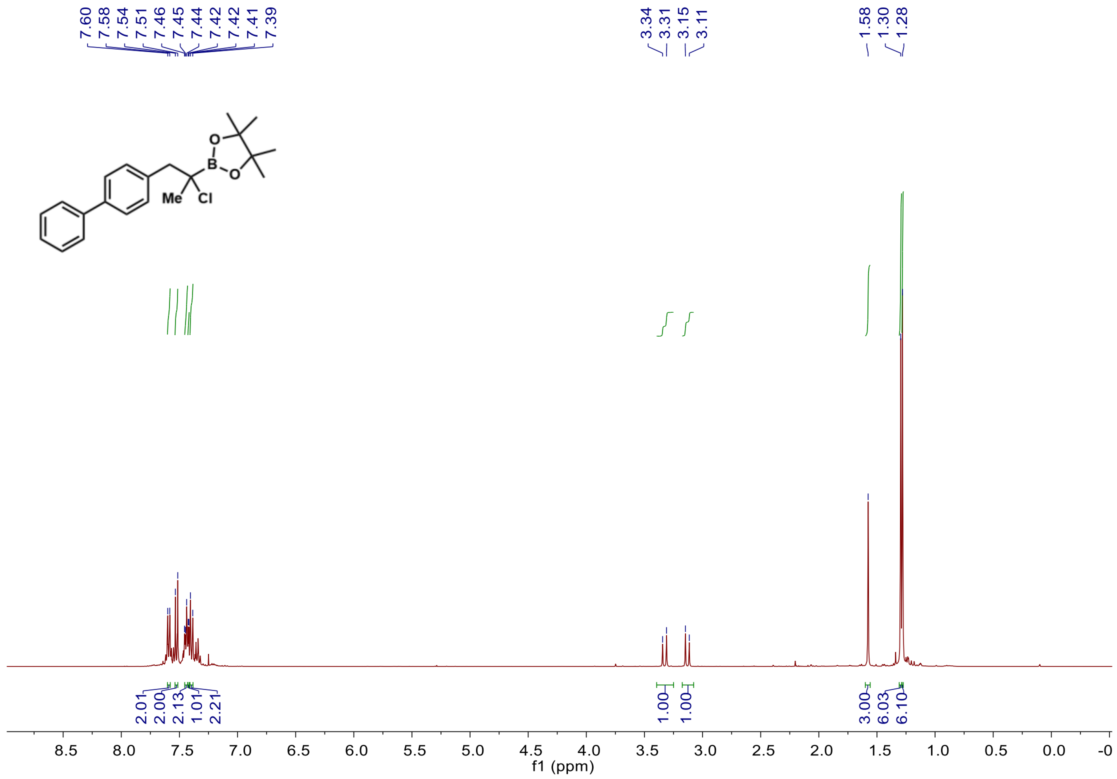

^{1}H NMR spectra (400 MHz, CDCl$_3$) of **21**.

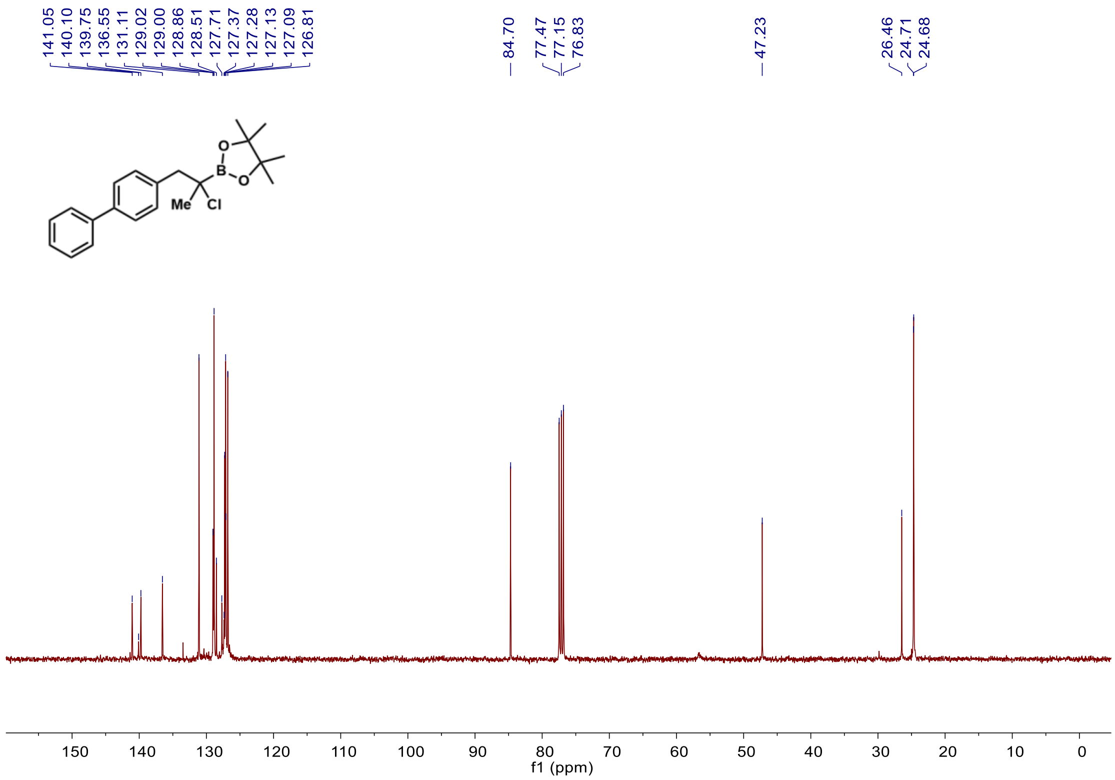

^{13}C NMR spectra (101 MHz, $CDCl_3$) of **21**.

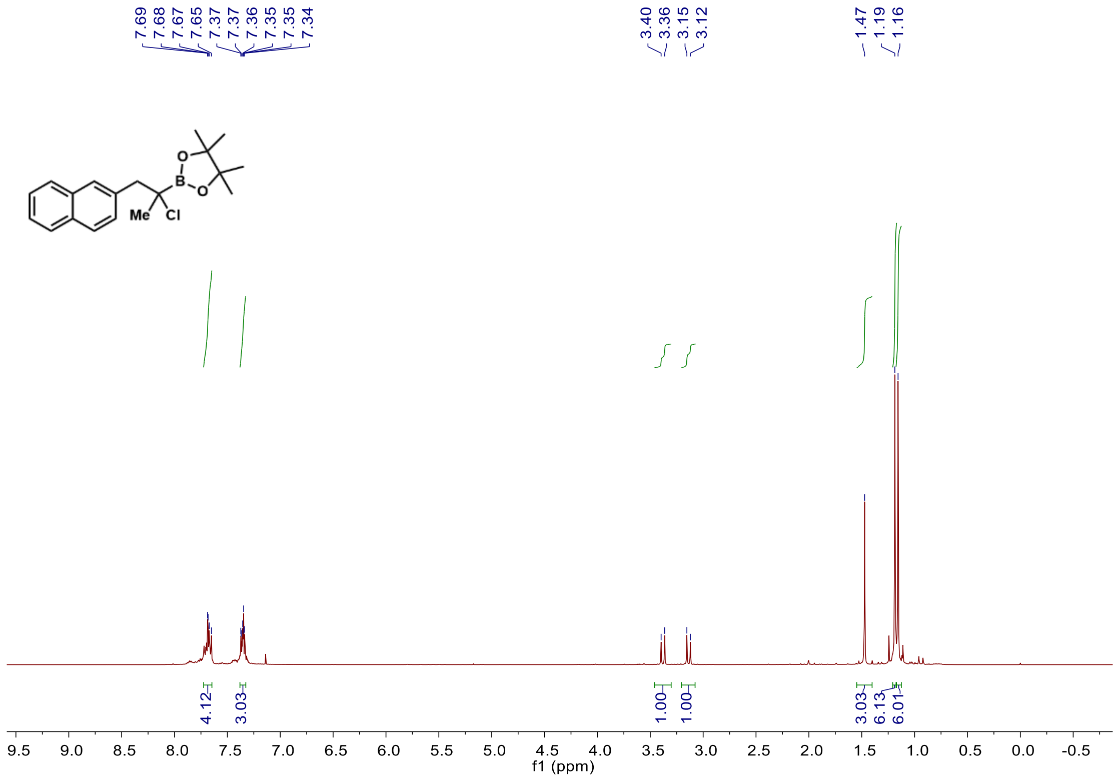

^{1}H NMR spectra (400 MHz, $CDCl_3$) of **22**.

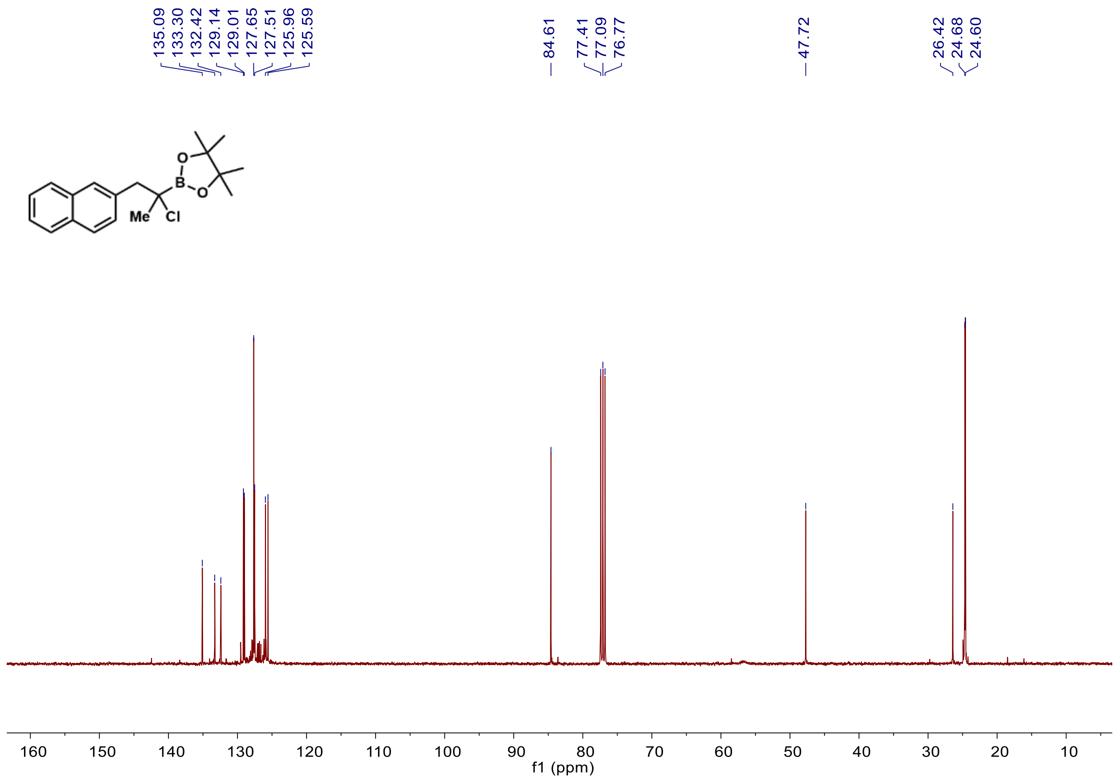

^{13}C NMR spectra (101 MHz, $CDCl_3$) of **22**.

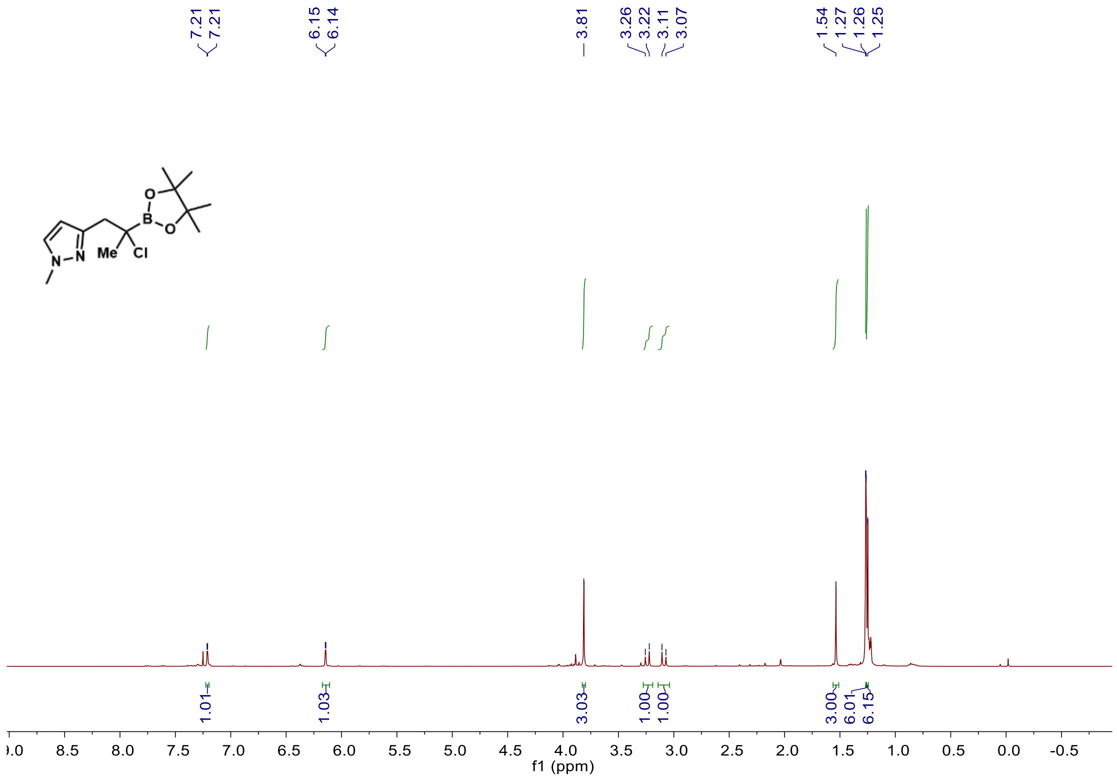

^{1}H NMR spectra (400 MHz, $CDCl_3$) of **23**.

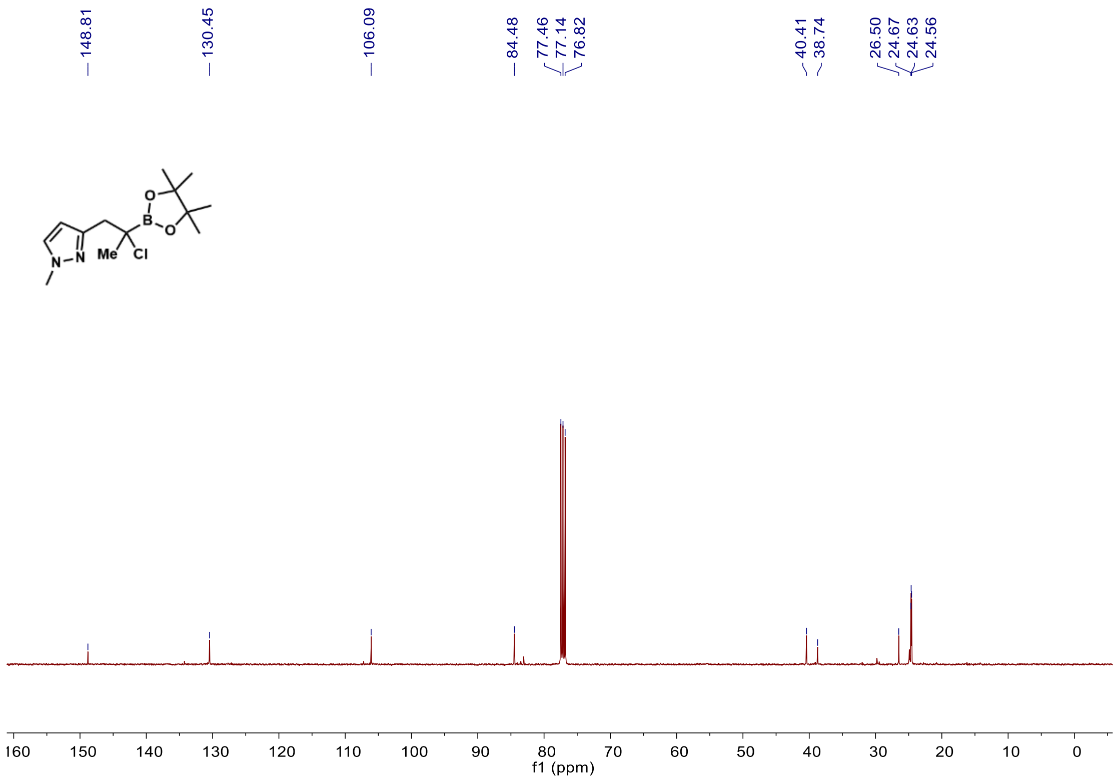

^{13}C NMR spectra (101 MHz, $CDCl_3$) of **23**.

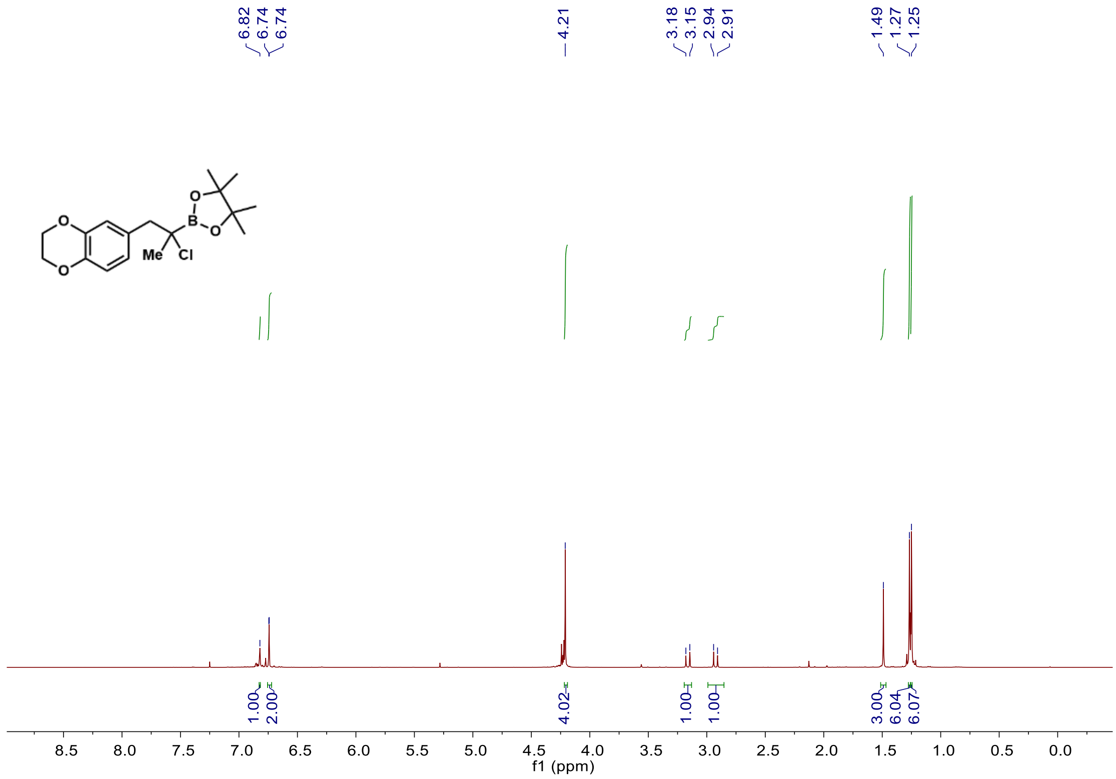

^{1}H NMR spectra (400 MHz, $CDCl_3$) of **24**.

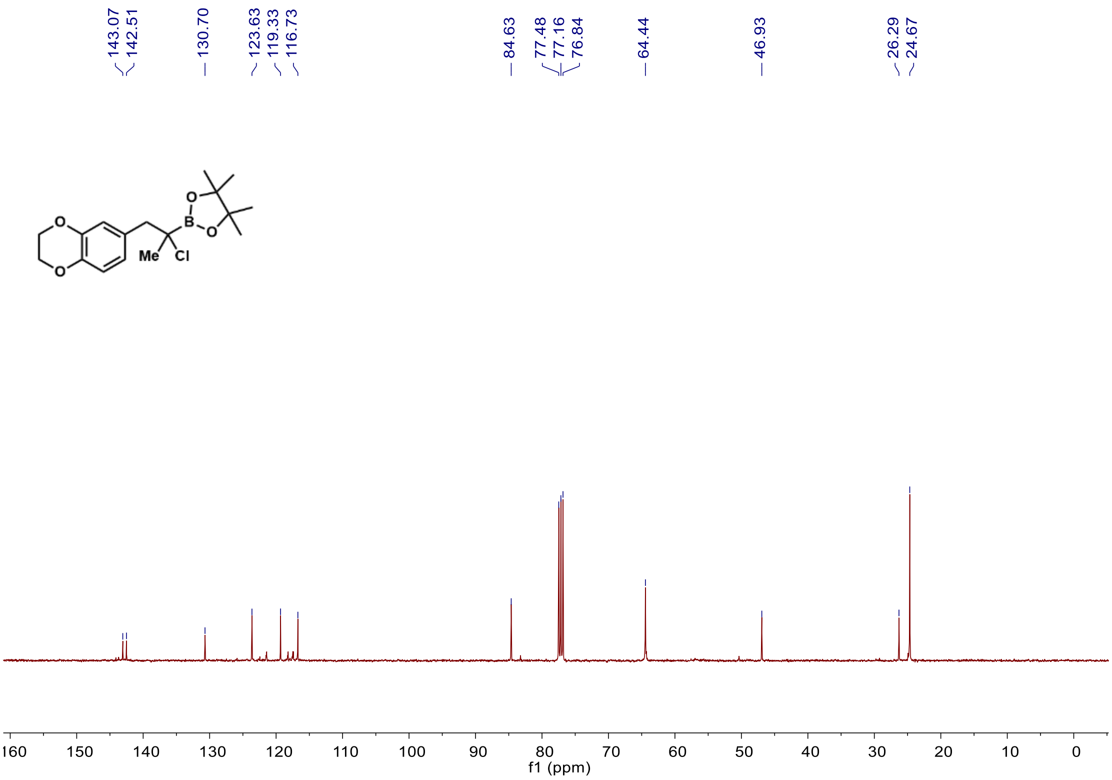

^{13}C NMR spectra (101 MHz, $CDCl_3$) of **24**.

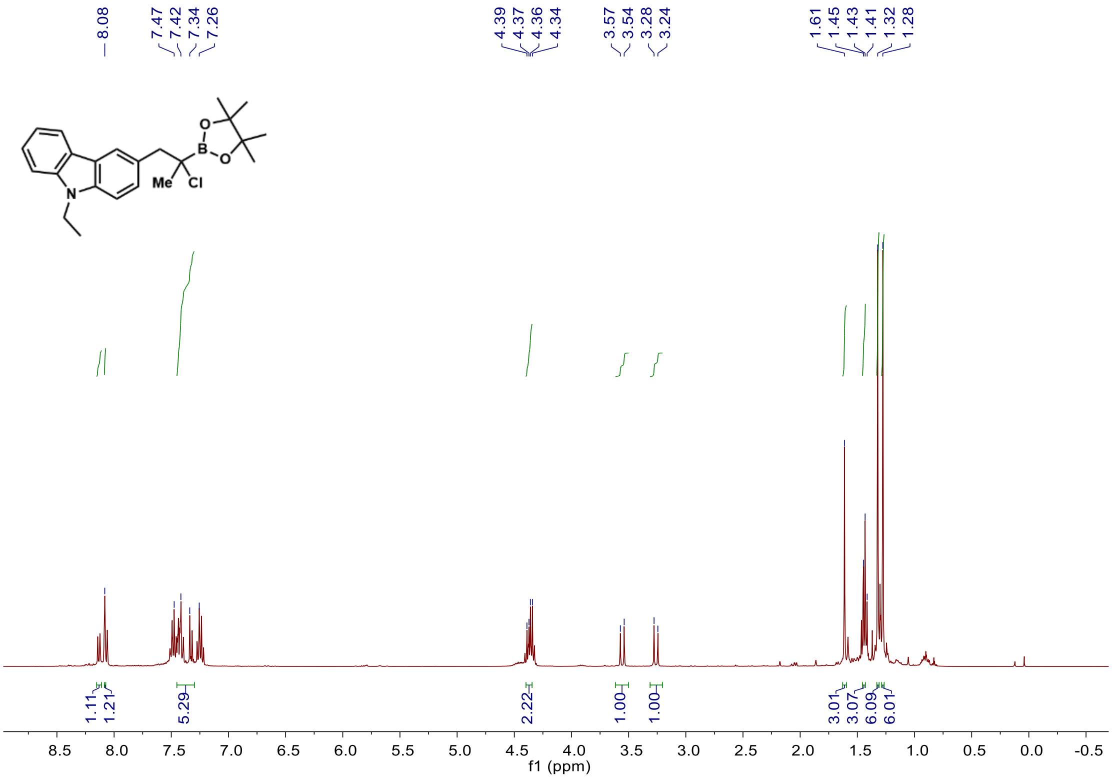

^{1}H NMR spectra (400 MHz, $CDCl_3$) of **25**.

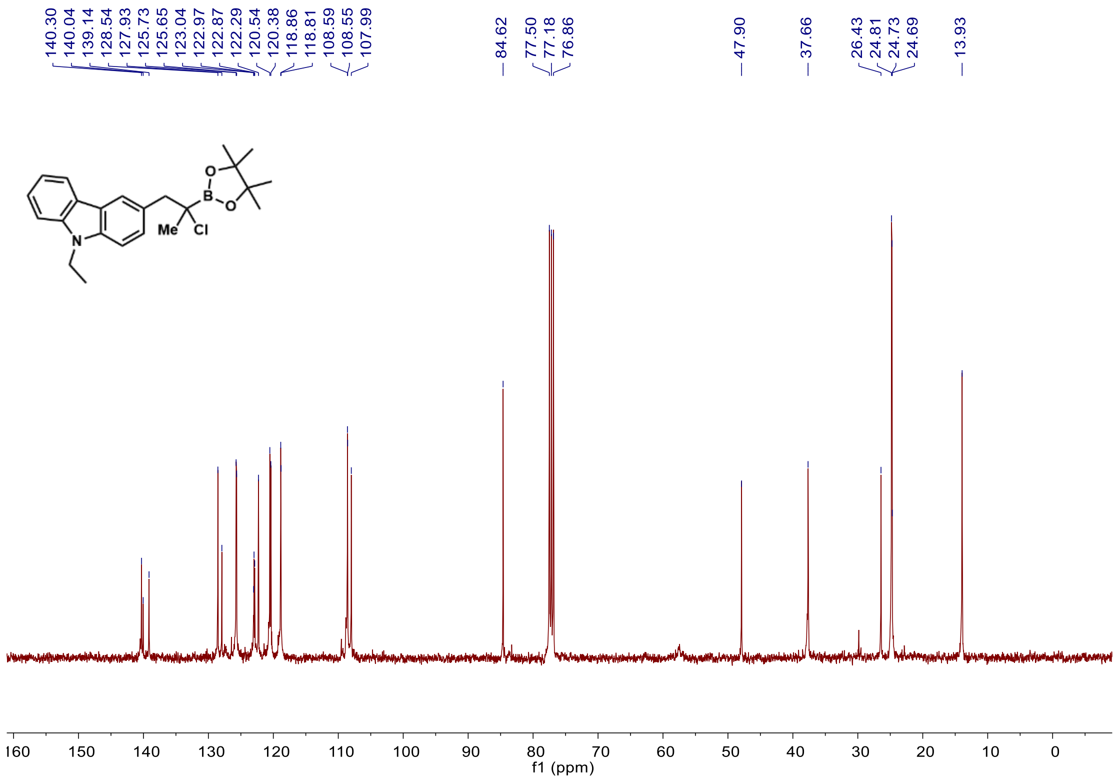

^{13}C NMR spectra (101 MHz, $CDCl_3$) of **25**.

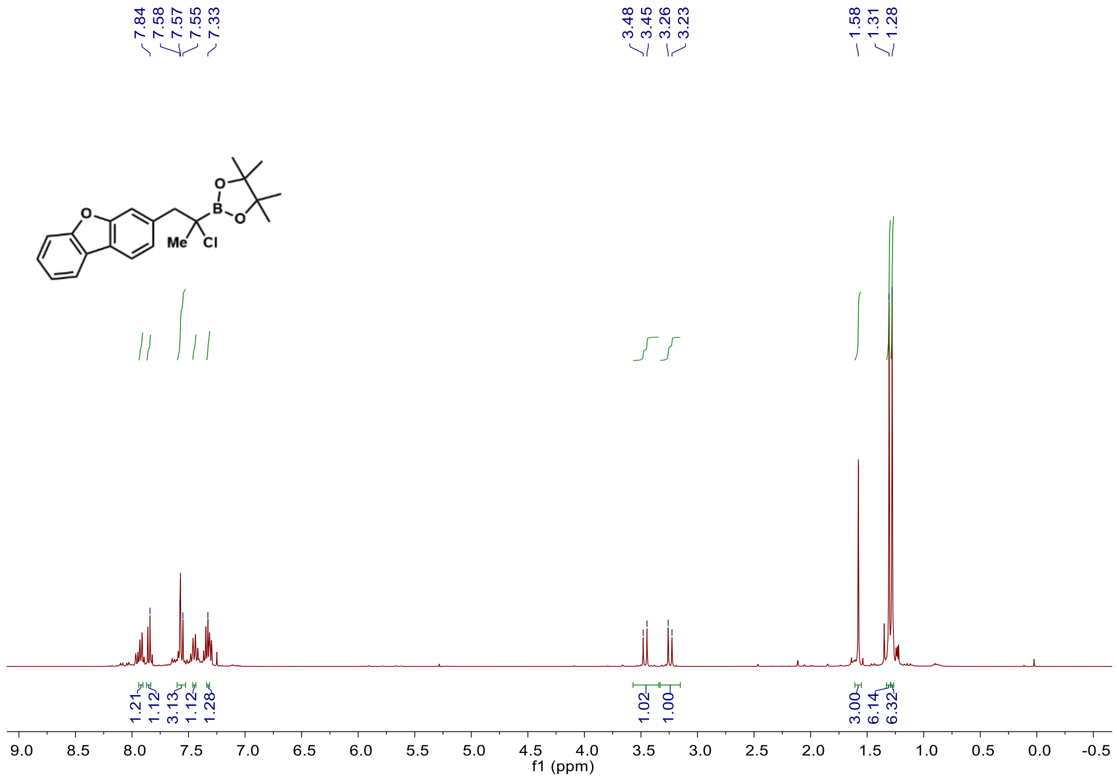

^{1}H NMR spectra (400 MHz, $CDCl_3$) of **26**.

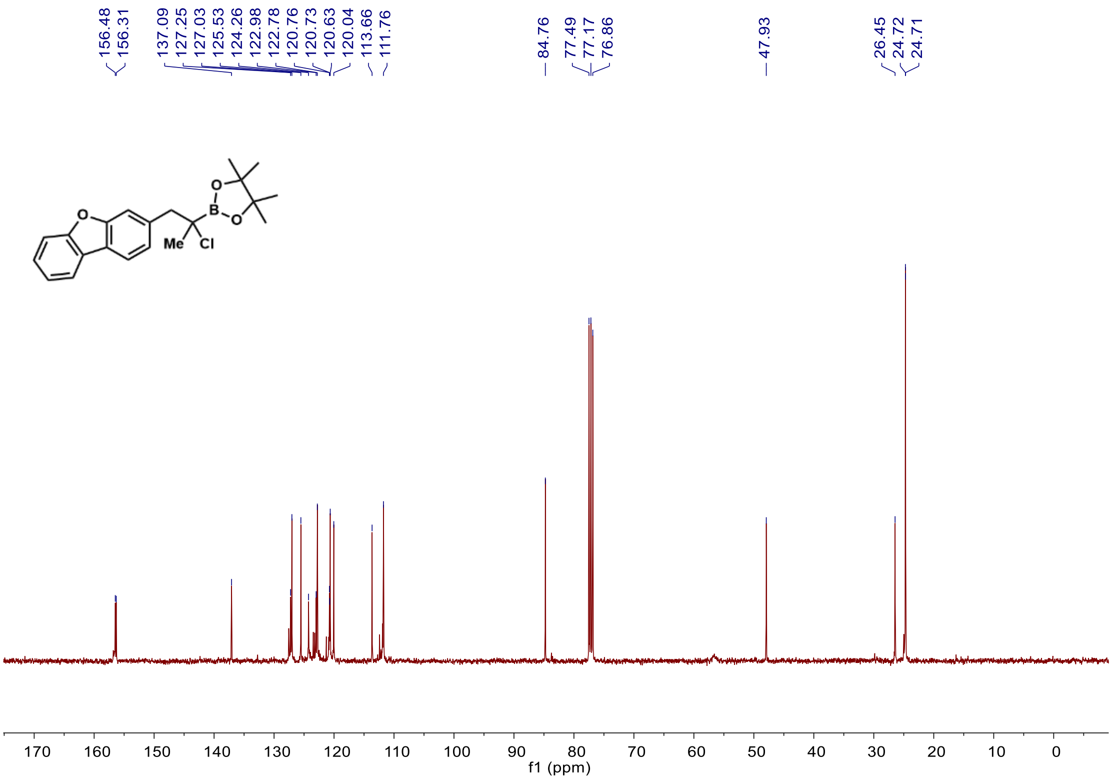

^{13}C NMR spectra (101 MHz, $CDCl_3$) of **26**.

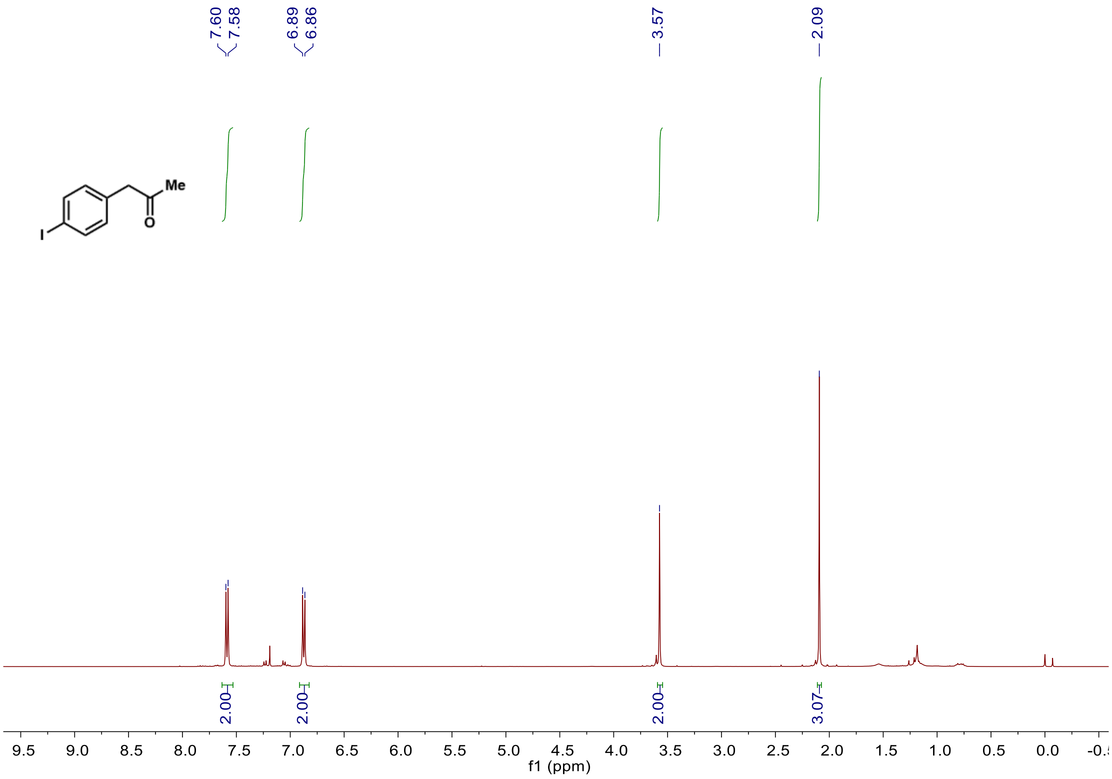

^{1}H NMR spectra (400 MHz, $CDCl_3$) of **27**.

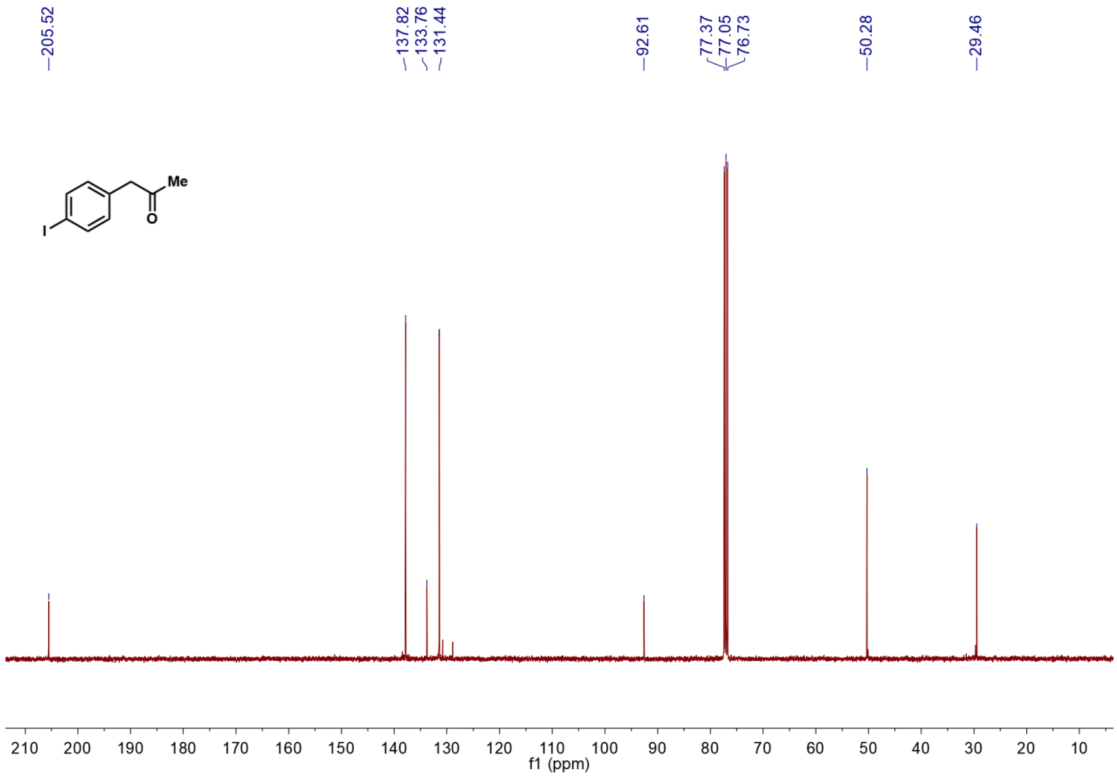

^{13}C NMR spectra (101 MHz, CDCl$_3$) of **27**.

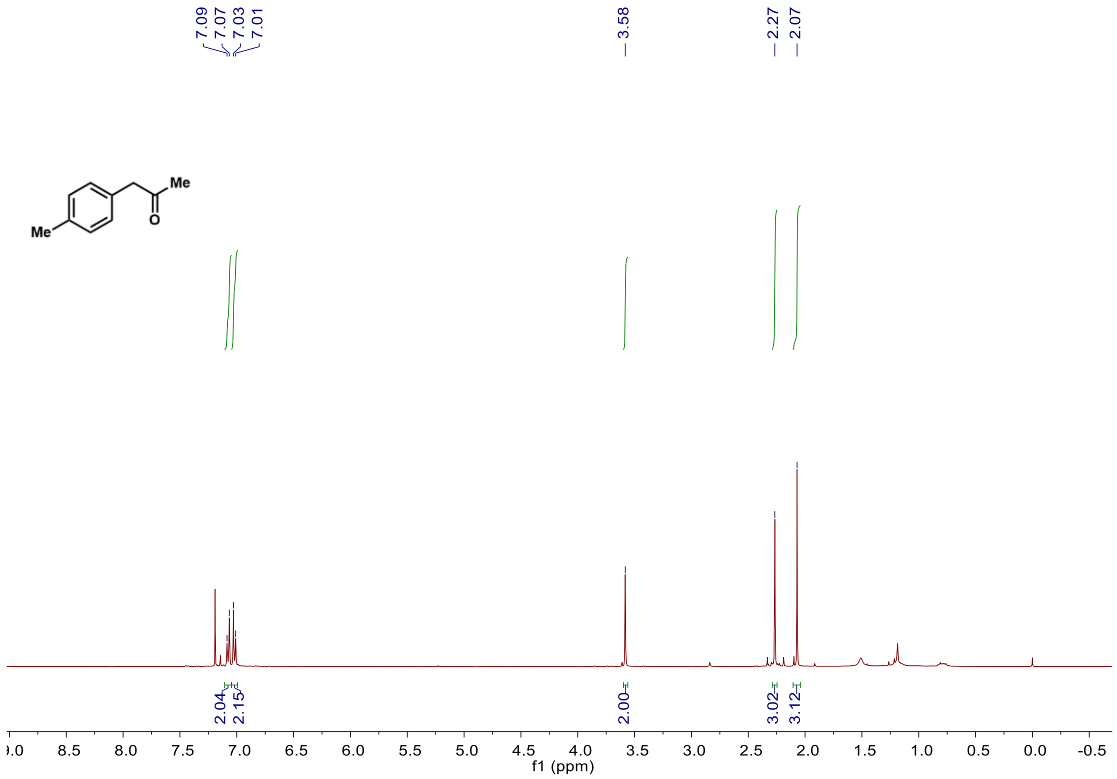

^{1}H NMR spectra (400 MHz, CDCl$_3$) of **28**.

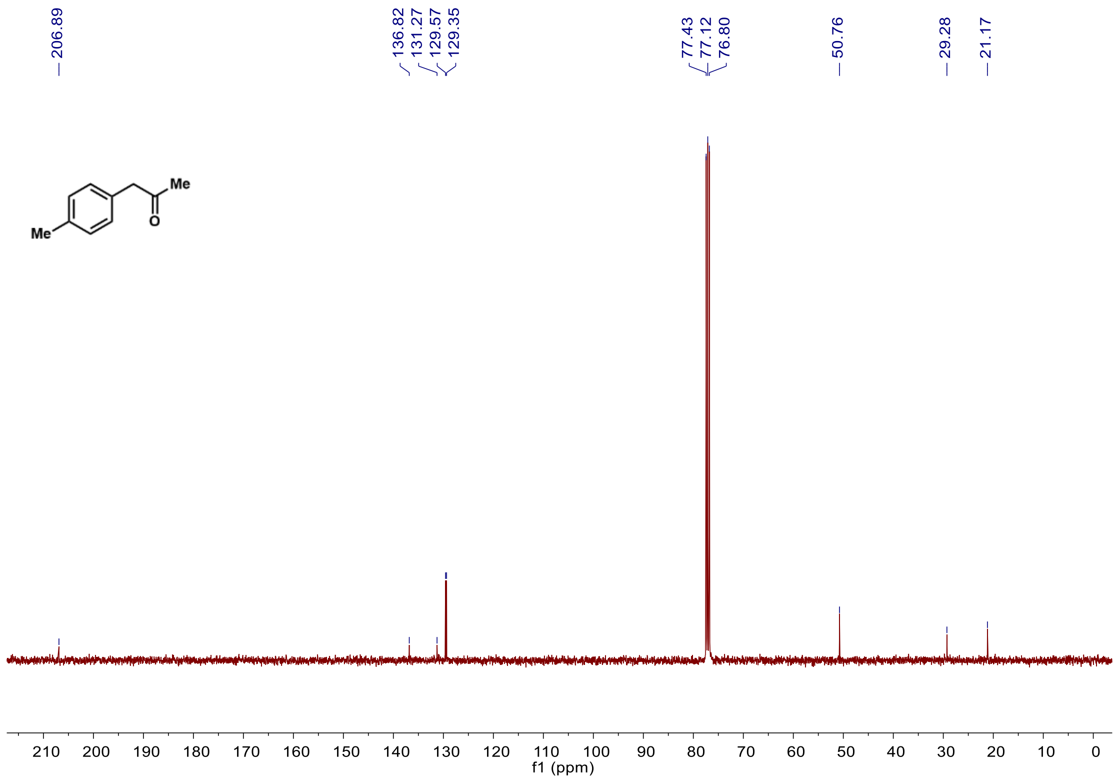

^{13}C NMR spectra (101 MHz, $CDCl_3$) of **28**.

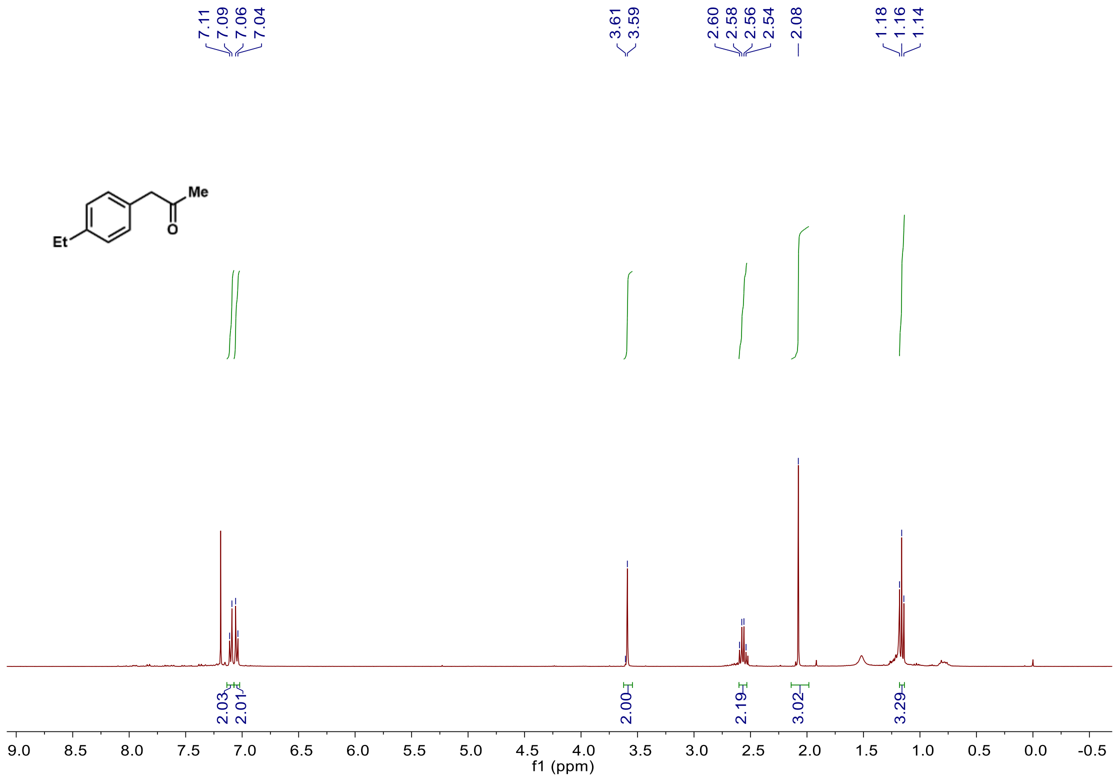

^{1}H NMR spectra (400 MHz, $CDCl_3$) of **29**.

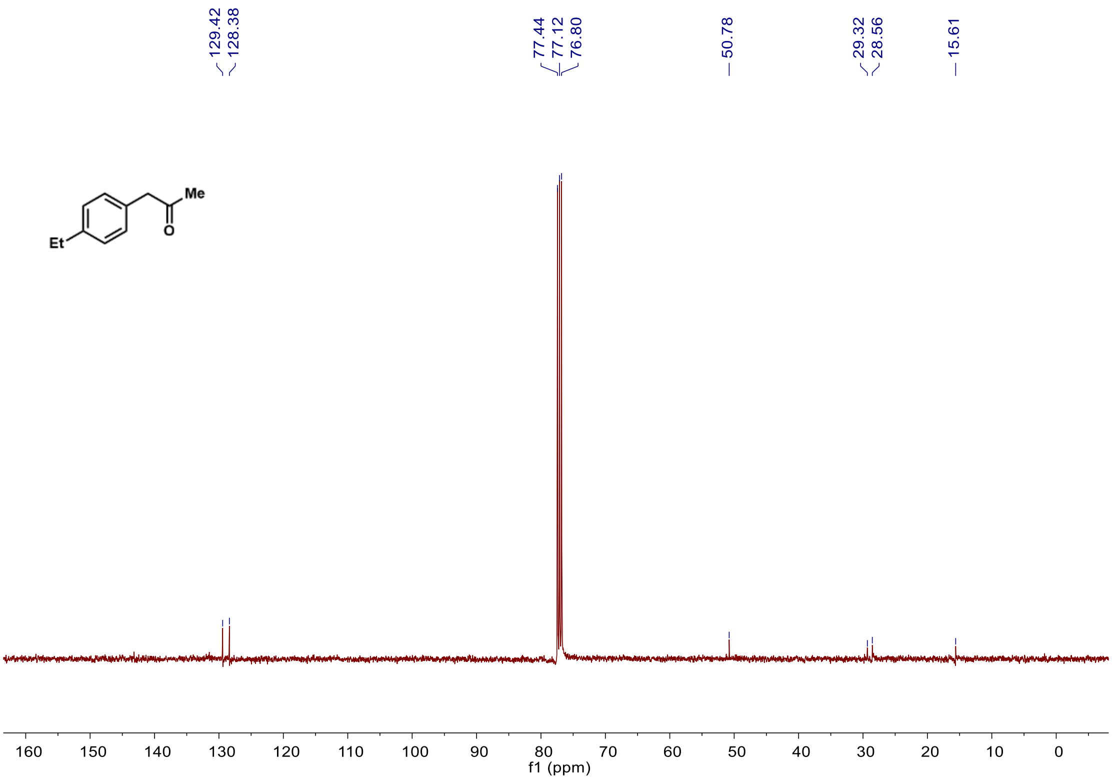

^{13}C NMR spectra (101 MHz, $CDCl_3$) of **29**.

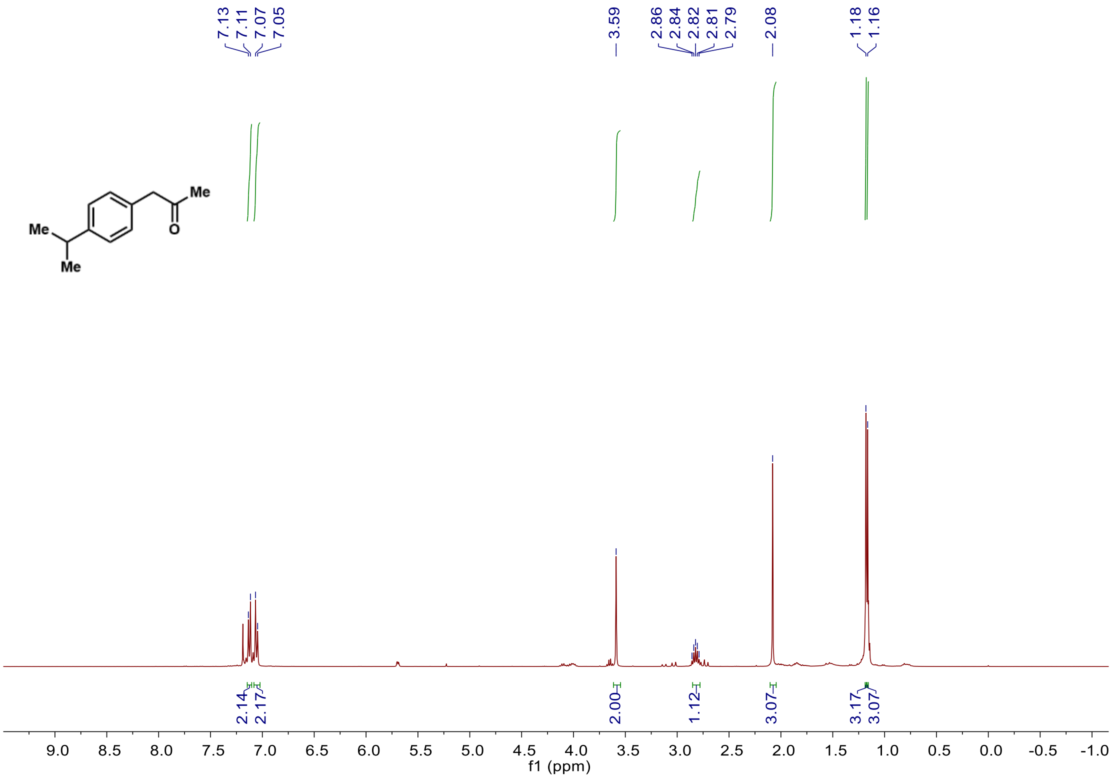

^{1}H NMR spectra (400 MHz, $CDCl_3$) of **30**.

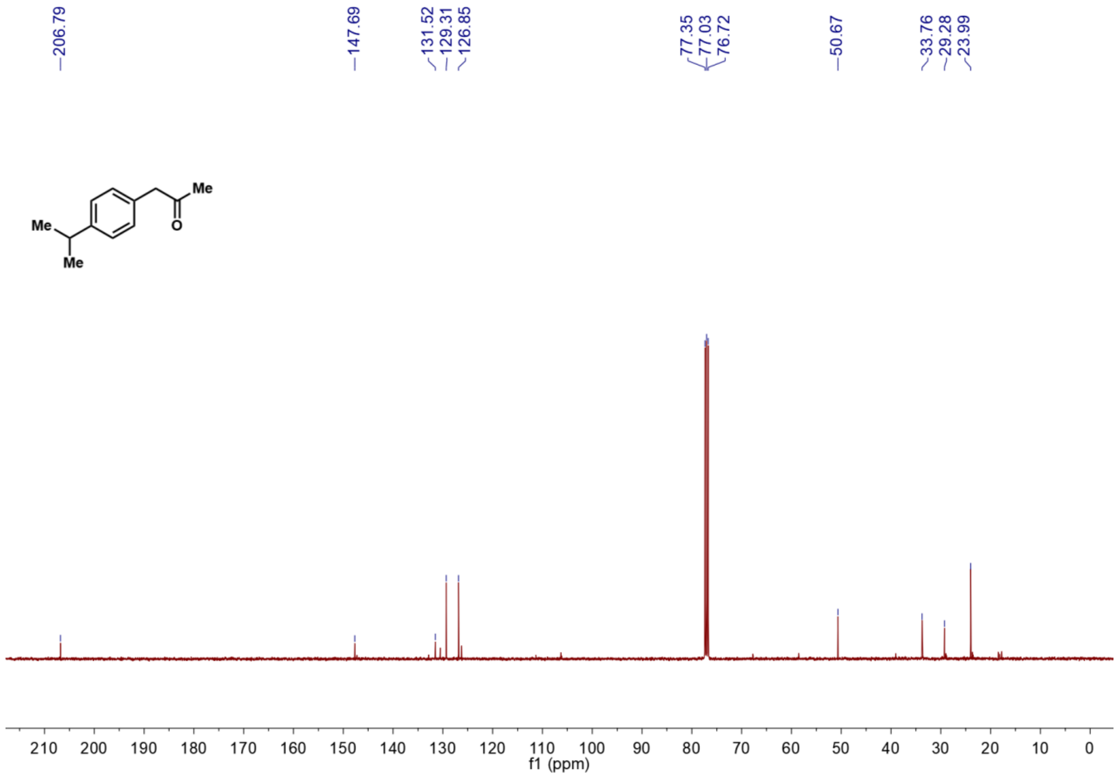

^{13}C NMR spectra (101 MHz, $CDCl_3$) of **30**.

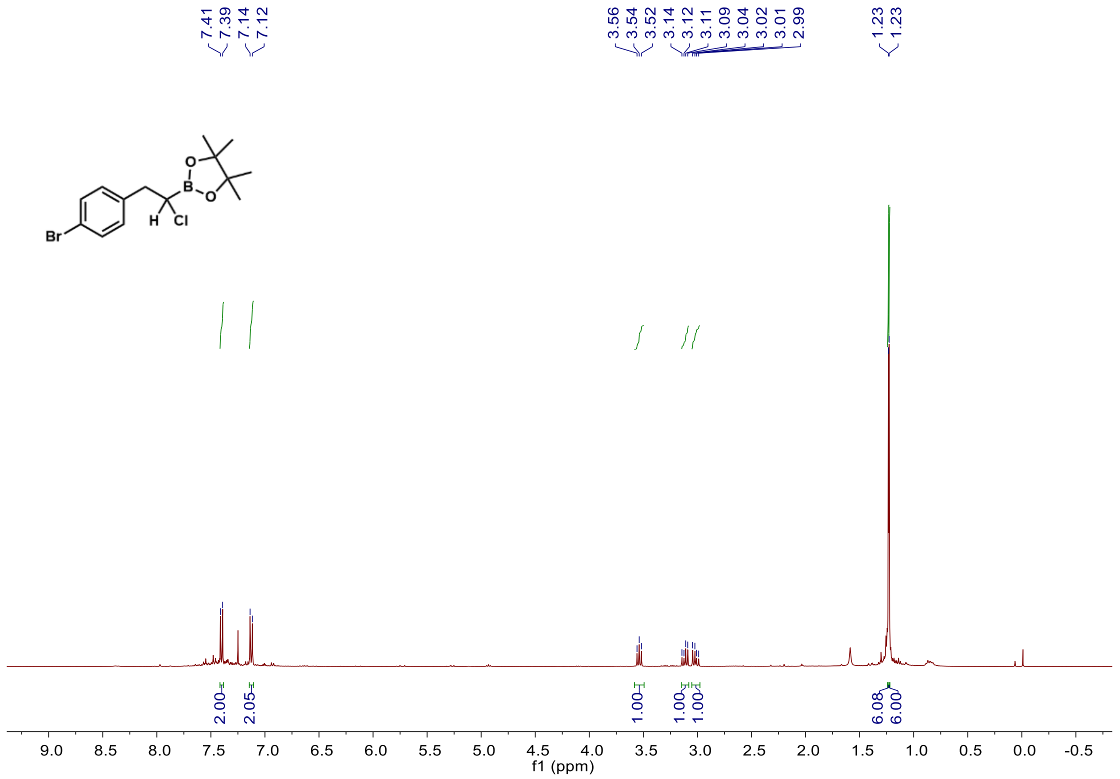

^{1}H NMR spectra (400 MHz, $CDCl_3$) of **31**.

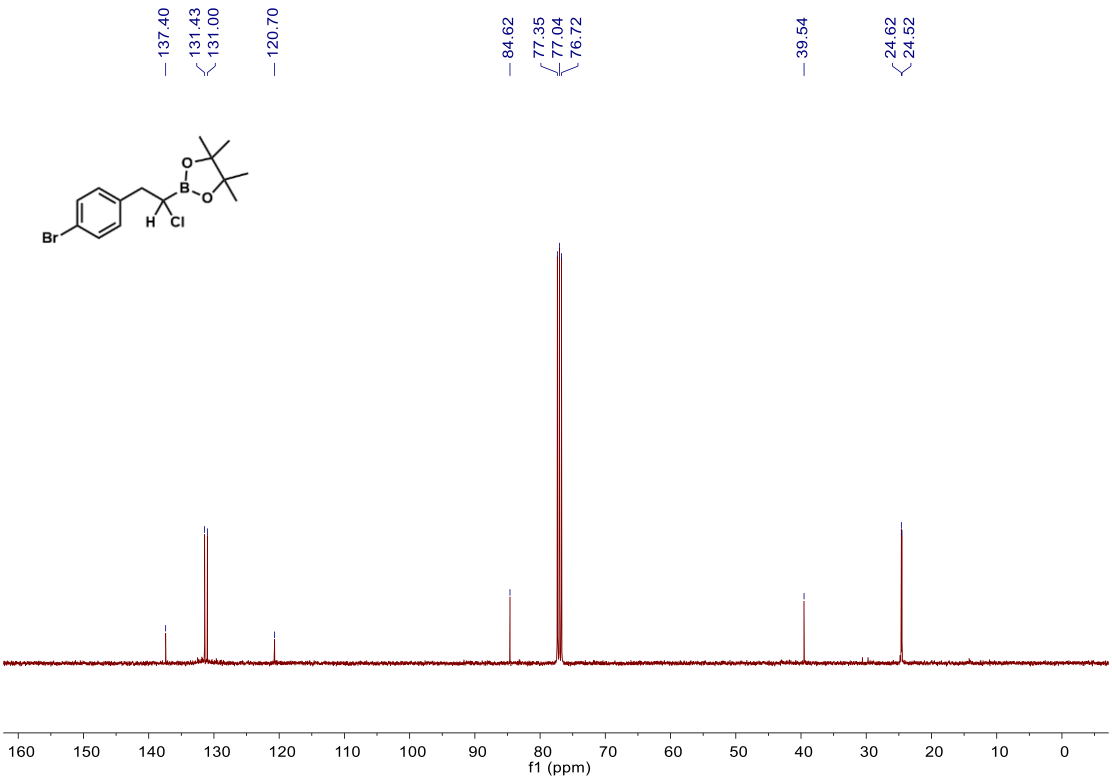

^{13}C NMR spectra (101 MHz, $CDCl_3$) of **31**.

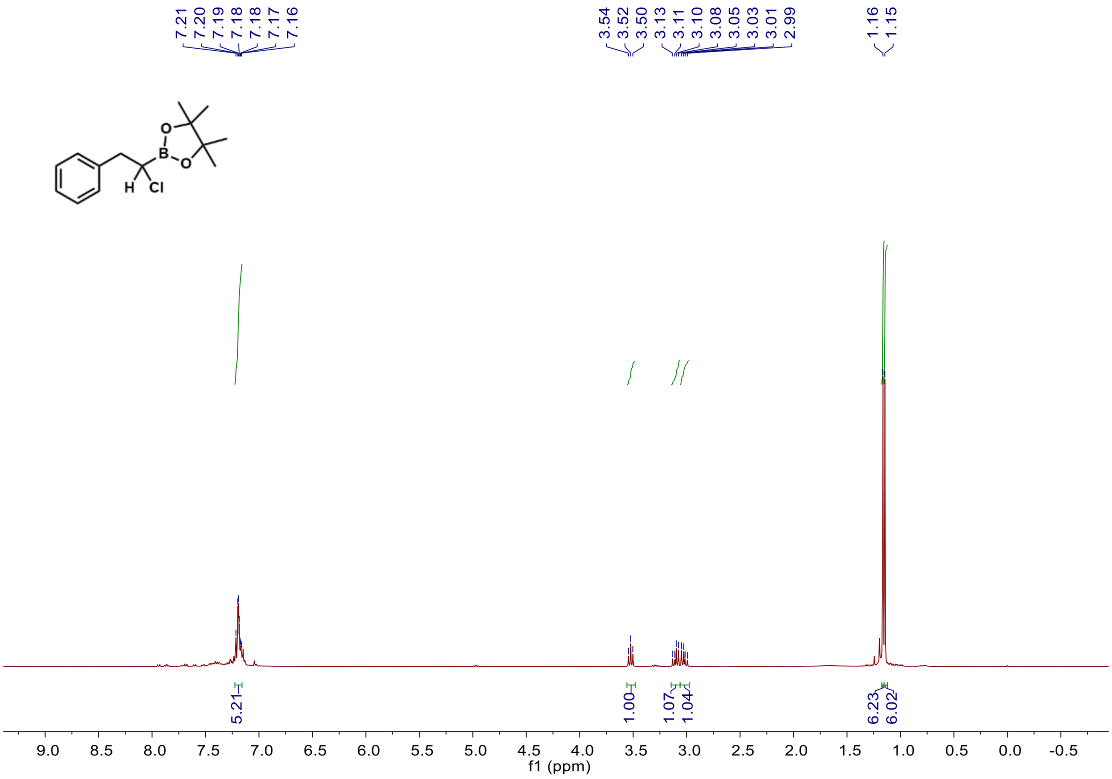

^{1}H NMR spectra (400 MHz, $CDCl_3$) of **32**.

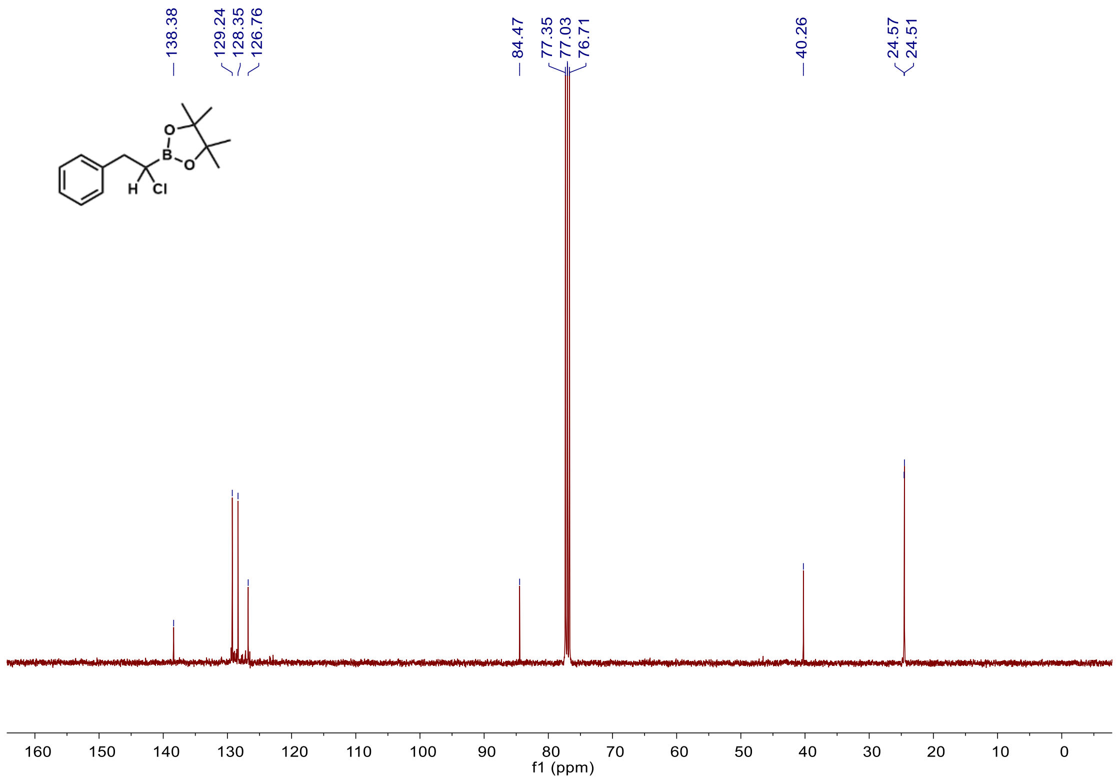

^{13}C NMR spectra (101 MHz, $CDCl_3$) of **32**.

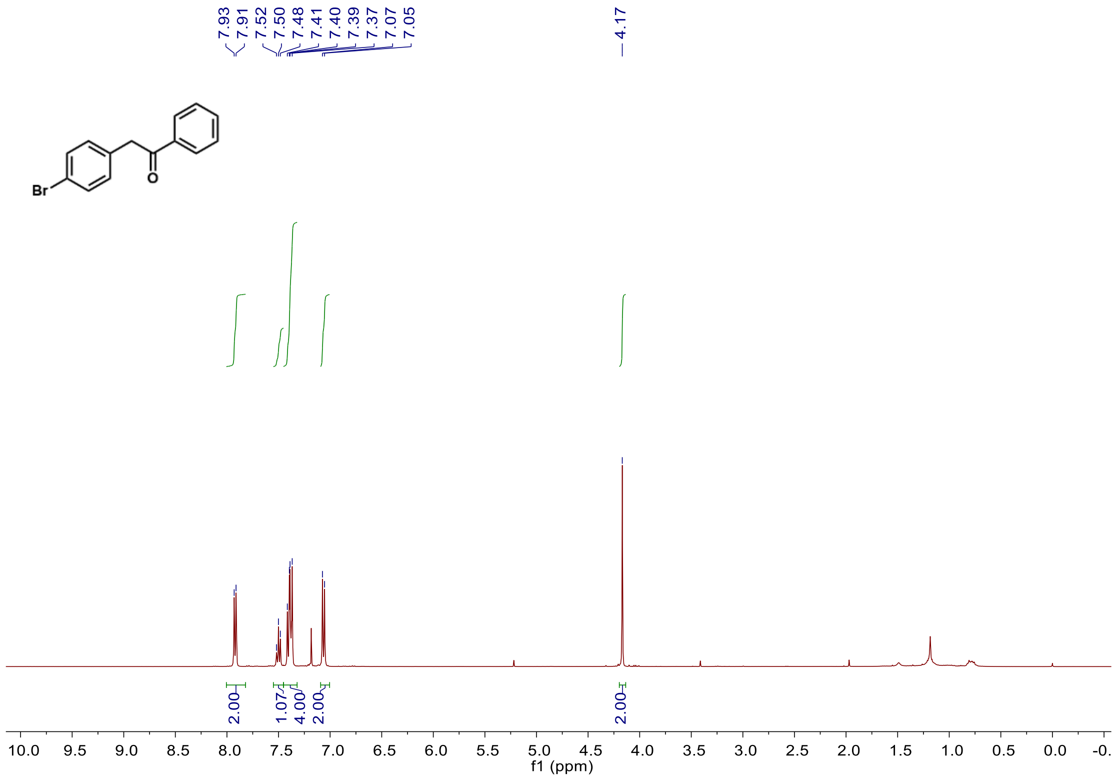

^{1}H NMR spectra (400 MHz, $CDCl_3$) of **33’**.

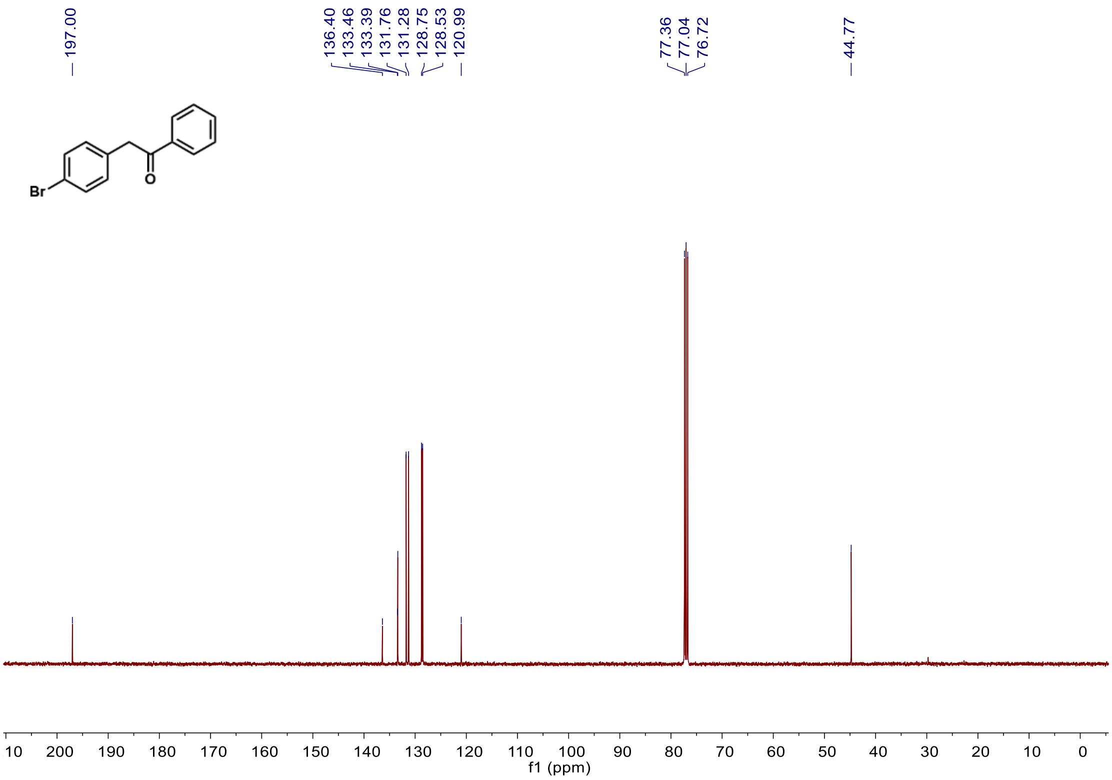

^{13}C NMR spectra (101 MHz, $CDCl_3$) of **33'**.

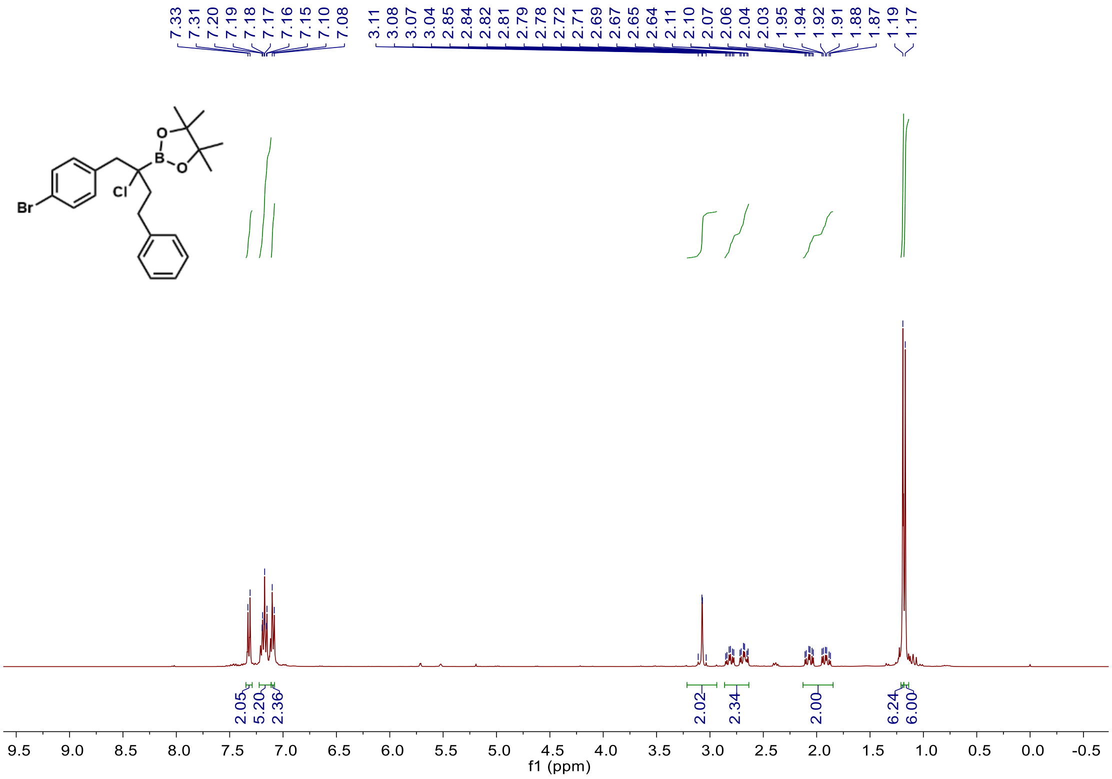

^{1}H NMR spectra (400 MHz, $CDCl_3$) of **34**.

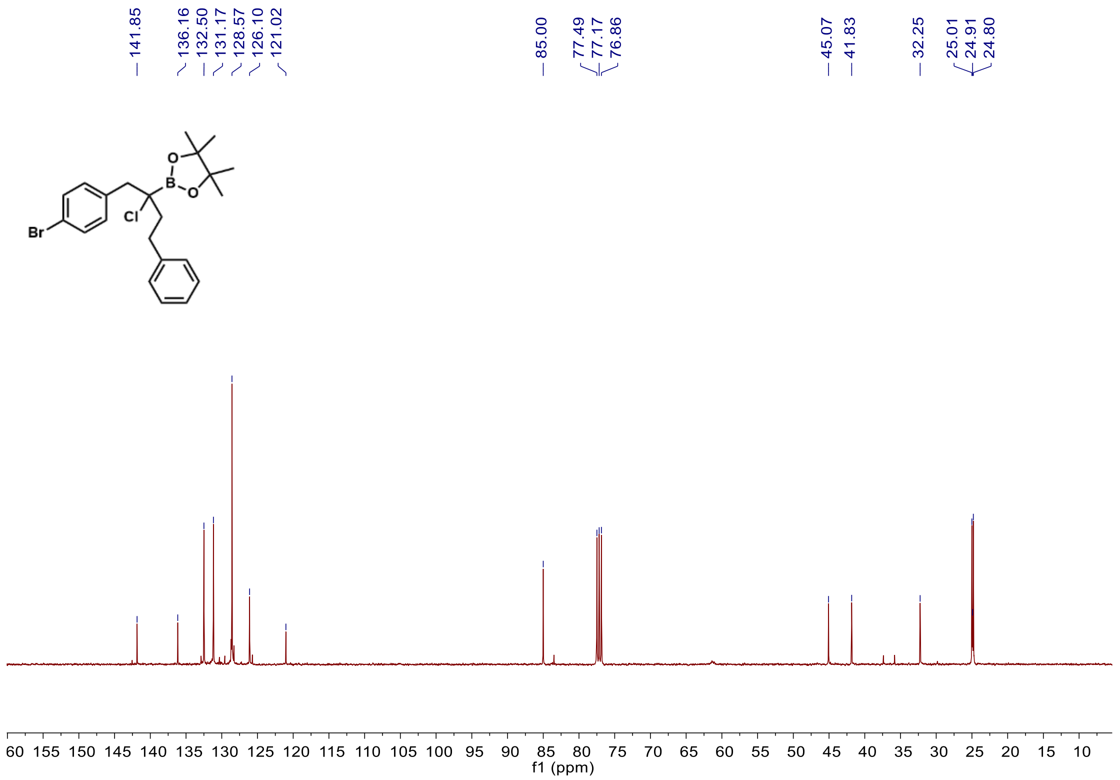

^{13}C NMR spectra (101 MHz, $CDCl_3$) of **34**.

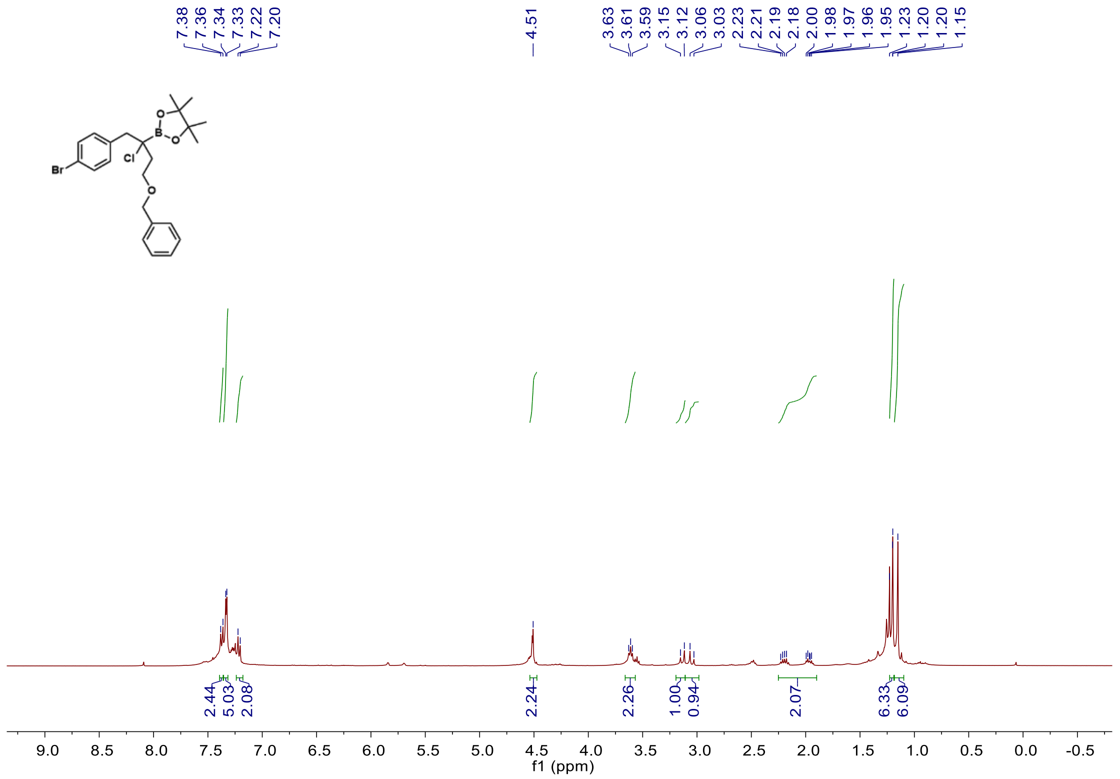

^{1}H NMR spectra (400 MHz, $CDCl_3$) of **35**.

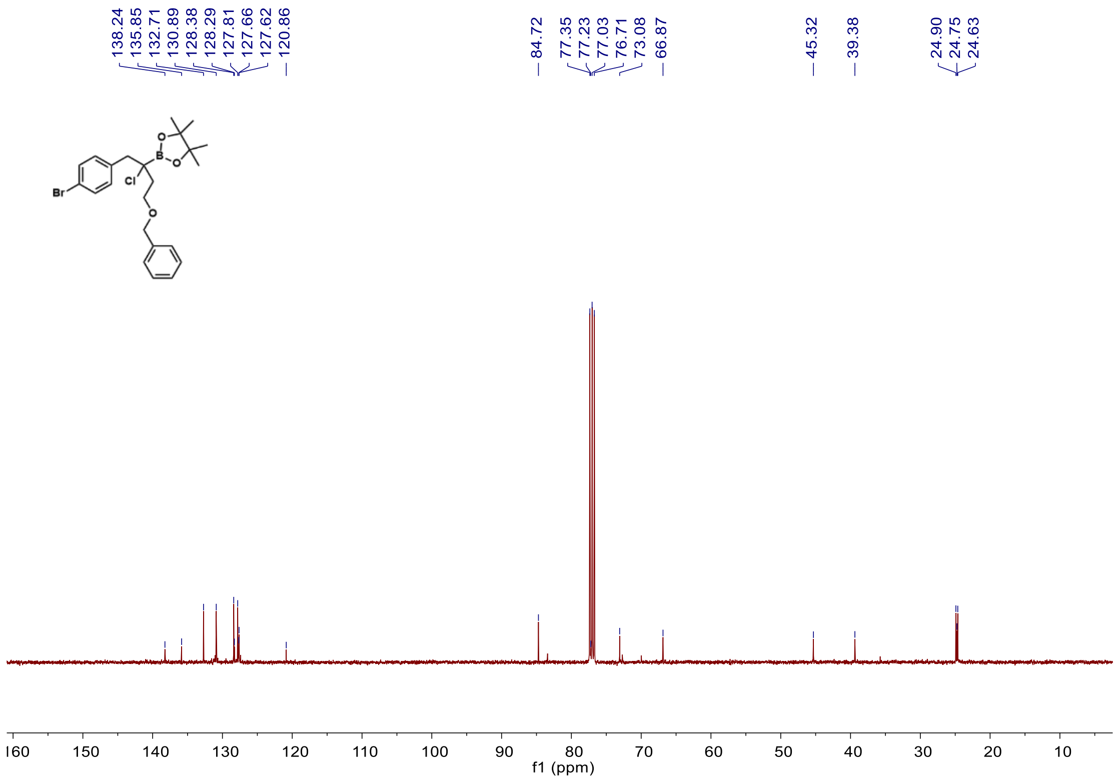

^{13}C NMR spectra (101 MHz, $CDCl_3$) of **35**.

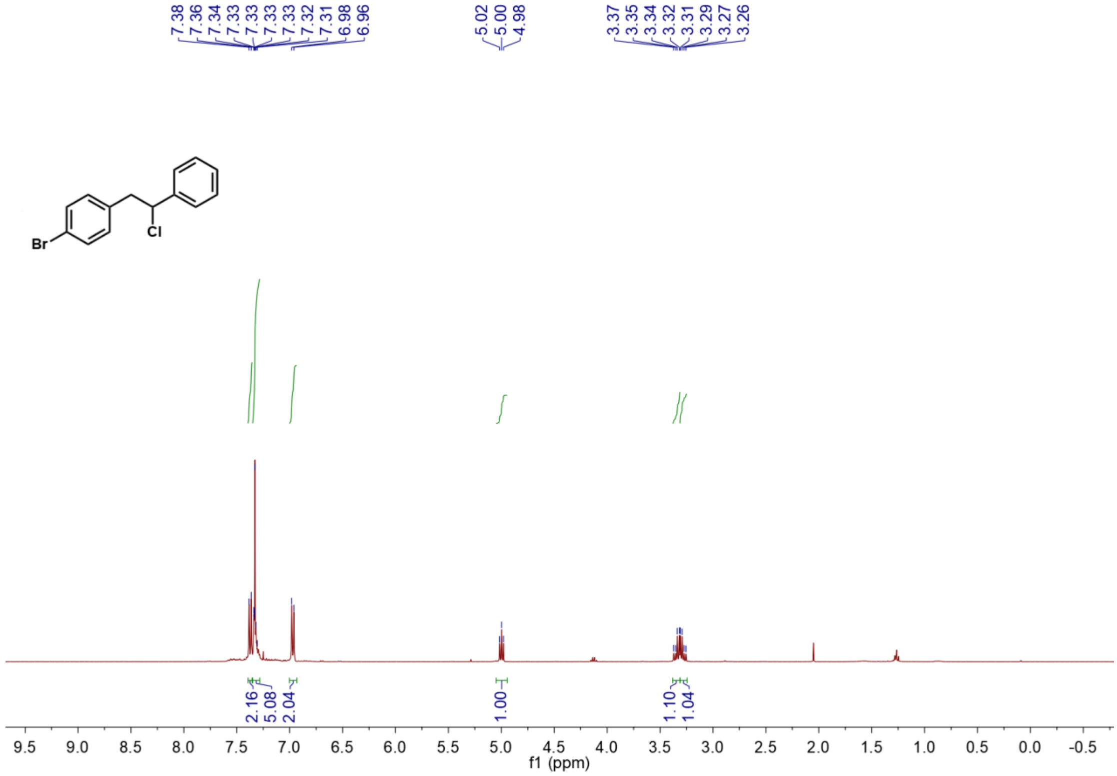

^{1}H NMR spectra (400 MHz, $CDCl_3$) of **36**.

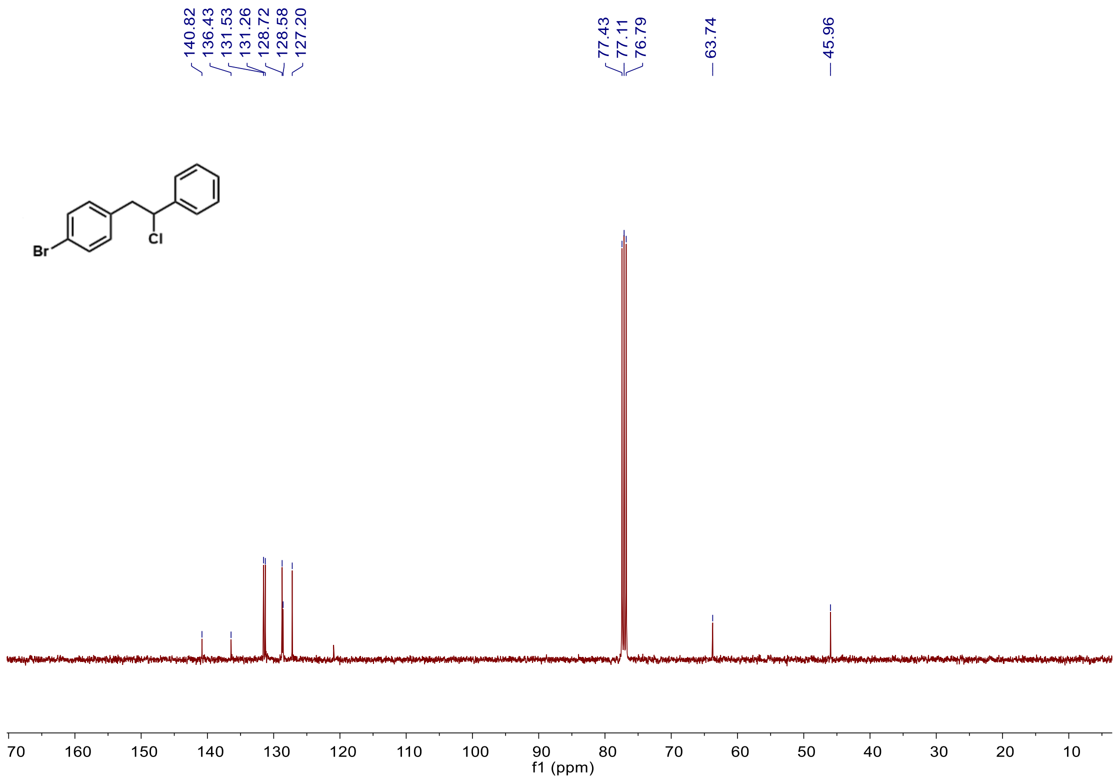

^{13}C NMR spectra (101 MHz, $CDCl_3$) of **36**.

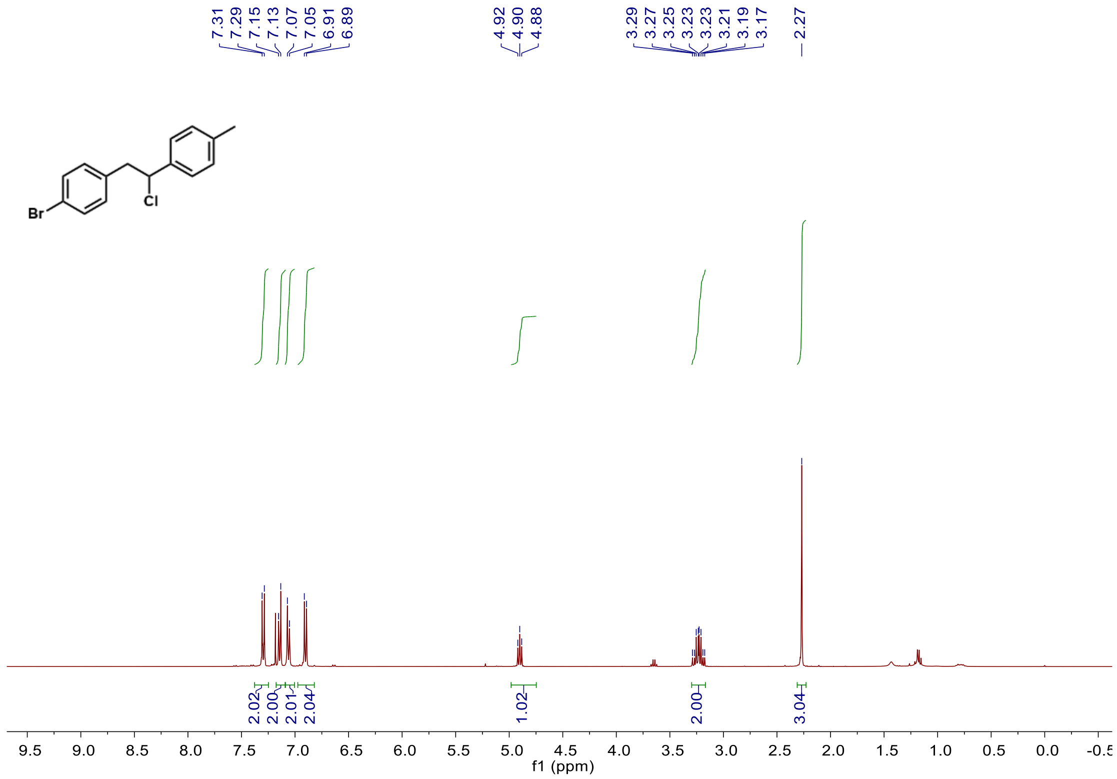

^{1}H NMR spectra (400 MHz, $CDCl_3$) of **37**.

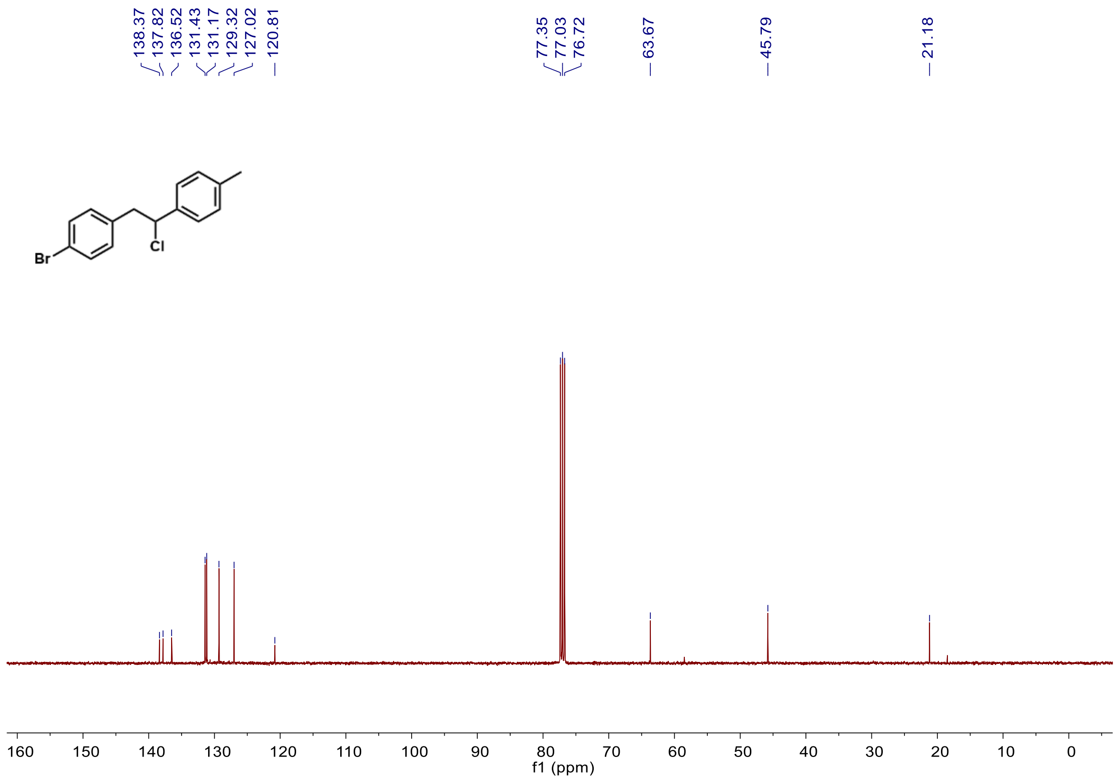

^{13}C NMR spectra (101 MHz, $CDCl_3$) of **37**.

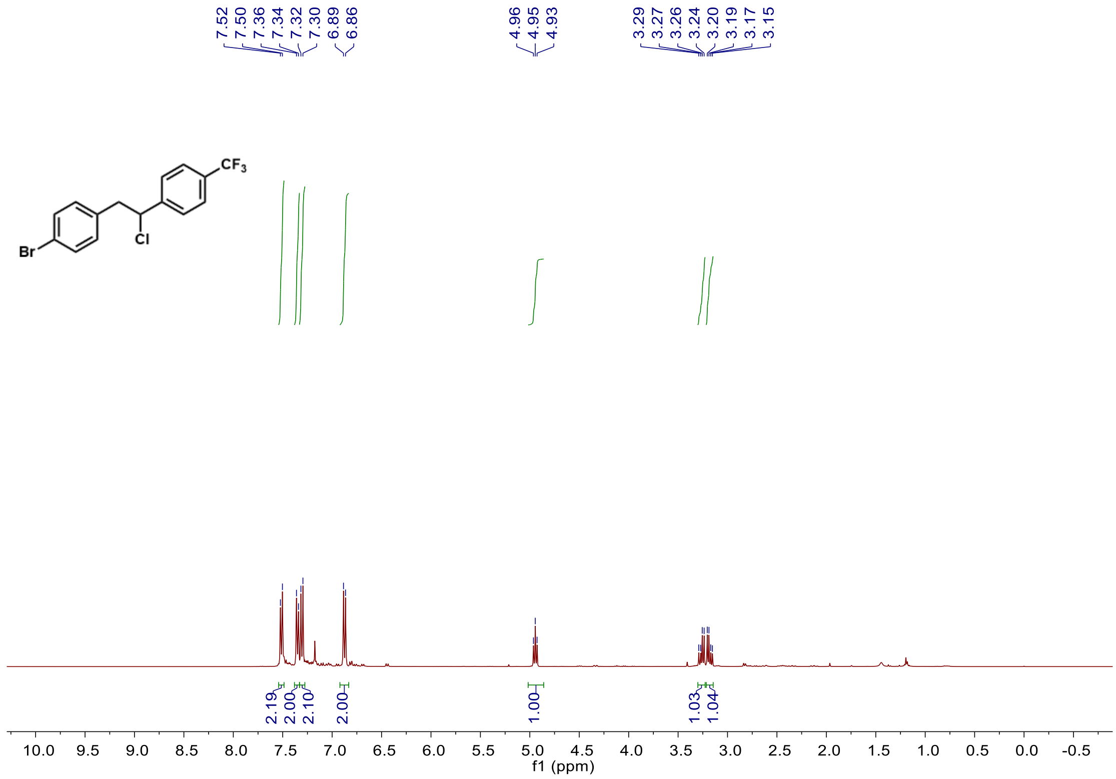

^{1}H NMR spectra (400 MHz, $CDCl_3$) of **38**.

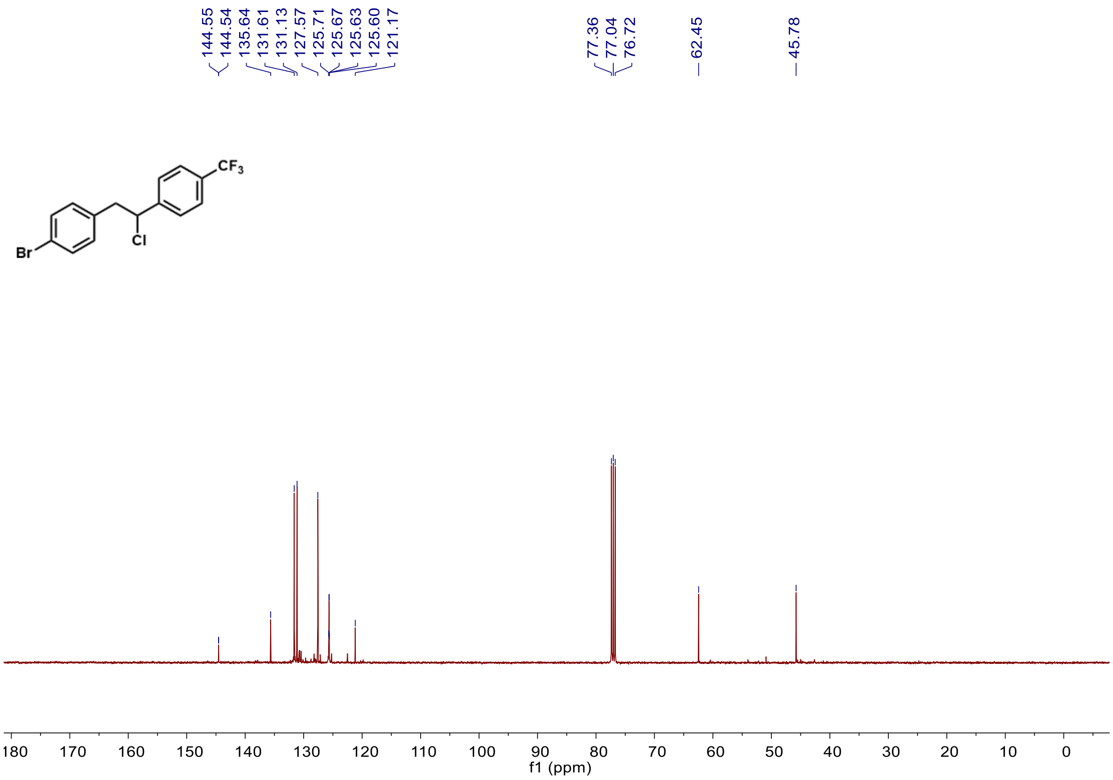

^{13}C NMR spectra (101 MHz, $CDCl_3$) of **38**.

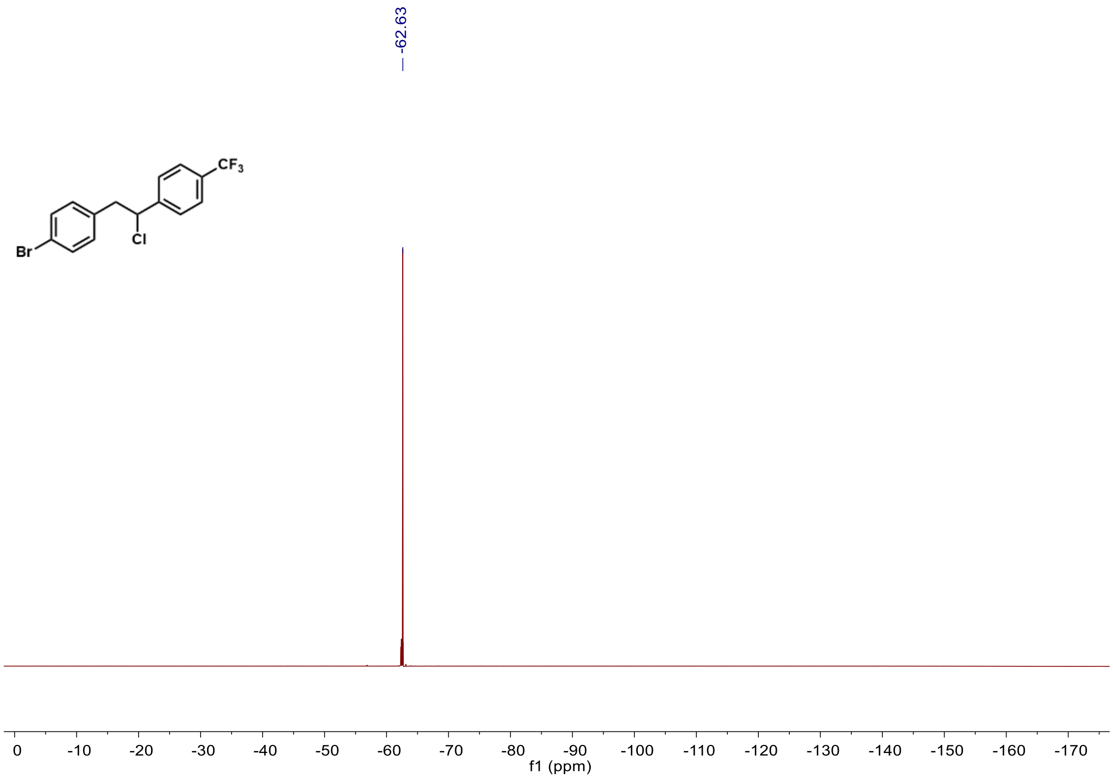

^{19}F NMR spectra (376 MHz, $CDCl_3$) of **38**.

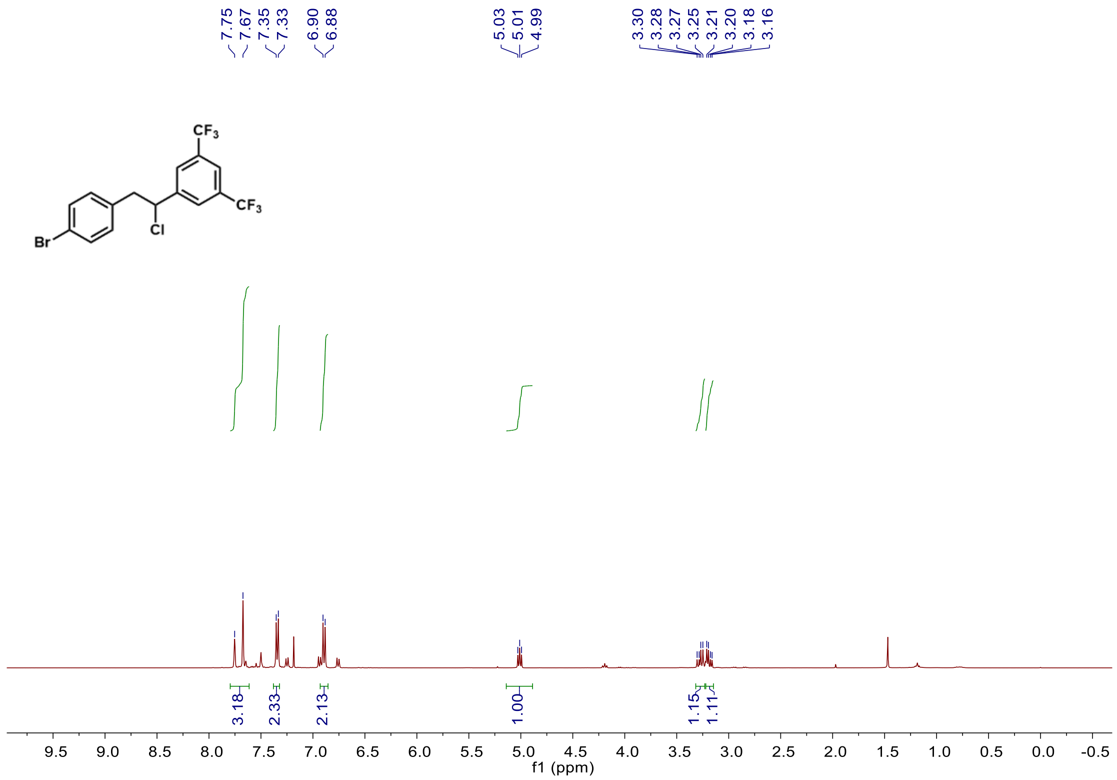

^{1}H NMR spectra (400 MHz, $CDCl_3$) of **39**.

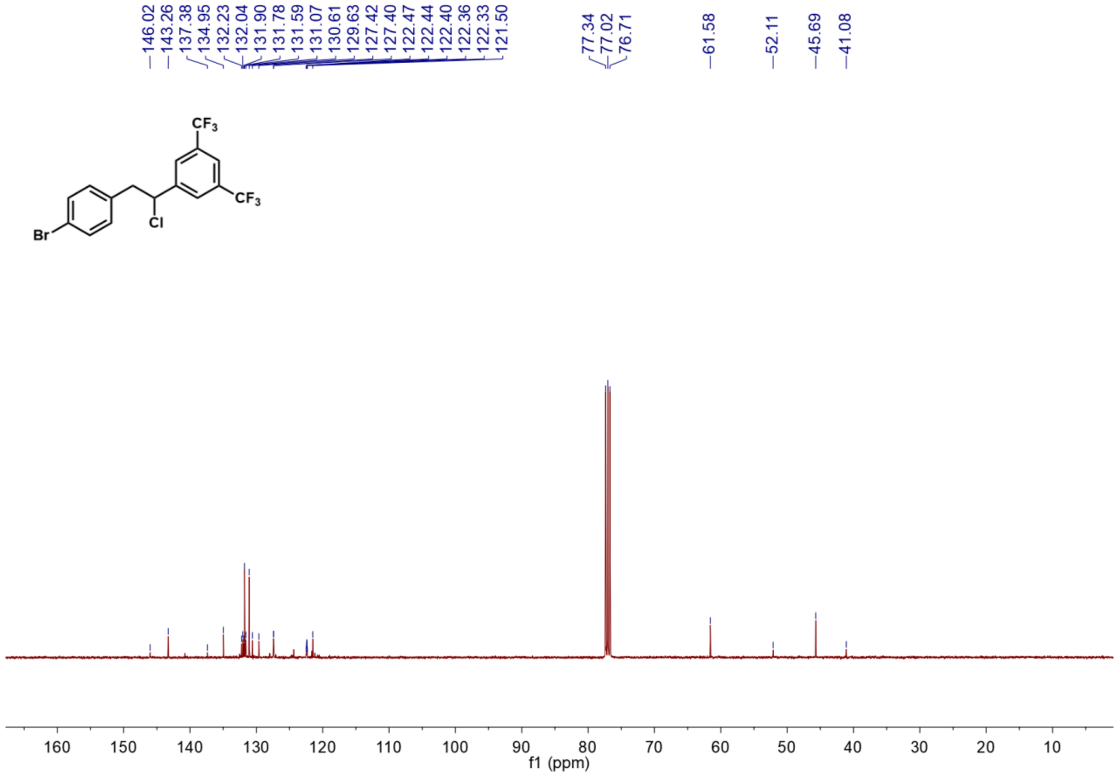

^{13}C NMR spectra (101 MHz, $CDCl_3$) of **39**.

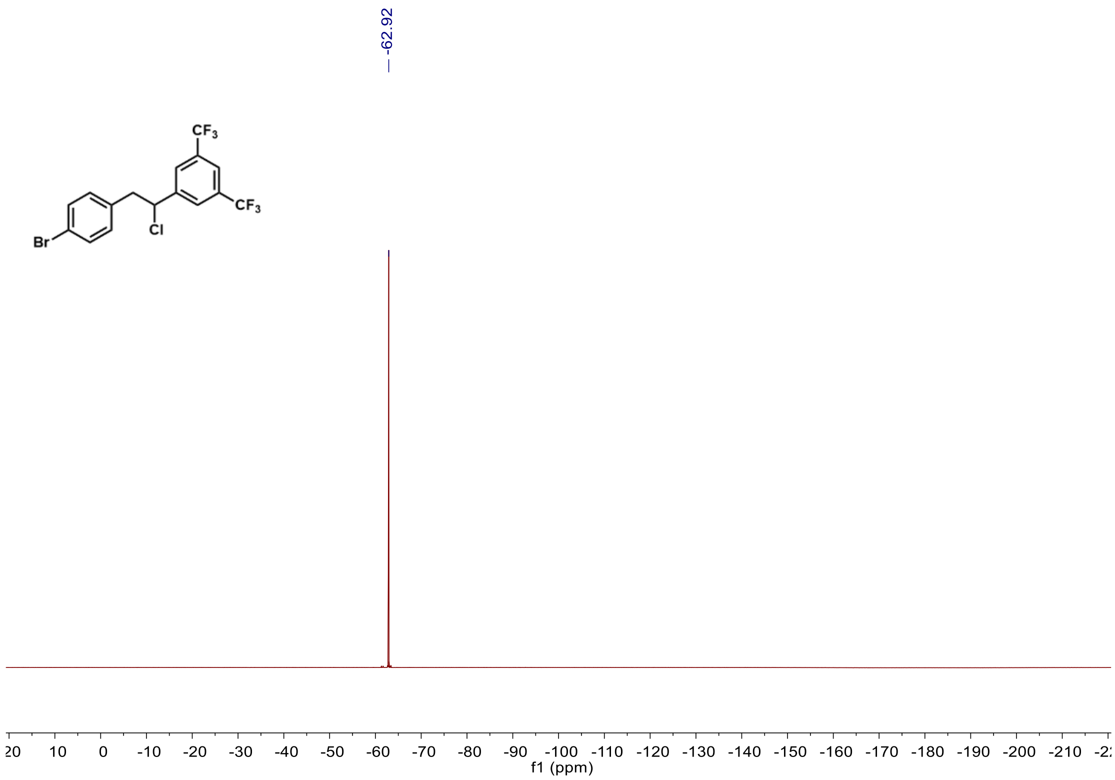

^{19}F NMR spectra (376 MHz, $CDCl_3$) of **39**.

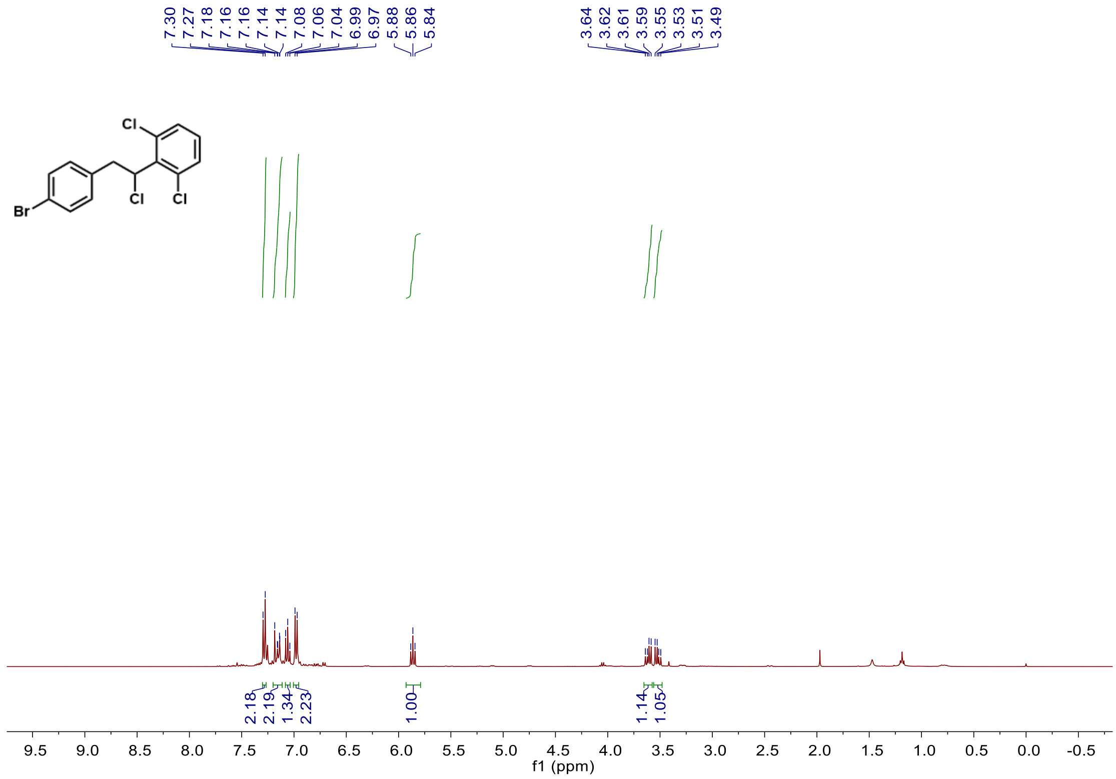

^{1}H NMR spectra (400 MHz, $CDCl_3$) of **40**.

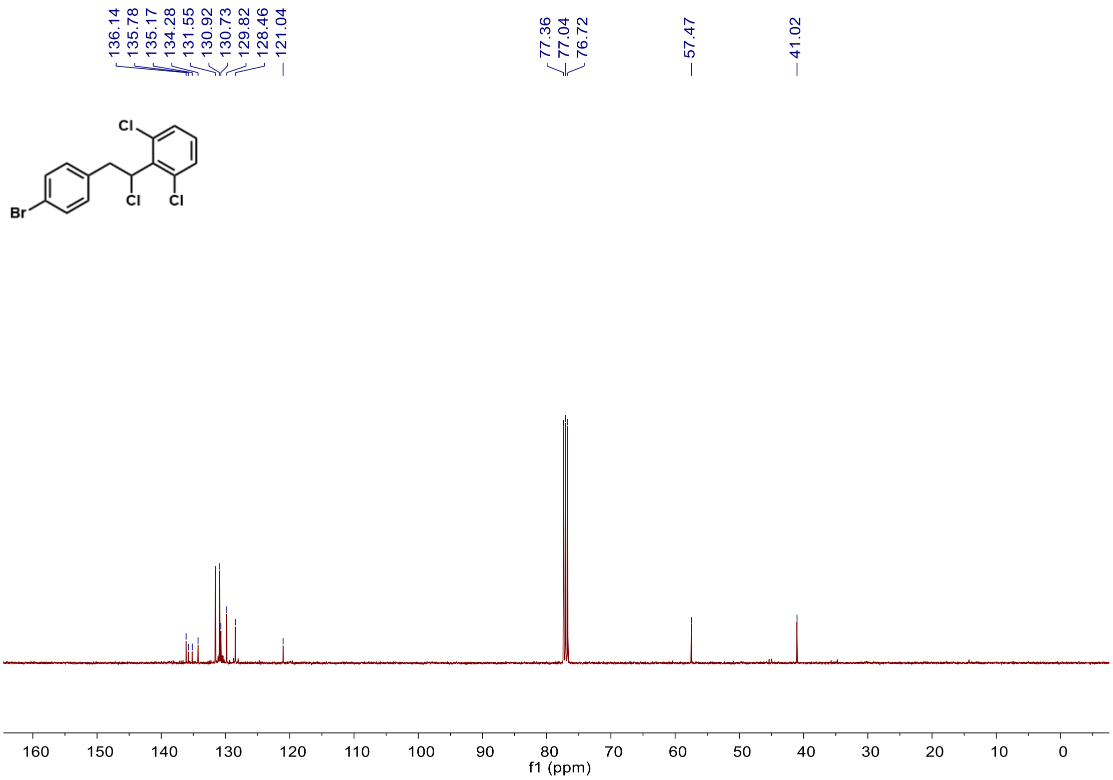

^{13}C NMR spectra (101 MHz, $CDCl_3$) of **40**.

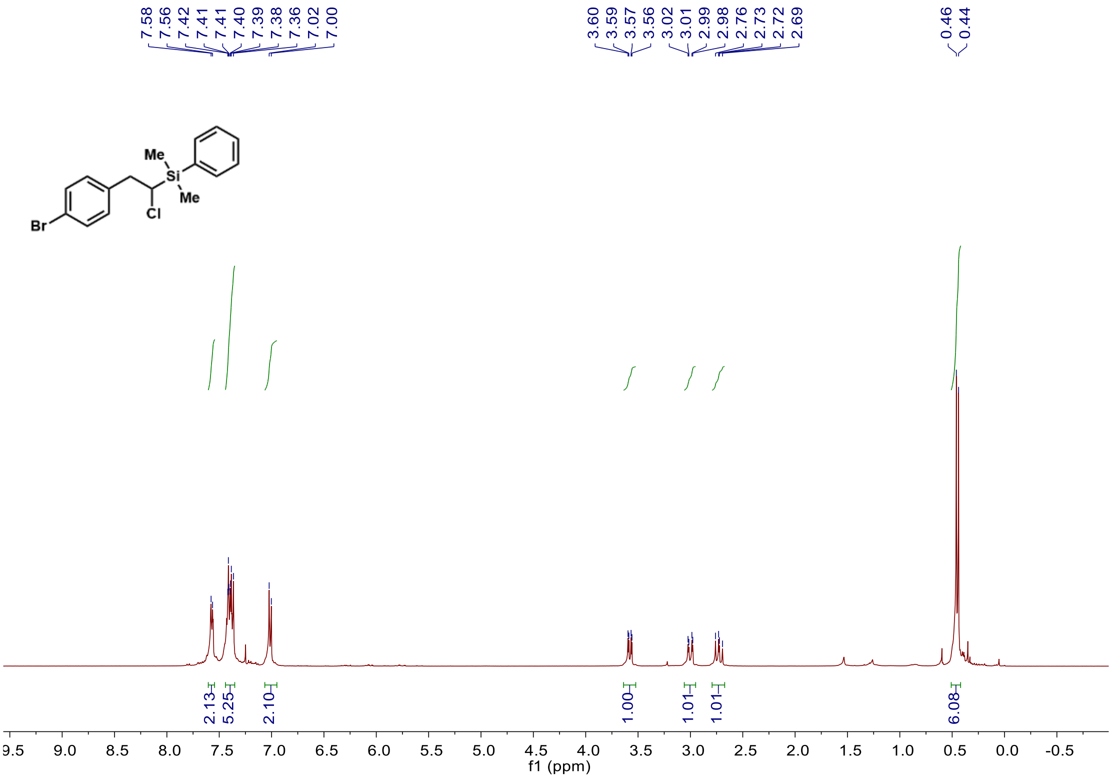

^{1}H NMR spectra (400 MHz, $CDCl_3$) of **41**.

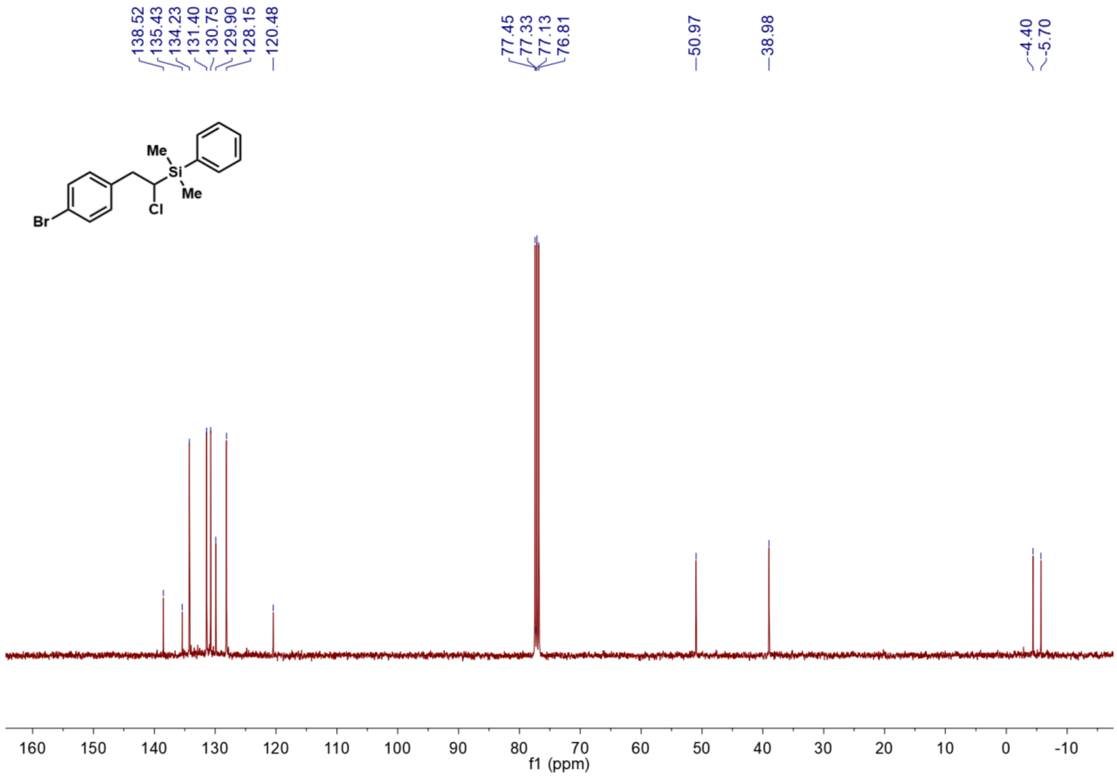

^{13}C NMR spectra (101 MHz, $CDCl_3$) of **41**.

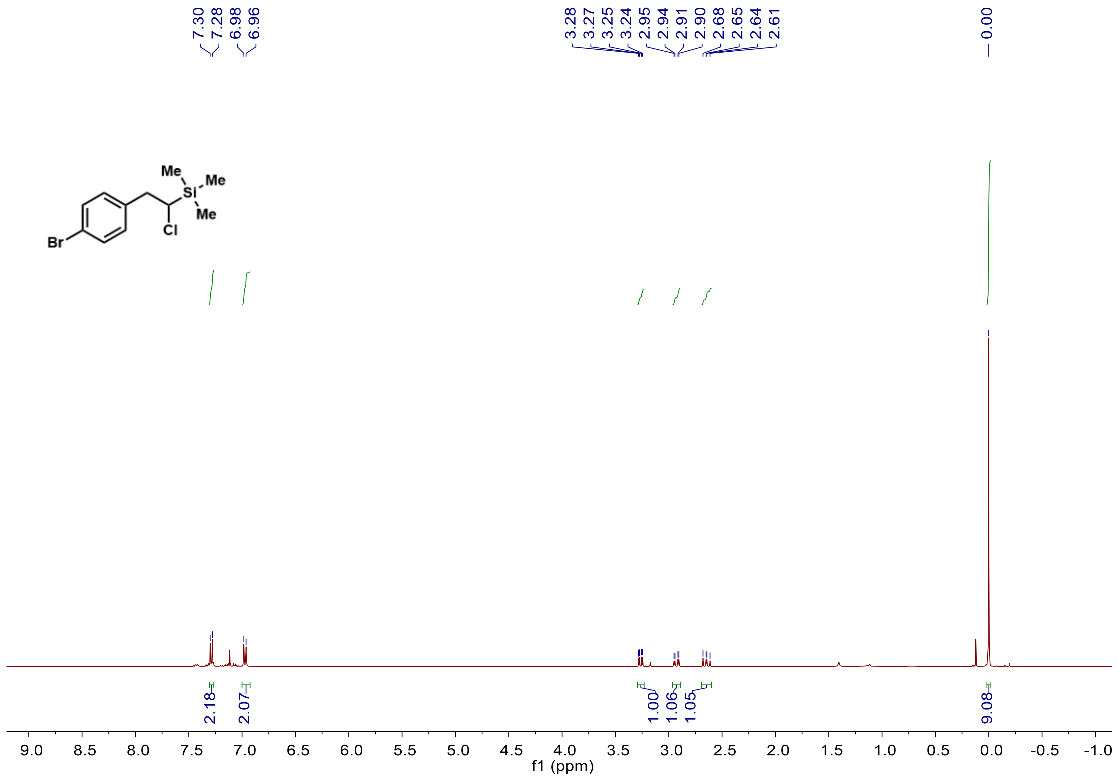

^{1}H NMR spectra (400 MHz, $CDCl_3$) of **42**.

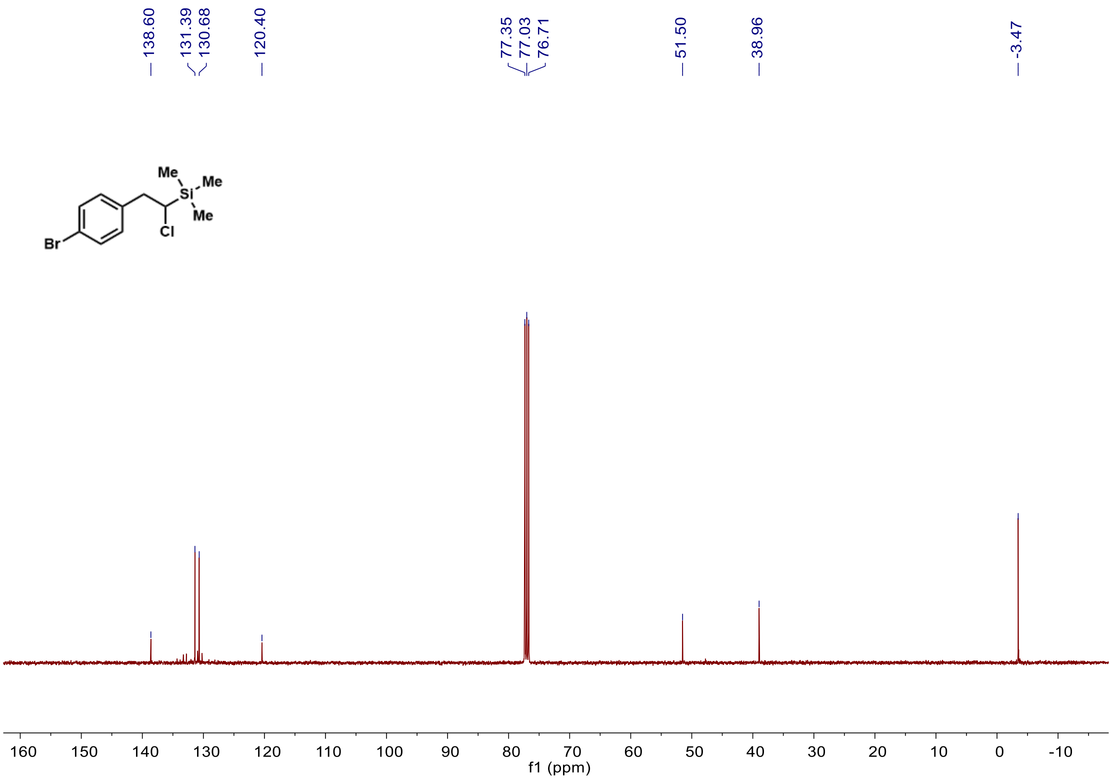

^{13}C NMR spectra (101 MHz, $CDCl_3$) of **42**.

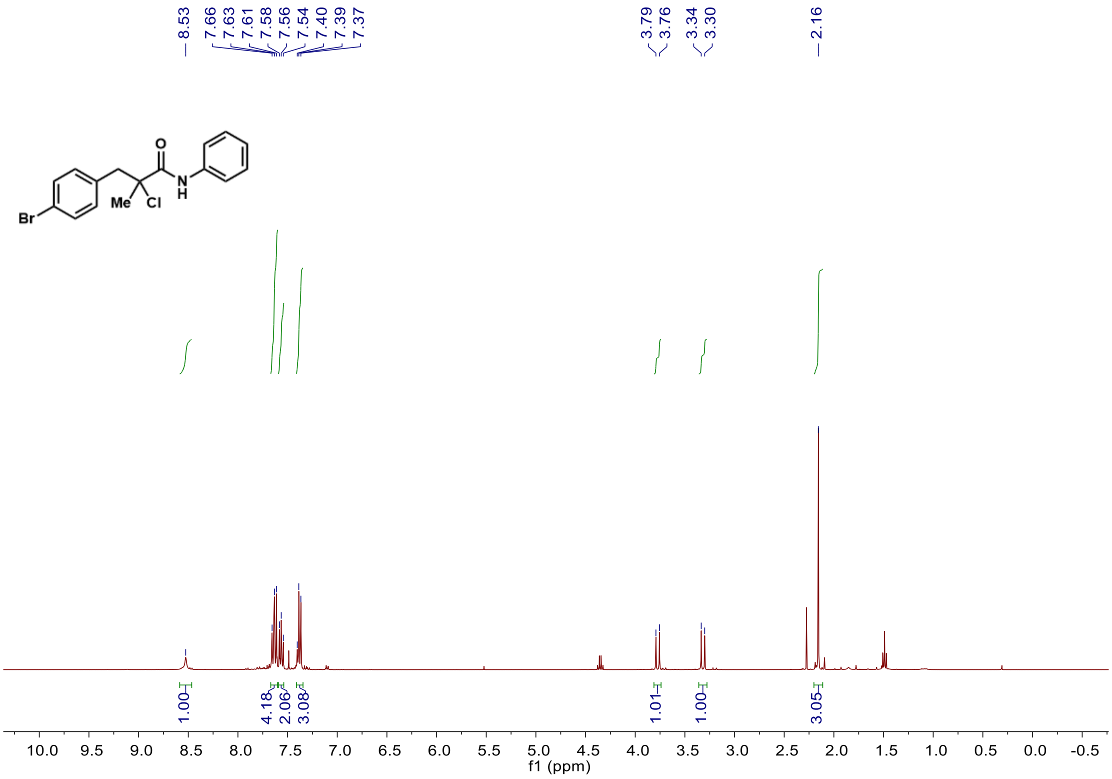

^{1}H NMR spectra (400 MHz, $CDCl_3$) of **43**.

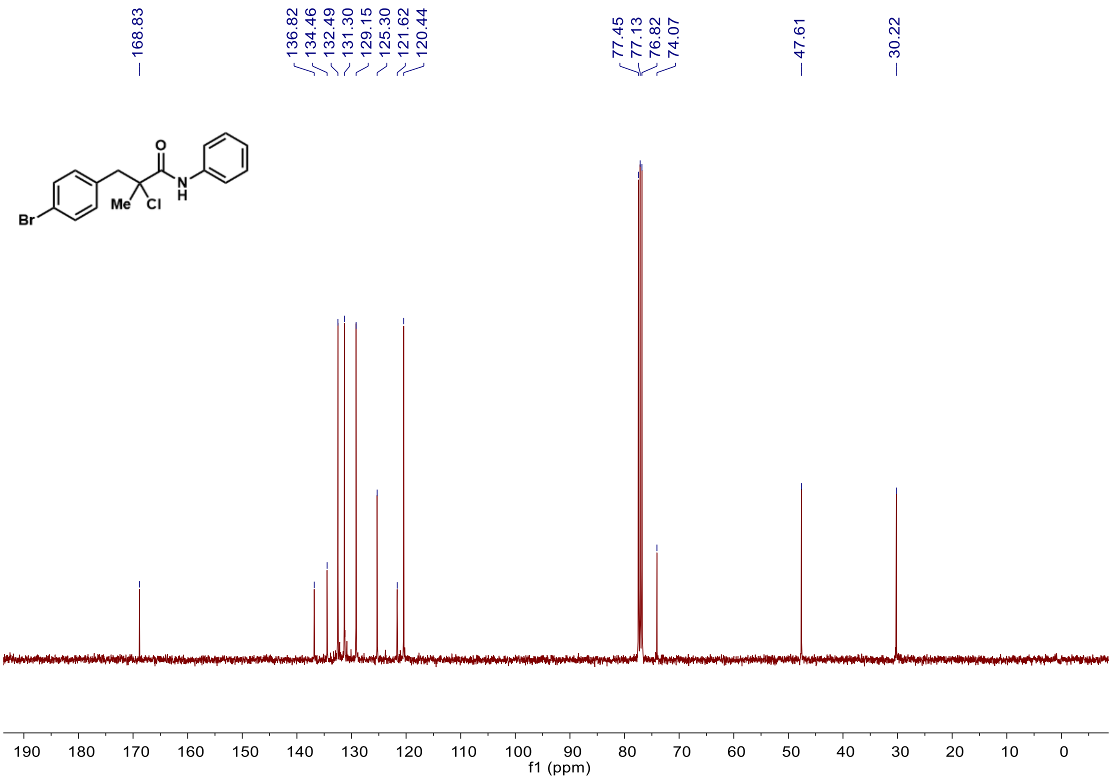

^{13}C NMR spectra (101 MHz, $CDCl_3$) of **43**.
